# Enhanced Cytotoxic Activity of PEGylated Curcumin Derivatives: Synthesis, Structure–Activity Evaluation, and Biological Activity

**DOI:** 10.3390/ijms24021467

**Published:** 2023-01-11

**Authors:** Dawid Lazewski, Malgorzata Kucinska, Edward Potapskiy, Joanna Kuzminska, Lukasz Popenda, Artur Tezyk, Tomasz Goslinski, Marcin Wierzchowski, Marek Murias

**Affiliations:** 1 Department of Chemical Technology of Drugs, Poznan University of Medical Sciences, Grunwaldzka 6 Street, 60-780 Poznan, Poland; 2 Department of Toxicology, Poznan University of Medical Sciences, Dojazd 30 Street, 60-631 Poznan, Poland; 3 Department of Pharmaceutical Chemistry, Poznan University of Medical Sciences, Grunwaldzka 6 Street, 60-780 Poznan, Poland; 4 NanoBioMedical Centre, Adam Mickiewicz University, Wszechnicy Piastowskiej 3 Street, 61-614 Poznan, Poland; 5 Department of Forensic Medicine, Poznan University of Medical Sciences, Rokietnicka 10 Street, 60-806 Poznan, Poland; 6 Center for Advanced Technology, Adam Mickiewicz University, Uniwersytetu Poznanskiego 10 Street, 61-614 Poznan, Poland

**Keywords:** curcumin, curcumin derivatives, PEGylation, hypoxia, bladder cancer

## Abstract

Curcumin has been modified in various ways to broaden its application in medicine and address its limitations. In this study, we present a series of curcumin-based derivatives obtained by replacing the hydroxy groups in the feruloyl moiety with polyethylene glycol (PEG) chains and the addition of the BF_2_ moiety to the carbonyl groups. Tested compounds were screened for their cytotoxic activity toward two bladder cancer cell lines, 5637 and SCaBER, and a noncancerous cell line derived from lung fibroblasts (MRC-5). Cell viability was analyzed under normoxic and hypoxic conditions (1% oxygen). Structure–activity relationships (SARs) are discussed, and curcumin derivatives equipped within feruloyl moieties with 3-methoxy and 4-{2-[2-(2-methoxyethoxy)ethoxy]ethoxy} substituents (5) were selected for further analysis. Compound **5** did not affect the viability of MRC-5 cells and exerted a stronger cytotoxic effect under hypoxic conditions. However, the flow cytometry studies showed that PEGylation did not improve cellular uptake. Another observation was that the lack of serum proteins limits the intracellular uptake of curcumin derivative **5**. The preliminary mechanism of action studies indicated that compound **5** under hypoxic conditions induced G2/M arrest in a dose-dependent manner and increased the expression of stress-related proteins such as p21/CIP1, phosphorylated HSP27, ADAMTS-1, and phosphorylated JNK. In summary, the results of the studies indicated that PEGylated curcumin is a more potent compound against bladder cancer cell lines than the parent compound, and derivative **5** is worthy of further investigation to clarify its mechanism of anticancer action under hypoxic conditions.

## 1. Introduction

In searching for new drugs and treatment strategies, compounds of natural origin have always played an important role. Phytochemicals offer scientists a vast library of chemical structures, which constitute a starting point for designing new compounds and modifying the original scaffold to improve their properties and desired activities. Numerous plant-derived compounds, such as vincristine, vinblastine, and paclitaxel, have been approved by the Food and Drug Administration (FDA) to treat different cancers [1]. Several other phytochemicals are currently in clinical trials to treat various cancers, including curcumin, resveratrol, lycopene, piperine, quercetin, and capsaicin [1]. Curcumin (CUR) is a naturally fat-soluble polyphenolic compound, initially isolated from the plant *Curcuma longa* (*Zingiberaceae* family). The pharmacological activity of *Curcuma longa* is mainly due to the presence of phenolic plant secondary metabolites named curcuminoids consisting of curcumin and two related agents, demethoxycurcumin and bisdemethoxycurcumin [2]. In the past, curcumin was used as a spice and in Ayurvedic medicine to treat different medical conditions, such as wound healing, gastrointestinal inflammation, cough, sinusitis, and indigestion [3]. Recent studies showed that curcumin exerted anti-inflammatory, pro-, and antioxidant [4], anticancer [5], and antibacterial [6] activity. Curcumin increases the expression of various genes and is involved in several signaling pathways, e.g., NF-κB [7], MAPK [8], JAK/STAT [9], WNT/β-catenin [10], Hippo [11], NOTCH [12], and Akt/mTOR [13] pathways. Moreover, curcumin can be used as a photosensitizer for anticancer and antibacterial photodynamic therapy (PDT) [3]. Curcumin is a very “popular” phytochemical, and in 2021–2023 alone, curcumin, as a search keyword, returned over 2000 results in the PUBMED database. Unfortunately, as with most phytochemicals, curcumin exhibits low bioavailability and unfavorable physicochemical parameters, rapid metabolism, and instability. Curcumin demonstrates pleiotropic activity; however, a broad spectrum of potential cellular targets proves to be both an advantage and a limitation. In the context of cancer diseases, it is mainly related to the possible lack of selectivity and the influence on the homeostasis of normal and neoplastic cells. Furthermore, in the literature, curcumin is presented as a PAINS (pan-assay interference compound), IMPS (invalid metabolic panaceas), and a poor lead compound when considering its pharmacokinetic and pharmacodynamic properties [14]. It should be emphasized, however, that scientists should not expect that one compound can fulfill all expectations. It is more important to understand the structure and mechanism of action to solve the potential drawbacks and limitations. Since curcumin is one of the compounds that have been studied for many years and much is known about its mechanism of action, curcumin, as a scaffold for designing new compounds, is still an attractive starting point.

As the emtansine story shows, even some drawbacks that block successful clinical development on the first attempt do not preclude later success. Emtansine, also known as DM1, is a derivative of maytansine that was originally isolated from an Ethiopian plant, *Maytenus ovatus*, and shown to have anticancer activity more potent than vinca alkaloids and paclitaxel [15]. However, the unfavorable toxicological profile has reduced interest in this drug candidate’s development. The situation reversed in 2008 when Lewis et al. showed that T-DM1, a conjugate of emtansine with trastuzumab—a monoclonal antibody that targets HER2 receptor—revealed its activity in a mouse xenograft model [15]. This antibody–drug conjugate (brand name Kadcyla^®^) was approved in 2013 by the European Union to treat adult patients with HER2-positive, locally advanced, or metastatic breast cancer and who had previously received trastuzumab and a taxane [16]. This example demonstrates that an appropriate modification can completely change the application of a problematic drug candidate. Therefore, despite numerous critical reports, the structure of curcumin can benefit medicinal chemistry.

**Figure 1:**
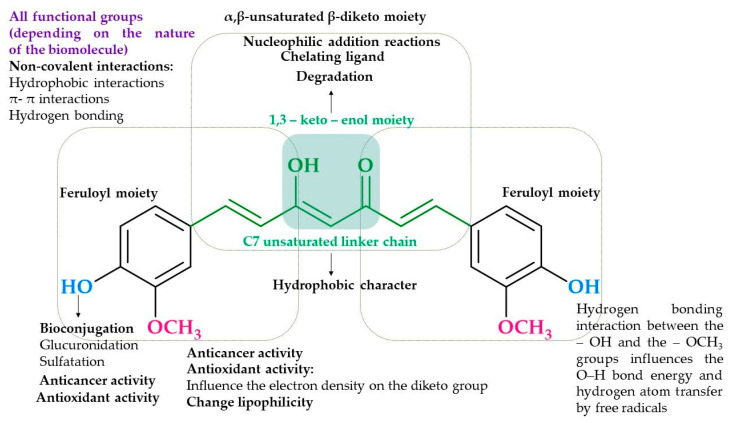
The curcumin functional groups of importance for biological activity. The figure was prepared following the references: [14,17].

In terms of the Structure–activity relationship, curcumin is a very interesting molecule that offers several possible structural modifications. There is a high probability that each functional group presented in the curcumin structure contributes to its reactivity, which translates into biological effects. The role of functional groups is summarized in Figure 1. As a result of the presence of two α,β-unsaturated carbonyl moieties that act as Michael acceptors for −SH groups, curcumin can form covalent bonds with proteins [17]. Additionally, two phenolic groups are involved in radical scavenging activity, and the 1,3-dicarbonyl motif acts as an excellent chelator of metal ions [14].

Inspired by nature, curcumin derivatives are promising structures that might overcome the limitations of the parental molecule. This work focuses on two chemical modifications of curcumin’s chemical structure by attaching the polyethylene glycol (PEG) chains and fluoroborination. PEGylation is widely accepted as a successful method of increasing hydrophilicity and the water solubility of compounds [18]. Polyethylene glycol polymers consist of linear or branched repeating ethylene glycol units [19]. PEG is nontoxic, biocompatible, hydrophilic, and FDA-approved for human use [20,21]. PEG is generally a low immunogenic agent; however, anti-PEG antibodies may appear after treatment with PEGylated drugs [22]. Although PEG should be used with caution because of the possibility of an allergic reaction, which was recently documented by observed post-vaccination hypersensitivity after using PEGylated lipid nanoparticles in COVID-19 mRNA vaccines [23], PEG still plays an essential role in pharmacy, food, and cosmetic technology. PEGylation can be used as a direct curcumin modification and a component of the delivery system, e.g., liposomes [24,25], micelles [26], or chitosan nanoparticles [27]. However, PEG chains very often have considerably higher mass (a few thousand Da) than the active compound and are connected via easily hydrolyzable bonds, such as esters, to act as prodrugs [28]. We have focused on short PEG chains (*n* = 3) and ether bonds, which are not susceptible to hydrolysis. While PEG chains might improve pharmacokinetic properties, adding the BF_2_ moiety to the dicarbonyl motif can effectively stop the keto-enol tautomerization, thus improving photophysical properties and increasing curcumin stability [29]. The addition of boron difluoride to the dicarbonyl motif within the curcumin structure can also improve some optical properties of which the most spectacular belong to an increase in the emission quantum yields, absorption coefficients, and shift of the absorption maxima to longer wavelengths [30,31]. Moreover, as shown in the previous studies, the presence of the BF_2_ group may also increase the cytotoxic effects on cancer cells [32].

The synthesized compounds were tested on human bladder cancer cell lines (5637 and SCaBER) and human lung normal fibroblast MRC-5 (noncancerous cell line). We analyzed the structure–activity relationship by comparing the activity of curcumin and isocurcumin and their BF_2_ adducts with or without short PEG chains. It should be highlighted that, to the best of our knowledge, there are no references to the cytotoxicity of isocurcumin against bladder cancer cells. Our research also demonstrated how the position of the methoxy group influences the phenolic ring’s hydroxyl group/PEG chains and the generated cytotoxic effect on the whole. In general, PEGylated compounds are significantly more active than curcumin. Experiments performed under hypoxic conditions (1% oxygen) showed that all compounds retain their activity. Based on the cytotoxicity test against cancer and normal cells and the activity under normoxic and hypoxic conditions, compound **5** was selected for further research. Interestingly, the PEGylation did not improve cellular uptake. Furthermore, we observed that the lack of serum proteins limits the intracellular uptake of curcumin derivative **5**. Compound **5,** in a concentration-dependent manner, induced G2/M phase arrest in 5637 cells. We also demonstrated that compound **5** under hypoxic conditions could increase the expression of stress-related proteins such as phosphorylated heat shock protein 27 (p-HSP27), ADAM metallopeptidase with thrombospondin type 1 motif 1 (ADAMTS-1), phosphorylated c-Jun *n*-terminal kinase (p-JNK), and p21/CIP1, and hypoxia-inducible factors (HIFs), HIF-1α and HIF-2α. To sum up, the results of the studies indicate that PEGylated curcumin compound **5** is more potent than the parent compound as an anticancer agent, exerts better cytotoxic activity under hypoxic conditions, and becomes an attractive scaffold for medicinal chemistry.

## 2. Results and Discussion

### 2.1. Synthesis

**Figure 2:**
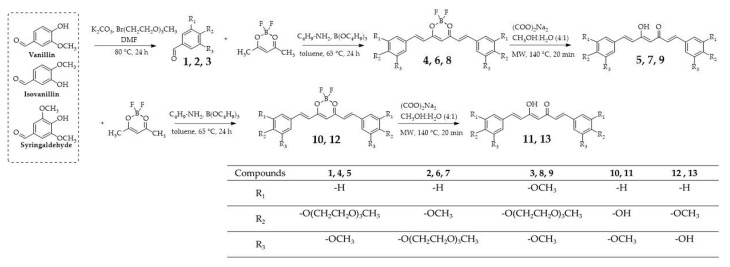
Synthesis of curcumin and isocurcumin derivatives (**4**–**13**), and intermediates for their synthesis (**1**–**3**). The aromatic aldehyde PEGylation (compounds **1**, **2**, and **3**) was performed using the nucleophilic substitution method. The borodifluorinated derivatives (compounds **4**, **6**, **8**, **10**, and **12**) were synthesized according to the method presented by Liu et al. [33], and derivatives with unmodified diketo moiety (compounds **5**, **7**, **9**, **11**, and **13**) were obtained by microwave hydrolysis developed by Abonia et al. [34]. Abbreviations: DMF, dimethylformamide; MW, microwave.

The method developed by Liu et al. [33] was applied to synthesize borodifluorinated curcumins and was subsequently accompanied by microwave hydrolysis developed by Abonia and co-workers [34] (Figure 2). It is worth noting that microwave hydrolysis, in contrast to the Pabon method [35], offers much higher yields and a more straightforward purification procedure. Previous studies on curcumin and its derivatives indicated an increase in their cytotoxic activity when hydroxyl groups were methylated. On one hand, this simple modification improved these compounds’ lipophilicity and permeability; on the other hand, however, some other effects of this approach should be highlighted, namely the fact that increased lipophilicity can be accompanied by limited solubility and increased metabolic rate, and can influence the bioavailability after oral administration. The improved hydrophilicity and lipophilicity of curcumin PEGylated derivatives with short chains demonstrate proper hydrophilic–lipophilic balance within the structure, which could lead to good bioavailability.

### 2.2. NMR Study

**Figure 3:**
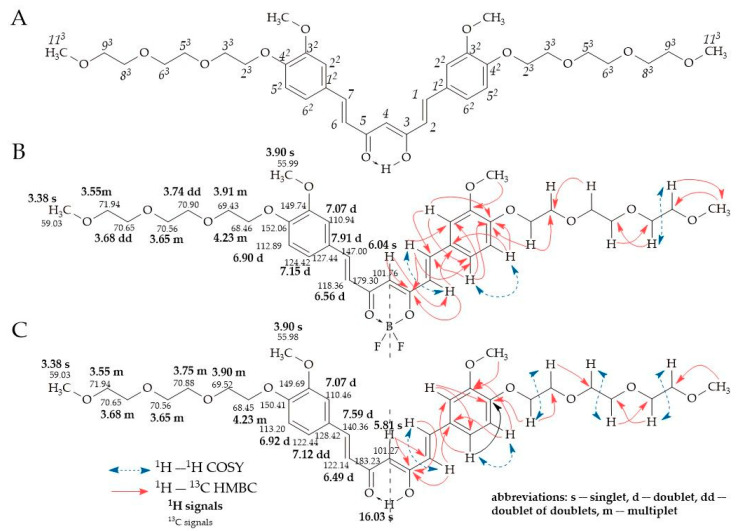
^1^H and ^13^C NMR data for compound **5** and its BF_2_ complex **4** in CDCl_3_. (**A**) Numbering of atoms within curcumin system; (**B**) Annotated ^1^H and ^13^C NMR signals for **4**; and (**C**) Annotated ^1^H and ^13^C NMR signals for compound **5**. Chemical shift values: ^1^H NMR (bold text) and ^13^C NMR (normal text) are expressed in ppm. Key ^1^H–^13^C HMBC and ^1^H–^1^H COSY correlations are marked with red full-line arrows and blue dashed double arrows, respectively.

The identity of the obtained compounds was confirmed by 1D ^1^H and ^13^C NMR techniques. In order to assign the observed signals in the 1D NMR experiments to the individual structural elements of the molecules, 2D correlation experiments (^1^H–^1^H COSY, ^1^H-^13^C HSQC, and ^1^H-^13^C HMBC) were also carried out. In Figure 3, annotated ^1^H and ^13^C NMR data for compound **5**, its BF_2_ complex **4**, and applied atom position numbering are presented.

According to NMR data, all investigated curcumin molecules revealed enolic forms. The chemical structure of compound **5** was confirmed by the presence of two signals with the integration of one proton for each of them: the first singlet appeared at 5.81 ppm representing position 4 with the CH group, and the second one (broad) at 16.03 ppm corresponded to the OH group (the result of tautomerization). The proton signal representing the CH group in position 4 of complex **4** appeared at 6.04 ppm. The presence of carbonyl groups in positions C3 and C5 was confirmed in ^13^C NMR by one signal at 183.23 ppm and 179.30 ppm, for compounds **5** and **4**, respectively. Two structural vinylene fragments -HC=CH- for compound **5** were represented in the ^1^H NMR spectrum by the signals of hydrogen and carbon atoms in positions 1, 2, 6, and 7. In ^1^H NMR, atoms H1 and H7 appear as a doublet at 7.59 ppm, whereas H2 and H6 appear as a doublet at 6.49 ppm. The protons H1-H2 and H6-H7 were present in *trans* configuration, which was confirmed by the ^3^*J*_H,H_ constant ca. 16 Hz. In the ^13^C NMR spectrum, the signal corresponding to C1 and C7 appeared at 140.36 ppm, whereas the signal at 122.14 ppm represented atoms C2 and C6. The presence of the -BF_2_ group caused a signal shift in the corresponding structural fragment of compound **4**. The doublets of H1(H7) and H2 (H6) were observed at 7.91 ppm and 6.56 ppm, respectively, whereas the carbon signals of C1(C7) and C2(C6) appeared at 147.00 ppm and 118.36 ppm, respectively. In the ^1^H NMR spectrum, the substituted benzene rings of compounds **5** and **4** were represented by three multiples. In the spectrum of compound **5**, the following signals, 7.07 ppm doublet, 6.92 ppm doublet, and 7.12 ppm doublet of doublets, were noted for H2^2^, H5^2^, and H6^2^, respectively, whereas for the corresponding atoms within complex **4**, a doublet at 7.07 ppm, a doublet at 6.90 ppm, and a doublet at 7.15 ppm, were observed. In the ^13^C NMR spectrum of compound **5**, ring C1^2^-C6^2^ was represented by peaks with values of 128.42 ppm, 110.46 ppm, 149.69 ppm, 150.41 ppm, 113.20 ppm, and 122.44 ppm, whereas carbon signals C1^2^-C6^2^ for complex **4** were observed at 127.44 ppm, 110.94 ppm, 149.74 ppm, 152.06 ppm, 112.89 ppm, and 124.42 ppm. The proton signals of methoxyl groups at position 3^2^ appeared as a singlet at 3.90 ppm for both studied curcuminoids. The locations of these carbon group signals appeared in the vicinity of 55.99 ppm for the complex and 55.98 ppm for free curcumin. The polyether chains protons of compound **5** in positions 2^3^,3^3^, 5^3^, 6^3^, 8^3^, 9^3^, and 11^3^ were represented by multiplets at 4.23 ppm, 3.90 ppm, 3.75 ppm, 3.65 ppm, 3.68 ppm, 3.55 ppm, and a singlet at 3.38 ppm, respectively. In the ^13^C NMR spectrum, the corresponding carbon signals for C2^3^, C3^3^, C5^3^, C6^3^, C8^3^, C9^3^, and C11^3^ appeared at 68.45 ppm, 69.52 ppm, 70.88 ppm, 70.56 ppm, 70.65 ppm, 71.94 ppm, and 59.03 ppm, respectively. The signals of H2^3^, H3^3^, H5^3^, H6^3^, H8^3^, H9^3^, and H11^3^ within complex **4** were represented by multiplets at 4.23 ppm, 3.91 ppm, 3.65 ppm, 3.55 ppm, doublets of doublets at 3.74 ppm and 3.68 ppm, and a singlet at 3.38 ppm, respectively. In the ^13^C NMR spectrum, the corresponding carbon signals for C2^3^, C3^3^, C5^3^, C6^3^, C8^3^, C9^3^, and C11^3^ appeared at 68.46 ppm, 69.43 ppm, 70.90 ppm, 70.56 ppm, 70.65 ppm, 71,94 ppm, and 59.03 ppm, respectively. All of the NMR spectra (Figures S1–S40) are presented in the Supplementary Materials.

### 2.3. Mass Spectroscopy

**Figure 4:**
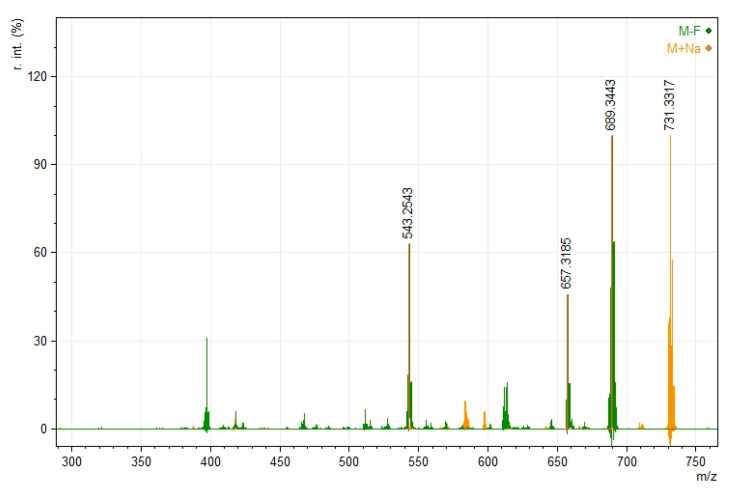
Mass spectrum of compound **4** with the presentation of fragmentations; [M+Na]^+^ sodium adduct with molecular ion in yellow, and [M-F]^+^ molecular ion without fluorine anion in green.

In the mass spectrometry experiments, the fragmentation pathways were studied. The difluoroborinated complexes lost a fluorine ion during ionization, prompting further fragmentation into smaller ions. It is worth noting that ions containing sodium appeared stable, and their fragmentation was almost negligible, as shown in Figure 4.

The same phenomenon was observed for non-fluoroborinated curcumin **5**. The remarkable stability of the sodium adduct to fragmentation was noted (Figure 5). The mass spectra for other compounds are presented in the Supplementary Materials (Figures S41–S48).

### 2.4. UV–Vis Study

**Figure 6:**
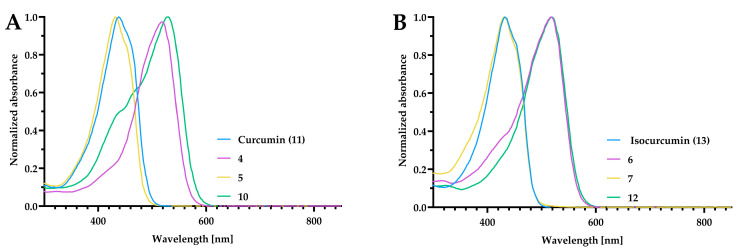
The normalized absorption spectra in DMSO of curcumin and its derivatives (**A**) and isocurcumin and its derivatives (**B**). The compounds were dissolved at the concentration of 10 µM in DMSO, and DMSO was used as a blank. The spectra were recorded using a standard cuvette port for single sample absorbance with a Tecan Infinite M PLEX microplate reader.

In general, the absorbance maxima in the UV–vis spectra of the curcumin derivatives were broad, with λ_max_ around 420 nm for the curcumin and isocurcumin, and 500 nm for their BF_2_ complexes (Figure 6). The spectra recorded in chloroform were added to the Supplementary Materials (Figures S49–S53).

### 2.5. Biological Activity of Tested Compounds

#### 2.5.1. Cytotoxic Activity of Tested Compounds

Several studies have demonstrated that PEG incorporation into the curcumin structure improves its pharmacological activity. As reported by Cheng and co-workers, novel PEGylated curcumin (Curc-mPEG454) synthesized by conjugating two low molecular weight PEGs (mPEG454) via β-thioester bonds to curcumin exhibited anti-inflammatory and antioxidant activity [36]. Li et al. presented that curcumin PEGylated with long chains improves water solubility and anticancer activity against human pancreatic cell lines [37]. Moreover, this curcumin derivative interacted synergistically with gemcitabine to sensitize pancreatic cancer cells to apoptosis.

**Figure 7:**
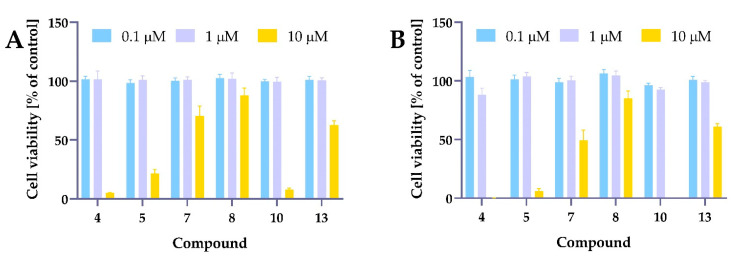
The preliminary data indicated the most promising structure for further research. Panel (**A**) presents the cytotoxic effects of compounds **4**, **5**, **7**, **8**, **10**, and **13** after treatment lasting 24 h, while panel (**B**) presents cell viability for 48 h. The 5637 cells were treated with compounds **4**, **5**, **7**, **8**, **10,** and **13** at a concentration of 0.1 µM, 1 µM, and 10 µM for 24 h and 48 h. Cell viability was measured by MTT assay.

Preliminary cytotoxicity studies were performed for compounds **4**, **5**, **7**, **8**, **10**, and **13** against 5637 cells (Figure 7). The cells were incubated with the test compounds at three different concentrations of 0.1 µM, 1 µM, and 10 µM for 24 h and 48 h. Cell viability was measured using the MTT assay. As presented in Figure 7, most of the tested compounds decreased cell viability at the highest dose. Since our research aimed to develop structures showing activity higher than curcumin, we assumed that the compounds should be active below 10 µM concentration.

**Figure 8:**
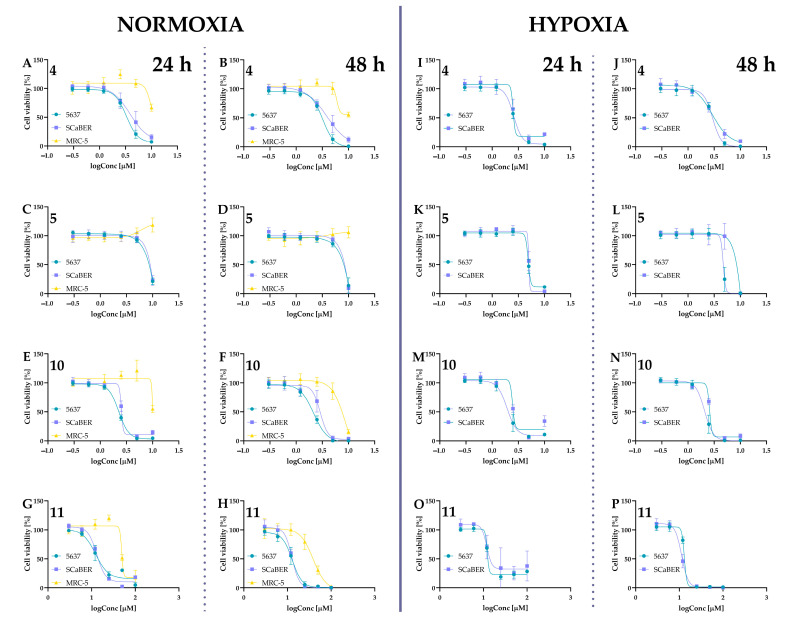
The cytotoxic effect of compounds **4**, **5**, **10**, and **11** on two human bladder cancer cell lines (5637, SCaBER), and a human noncancerous lung fibroblast cell line (MRC-5) under normoxic conditions and cancer cell lines also under hypoxic conditions (1% of oxygen). Panels (**A**–**H**) present data for normoxic conditions: panels (**A**,**B**) for compound **4**; panels (**C**,**D**) for compound **5**; panels (**E**,**F**) for compound **10**; panels (**G**,**H**) for compound **11**. Panels (**I**–**P**) present data for hypoxic conditions: panels (**I**,**J**) for compound **4**; panels (**K**,**L**) for compound **5**; panels (**M**,**N**) for compound **10**; panels (**O**,**P**) for compound **11**.Cells were treated with curcumin derivatives at a concentration of 0.3 µM, 0.6 µM, 1.2 µM, 2.5 µM, 5 µM, and 10 µM for 24 h and 48 h. The curcumin (**11**) was used at a concentration of 3 µM, 6 µM, 12 µM, 25 µM, 50 µM, and 100 µM. The DMSO was used as a control, and concentration in the cell culture medium did not exceed 0.1%. The H35 Hypoxystation (Don Whitley Scientific, Bingley, UK) created controlled and precise hypoxic conditions. For hypoxia experiments, cells, post-seeding, were placed in a hypoxia workstation 24 h before treatment. Cell viability was measured by MTT assay. Data are presented as mean values ± SD from at least two independent experiments.

**Figure 9:**
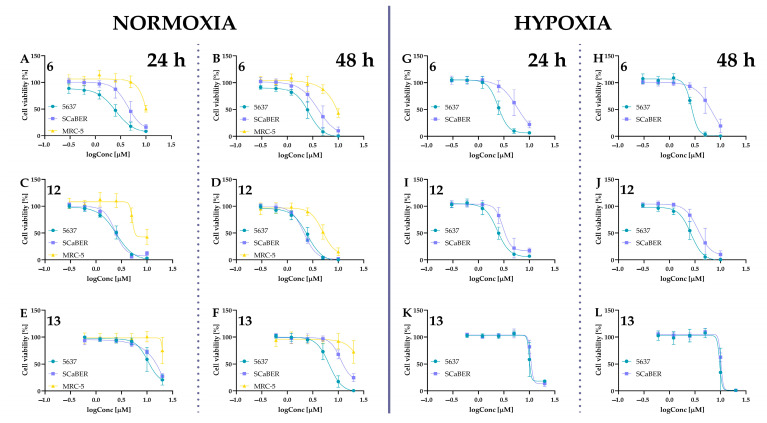
The dose-response curves plotted for compounds **6**, **12**, and **13**. Cell viability was measured using the MTT assay after 24 h and 48 h incubation under both normoxic and hypoxic (only for cancer cell lines) conditions. Panels (**A**–**F**) presents cell viability for 24 h and 48 h under normoxic conditions for compounds **6** (**A**,**B**); **12** (**C**,**D**), **13** (**E**,**F**). Panels (**G**–**L**) presents cell viability for 24 h and 48 h under hypoxic conditions for compounds **6** (**G**,**H**); **12** (**I**,**K**), **13** (**K**,**L**). Cells were treated with isocurcumin derivatives (**6** and **12**) at a concentration of 0.3 µM, 0.6 µM, 1.2 µM, 2.5 µM, 5 µM, and 10 µM for 24 h and 48 h. The isocurcumin (**13**) was used at a concentration of 0.6 µM, 1.2 µM, 2.5 µM, 5 µM, 10 µM, and 20 µM. The DMSO was used as a control. The H35 Hypoxystation (Don Whitley Scientific, Bingley, UK) created controlled and precise hypoxic conditions (1% of oxygen). Data are presented as mean values ± SD calculated from at least two independent experiments.

For further studies, compounds were tested in a concentration range of 0.3–10 µM to determine their IC_50_ values. Concentrations of 0.6–20 µM were used for isocurcumin, and the concentration range for curcumin, based on the literature data, was also higher (3–100 µM). The IC_50_ values are presented in Table 1, while the cell viability curves are presented in Figure 8 and Figure 9.

It should be highlighted that only one published data indicates the cytotoxic activity of isocurcumin. Deters et al. tested curcumin, isocurcumin, bisdesmethoxy-, diacetyl-, tetrahydro-, hexahydro-, octahydrocurcumin, vanillin, ferulic acid, and dihydroferulic acid against human peripheral blood mononuclear cell (PBMC) [38]. The authors demonstrated that curcumin and isocurcumin exerted the same activity against PBMC with an IC_50_ value of 2.8 µM for both compounds [38]. Our study found that curcumin and isocurcumin (**13**) have comparable IC_50_ values toward SCaBER cells, while isocurcumin decreased cell viability more efficiently against 5637 cells.

Our results showed that the compound most active against both cancer cell lines was compound **12**. However, this isocurcumin derivative with the BF_2_ moiety was also one of the most cytotoxic compounds against the normal MRC-5 cell line, with IC_50_ values of 9.63 µM and 5.80 µM for incubation lasting 24 h and 48 h, respectively. A Structure–activity relationship study showed that replacing the position of methoxy and hydroxy groups in BF_2_ derivatives (derivatives **10** and **12**) did not significantly change the cytotoxic activity against the cancer cell lines. However, the presence of the -BF_2_ moiety might decrease the selectivity toward noncancerous cells. On the other hand, compounds bearing PEG chains instead of the hydroxyl group exerted lower cytotoxic activity against MRC-5, even in the case of the BF_2_ complexes, while still exerting stronger anticancer activity compared to curcumin. Interestingly, in our assay, compound **7**, compared to its isomer compound **5**, was considered inactive (cell viability at a concentration of 10 µM was >60% after 24 h incubation). Thus, inserting the PEG into the isocurcumin structure is less beneficial than the curcumin derivative. As Laali and co-workers demonstrated, the BF_2_ adducts could fit nicely in the tunnel-like binding pocket of HER2 by establishing hydrophobic contacts leading to favorable docking energies. Thus, the interaction of BF_2_-possessing compounds with HER2 receptors can be involved in the cytotoxic activity of these compounds [32]. Based on the literature data, both cell lines used in our studies, 5637 and SCaBER, expressed the HER2 receptor [39]. The similar IC_50_ values calculated for our BF_2_ complexes toward 5637 and SCaBER cells could potentially result from this interaction. Interestingly, the activity slightly changed between 5637 and SCaBER cells by inserting the PEG chains. The BF_2_ derivatives with PEG chains were more active against 5637 cells than SCaBER cells.

**Figure 10:**
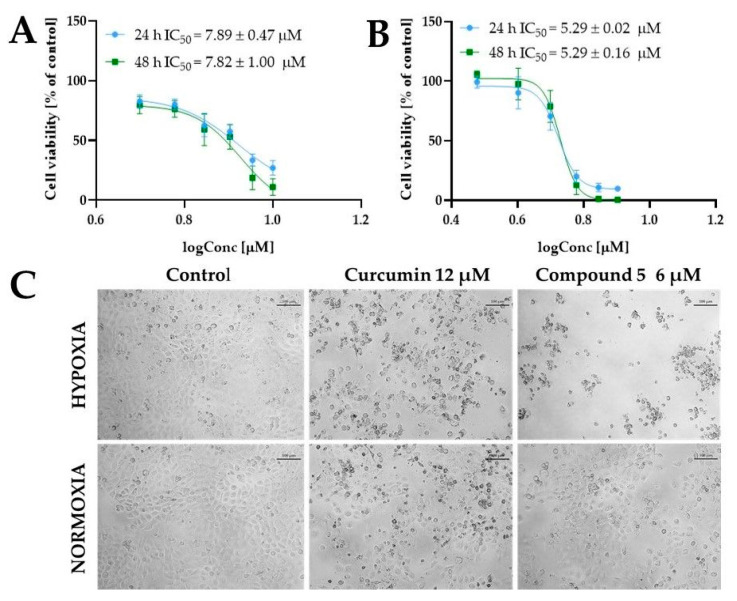
The cytotoxic effect of compound **5** against bladder cancer 5637 cells under normoxic and hypoxic (1% of oxygen) conditions. The cells were treated with compound **5** at a concentration of 5 µM, 6 µM, 7 µM, 8 µM, 9 µM, and 10 µM (normoxic conditions), and 3 µM, 4 µM, 5 µM, 6 µM, 7 µM, and 8 µM (hypoxic conditions) for 24 h (**A**) and 48 h (**B**). Cell viability was determined by MTT assay. Data are presented as mean values ± SD calculated from at least two independent experiments. Panel (**C**) presents the representative images of cell morphology after treatment with compound **5** at a concentration of 6 µM for 24 h under both hypoxic and normoxic conditions. The 5637 cells’ morphology after treatment with curcumin (12 µM) for 24 h is also shown for comparison. The images were taken with a DS-SMc digital camera attached to a Nikon Eclipse TS100 microscope. The scale bar corresponds to 100 µm.

Interesting properties were observed for curcumin derivative **5**. This compound shows a similar effect on tumor lines while not reducing the survival of the MRC-5 cells. Noteworthily, compound **4** decreased cell viability to 66% at a concentration of 10 µM, while for compound **5** at the same concentration, viability was approximately 100% (Figure 8). Moreover, under hypoxic conditions, the IC_50_ values of compound **5** were lower than normoxia. To obtain further insights into the cytotoxic activity of compound **5**, we determined the IC_50_ under normoxic and hypoxic conditions for a narrow concentration range. For normoxic conditions, compound **5** was used at a concentration of 5 µM, 6 µM, 7 µM, 8 µM, 9 µM, and 10 µM, while for hypoxia experiments, 5637 cells were treated with 3 µM, 4 µM, 5 µM, 6 µM, 7 µM, and 8 µM. The IC_50_ values calculated for -these concentration ranges are presented in Figure 10. We observed that compound **5** was more active under hypoxic conditions. The cell morphology shown in Figure 10C indicated that compound **5** exerted cytotoxic activity at lower concentrations under hypoxic conditions. We also compared the cellular morphology after incubation with curcumin at both conditions at a concentration close to its IC_50_ values.

#### 2.5.2. Cellular Uptake of PEGylated Curcumin

**Figure 11:**
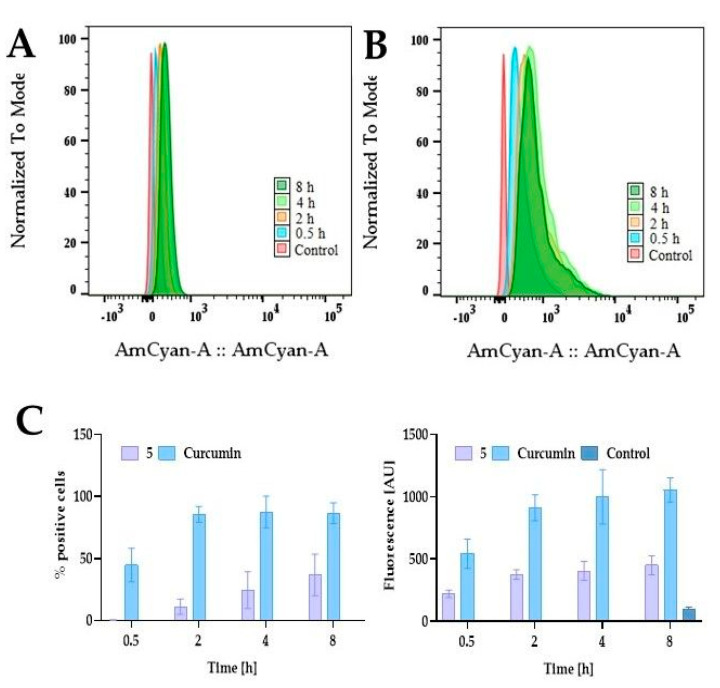
The time-dependent uptake of compound 5 and curcumin by 5637 cells. Based on the literature data, curcumin has extensive absorption around 408–430 nm and can emit fluorescence around 460–560 nm, while excitation and emission wavelengths of compound 5 are 436 nm and 530 nm, respectively. Panel (**A**) presents the representative histograms for compound **5**, and panel (**B**) for curcumin. Panel (**C**) presents quantitative analysis as the percent of positive cells and mean fluorescence intensity. The 5637 cells were treated with compound 5 and curcumin at a concentration of 10 µM for 0.5 h, 2 h, 4 h, and 8 h under standard cell culture conditions. The uptake was analyzed by flow cytometry (BD Lyric^TM^, Becton Dickinson, Franklin Lakes, NJ, USA) after excitation with a 405 nm laser and detection with a 505.5–550.5 band-pass filter (528/45). Data are presented as mean values ±SD from three experiments.

Since curcumin and compound **5** are fluorescent compounds, the fluorescence-based method can be used to determine cellular uptake. The maximum absorption of curcumin ranges between 408 and 430 nm, depending on the solvents [40]. Regarding the emission spectra, curcumin shows a stronger dependence on the solvent used, with the emission maximum ranging between 460 and 560 nm [40]. Curcumin derivative **5** is fluorochrome with excitation and emission wavelengths of 436 nm and 530 nm, respectively. To examine whether the curcumin PEGylation could improve intracellular uptake, we treated 5637 cells with curcumin and compound **5** at a concentration of 10 µM for 0.5 h, 2 h, 4 h, and 8 h and analyzed using flow cytometry. In the case of curcumin, we observed that after 0.5 h, approximately 30% of cells were positive and increased during the incubation to reach a plateau for the longer incubation time, with 80–90% positive cells for 2 h, 4 h, and 8 h (Figure 11). In contrast, we observed a different pattern for compound **5**. The number of positive cells is significantly lower than in curcumin, and we observed a time-dependent increase in the fluorescent signal. After incubation with curcumin lasting 8 h, we found over 85% positive cells. For compound **5**, the number of positive cells at this time was 36%.

**Figure 12:**
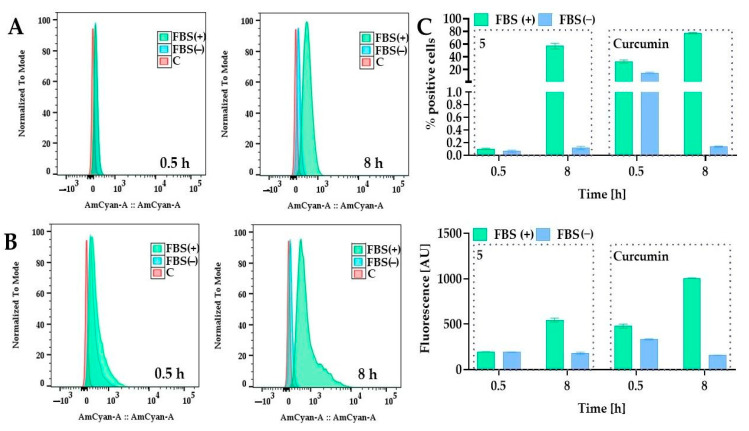
The influence of fetal bovine serum on curcumin and compound **5** uptake by 5637 cells. The uptake of compound **5** and curcumin was determined in a medium with FBS (10% *v/v*) and a serum-deprived medium (0% FBS). Panel (**A**) presents the representative histograms for compound 5 and panel (**B**) for curcumin. Panel (**C**) presents the results as a percentage of positive cells and mean fluorescence intensity. Compound **5** and curcumin were added to the cells at a concentration of 10 µM and incubated for 0.5 h and 8 h under standard cell culture conditions. The DMSO was used as a control. The fluorescence was measured by flow cytometry after excitation with a 405 nm laser and detection with a 505.5–550.5 nm band-pass filter (528/45). Abbreviations: fetal bovine serum, FBS; control, C.

We also observed that the presence of fetal bovine serum (FBS) is important for efficient cellular uptake (Figure 12). A previous study by Wang et al. showed that curcumin is more stable in a cell culture medium containing 10% fetal calf serum (FCS) than in human blood [41]. The authors demonstrated that less than 20% and 50% of curcumin decomposed within 1 h and 8 h in the presence of FCS, respectively, while in a serum-free medium, about 90% decomposed within 30 min [41].

Our results also showed that FBS might protect the curcumin from degradation in an aqueous environment. The same behavior was also observed for compound **5**. After 8 h, the fluorescence was diminished in the medium without FBS. These results can indicate that FBS can also stabilize compound **5**. In general, most of the information in the literature concerns the influence of PEG (usually long chains) on the stabilization and absorption of PEG nanoparticles in their structure. In contrast, information about the effects of short PEG chains attached covalently to small molecules on processes such as absorption is still missing. It is known that attaching PEG chains onto the liposome surface might block binding sites for proteins, thus creating a thermodynamic shield to protein diffusion, and might also be able to reduce nonspecific protein adsorption [42] and protect the surface from opsonization [43]. On the other hand, the increasing number of PEG chains can affect uptake by the cells [44,45]. As observed for nanoparticles, long PEG chains compared to short chains create a steric hindrance by reducing the electrostatic or van der Waals force, thus increasing the distance between particles and protein [46]. Although the uptake of PEGylated curcumin was less than that of curcumin, this cytotoxic effect was higher. The PEGylation increases the molecular mass, changes the number of H-bond acceptors (12 for compound **5**, and 6 for curcumin), and lipophilicity can impact the permeability coefficients, thus decreasing the uptake; however, this modification could offer protection from rapid degradation and, thus, in the end, achieve the better cytotoxic activity. Considering that PEG can interact with different proteins present in serum [47], compound **5** might not cross the membrane passively, and more specialized membrane transport may be involved in this process.

Furthermore, it should be highlighted that PEG can act as a P-glycoprotein (Pg-p) substrate and inhibitor [48,49,50]. Thus, as presented by Snyder, the attachment of methoxypolyethylene glycol (mPEG) to quinidine acts as a Pg-p inhibitor, and the intracellular level of quinidine was decreased compared to the free form [51]. Therefore, if the PEGylated curcumin can block P-glycoprotein and decrease its efflux, this strategy, even with reduced uptake, allows for a better biological effect. However, all of these hypotheses need detailed studies and further verification that exceed the work presented herein.

#### 2.5.3. Cell Cycle Distribution

**Figure 13:**
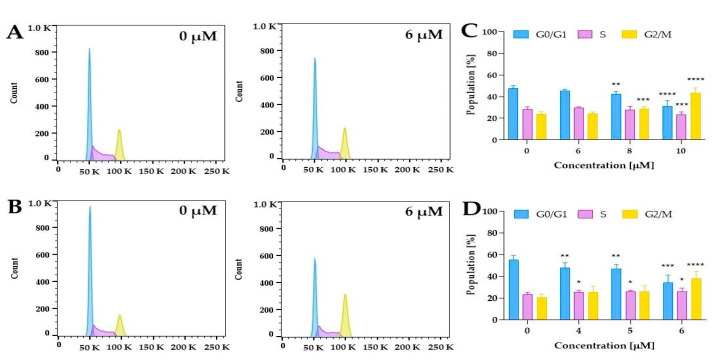
Cell cycle distribution after the treatment of 5637 cells with compound **5** under normoxic and hypoxic conditions. Cells were treated with compound **5** at a concentration of 6 µM, 8 µM, and 10 µM (normoxia), and 4 µM, 5 µM, and 6 µM (hypoxia) for 24 h and stained with propidium iodide. DNA content was analyzed by flow cytometry. Panels (**A**) and (**B**) present representative histograms for normoxia and hypoxia experiments, respectively. Panels (**C**) and (**D**) show the quantification of results for normoxic and hypoxic conditions, respectively. Results are presented as the mean ± standard deviation from three experiments. The asterisk indicates statistical significance * *p* < 0.05, ** *p* < 0.01, *** *p* < 0.001, and **** *p* < 0.0001 vs. the control group. Statistical significance was measured by one-way ANOVA Dunnett’s multiple comparisons tests.

The literature data showed that curcumin might affect cell cycle distribution in human bladder cancer cells [52,53,54,55]. Park et al. demonstrated that curcumin at a concentration of 12.5 µM significantly increased cell population in G2/M (64% cells) compared to control cells (33% cells) in the bladder cancer T24 cell line [53]. As we observed a decrease in cell survival at a different level for normoxia and hypoxia, we performed further experiments to clarify whether compound **5** might affect the cell cycle distribution. The findings revealed that compound **5** caused a significant increase in the number of cells in the G2/M phase in a dose-dependent manner, compared with control cells under both hypoxic and normoxic conditions (Figure 13C,D).

An interesting pattern was observed when comparing the hypoxia and normoxia experiments. For example, treatment with 6 µM increased the G2/M cell populations to 38% in 5637 cells, while in normoxia at this concentration, 24%, we did not observe a statistically significant increase in this phase (Figure 13C,D). These findings confirmed that compound **5** induced a G2/M dose-dependent cell cycle arrest in 5637 cells, which is modulated by oxygen concentration. Moreover, it should be noted that under hypoxic conditions, curcumin derivative compound **5** at a concentration of 4 µM and 5 µM also induced significant changes in cell cycle distribution. The results showed that after 24 h of treatment with compound **5** under hypoxic conditions, the number of cells in the proliferative G0/G1 phase was significantly decreased, and the number of cells in the S phase was slightly increased. This suggested the inhibitory effect on DNA replication.

#### 2.5.4. The Expression of Stress-Related Proteins after Treatment of 5637 Cells with PEGylated Curcumin

**Figure 14:**
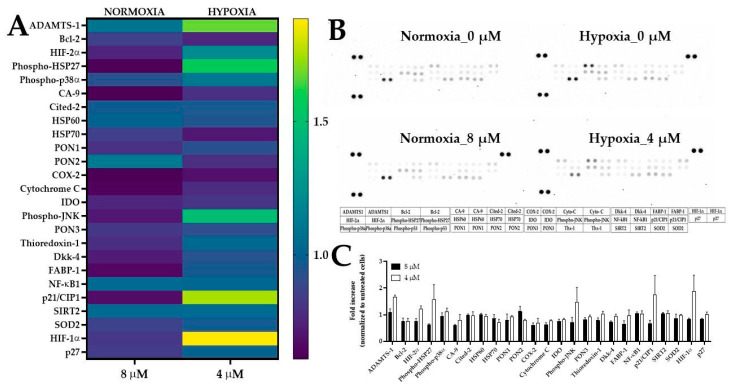
The effect of compound **5** on stress-related protein expression in 5637 cells under normoxic and hypoxic conditions. Panel (**A**) presents the expression of the proteins present as a heat map. Panel (**B**) shows the representative membrane images. Panel (**C**) presents the densitometric analysis expressed as the fold change in protein expression (relative to control means 1, a value greater than 1 means upregulation, and values less than 1 mean downregulation). Cells were treated with compound **5** at a concentration of 4 µM and 8 µM for 18 h. The densitometric analysis was performed using Image Studio Lite software. These data presented the mean values from two independent experiments. Chemiluminescent signals were detected by a charge-coupled device (CCD) camera-based digital imaging instrument (Thermofisher Scientific). Abbreviations: Bcl-2, B-cell lymphoma 2 protein; CA-9, carbonic anhydrase IX; Cited-2, Cbp/p300-interacting transactivator 2 protein; Cyto-C, cytochrome C; Dkk-4, Dickkopf WNT signaling pathway inhibitor 4; FABP-1, fatty acid-binding protein 1; HSP60, heat shock protein 60; HSP70, heat shock protein 70; IDO, indoleamine 2,3-dioxygenase; p27, cyclin-dependent kinase inhibitor 1B; PON1, human paraoxonase 1; PON2, human paraoxonase 2; PON3, human paraoxonase 3; SIRT2, sirtuin 2; SOD2, mitochondrial dismutase.

We used the Proteome Profiler Antibody Array to obtain further insights into the anticancer activity of compound **5** under normoxic and hypoxic conditions. For normoxia experiments, we used compound **5** at a concentration of 8 µM, while in hypoxia experiments, we used a lower concentration of 4 µM (Figure 14).

**Figure 15:**
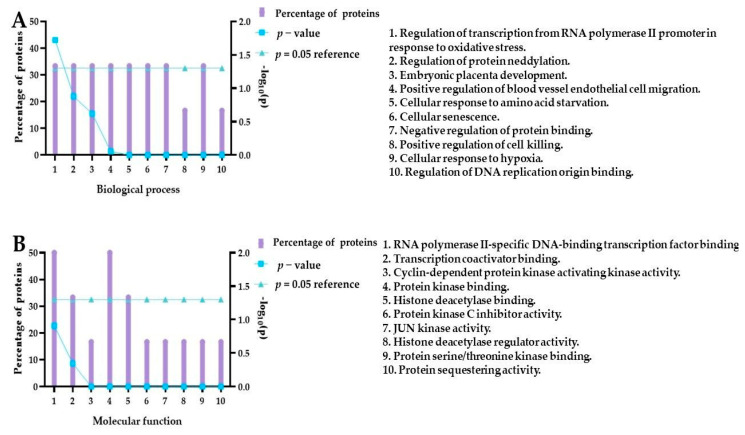
Enriched biological process and molecular function of proteins overexpressed after treatment with compound **5** under hypoxic conditions. The FunRich software further analyzed the six proteins (HIF-1α, HIF-2α, ADAMTS-1, phospho-JNK, p21, and phospho-HSP27) upregulated by compound **5** for pathway enrichment, e.g., biological process (panel **A**) and molecular function (panel **B**). The graph presents the top 10 enriched biological processes and molecular functions.

After incubation lasting 18 h, we analyzed the panel of 26 proteins and found that compound **5** under hypoxic conditions increased the expression of HIF-1α, HIF-2α, ADAMTS-1, phospho-JNK, p21, and phospho-HSP27 compared to control cells. Using a molecular function as a criterion, the role of the proteins whose expression levels have been increased is related to the kinase regulator activity, transcription factor activity, chaperone, protein serine/threonine kinase, and metallopeptidase activity. Furthermore, we performed analysis using FunRich software to identify biological processes related to these proteins, as shown in Figure 15.

The results indicated that the protein set was related, for example, to the regulation of transcription from RNA polymerase II, protein neddylation, cellular senescence, negative regulation of protein binding, positive regulation of cell killing, and cellular response to hypoxia. Interestingly, it was found that curcumin downregulated DNA topoisomerase II alpha, which is upregulated in many malignancies [56]. The activity of this enzyme is required for RNA polymerase II transcription on chromatin templates [57]. Our analysis also indicated that the neddylation process could be associated with our protein set. Neddylation is a posttranslational protein modification that conjugates a ubiquitin-like protein, developmental downregulation protein 8 (NEDD8), to substrate proteins z [58]. Although Cullin-RING E3 ubiquitin ligases (CRLs) are the most typical target proteins for neddylation [58], several other non-cullin substrates have been reported as potential targets for the canonical NEDD8 pathway involved in the control of the cell cycle, transcription, subcellular localization, nucleolar signaling, DNA damage response, neuronal maturation, and synaptic plasticity [59]. The following non-cullin substrates of neddylation have been reported: the tumor suppressor pVHL, EGFR, TGF-β type II receptor, HIF-1α/HIF-2α breast cancer-associated protein 3 (BCA3), APP intracellular domain (AICD), E2F-1, HECT-domain ubiquitin E3 ligase SMURF1, and RBR ubiquitin E3 ligase Parkin [60]. During the neddylation, NEDD8 binds to proteins, thereby promoting the CRLs’ control of numerous biological pathways and cellular processes by transferring ubiquitin to substrates and regulating the ubiquitination and degradation of approximately 20% of the proteins in mammalian cells [61]. Typical substrates of CRLs include proteins related to cell cycle regulation (e.g., Cyclin D/E, p21, and p27), apoptosis (e.g., BIM, NOXA, and BIK), and signal transduction pathways (e.g., HIF-1α, REDD1, and β-catenin) [60]. The balance between the neddylation and deneddylation is crucial to maintain the proper function of CRLs by controlling adaptor subunit exchange and preventing autoubiquitination of the CRL complexes in the absence of substrates. Previous studies have demonstrated that the remodeling of CRLs is initiated by the cleavage of NEDD8 from CRLs, catalyzed by the deneddylase COP9 signalosome (CSN). Noteworthily, it was demonstrated that curcumin inhibits CSN-associated kinases to induce the proteasome-dependent degradation of the inhibitor of differentiation/DNA binding 1 (Id1) and inhibitor of differentiation/DNA binding 3 (Id3), which are known regulators of tumor angiogenesis stimulated by CSN-directed c-Jun signaling [62]. The inhibition of the COP9 signalosome (CSN)-associated kinases, casein kinase 2 (CK2), and protein kinase D (PKD), by curcumin, causes stabilization of the tumor suppressor p53 [63]. Fullbeck and co-workers demonstrated that curcumin blocks the CSN-directed c-Jun signaling pathway, which results in diminished c-Jun steady-state levels in HeLa cells [64]. Li et al. demonstrated that water-soluble polyethylene glycol-conjugated curcumin inhibits pancreatic cancer cell proliferation by activating Jab1 (Jun activation domain-binding protein 1)/CSN5 [37]. It was also reported that the 4-arylidene curcumin analog, T83, might act as a Jab1 inhibitor. T83 significantly inhibited cell proliferation in nasopharyngeal cancer cells, blocked cells at the G2/M cell cycle, and sensitized the cells to radiotherapy [65]. ab1/CSN5 is a component of the CSN involved in the degradation of the tumor suppressor p53, the cell cycle inhibitors p27/Kip1 (cyclin-dependent kinase inhibitor 1B), and p21/Cip1 (cyclin-dependent kinase interacting protein 1), which are actively involved in suppressing cancer cell proliferation [37]. Thus, as Jab1 is overexpressed in many cancers, including bladder cancer [66], the inhibition of CNS and dysregulation of the neddylation and deneddylation cycles by curcumin and its derivatives are important in designing therapeutic approaches.

The protein p21 (p21^WAF1/Cip1^) belongs to the Cip/Kip family and acts as a cyclin-dependent kinase inhibitor [67]. Aljabery and co-workers showed that p21 expression in p53-negative tumors was associated with a significant decrease in cell proliferation in patients with muscle-invasive bladder cancer (MIBC) treated with cystectomy [68]. Furthermore, patients with a low p21 expression had shorter recurrence-free survival and overall survival rates [69]. Presented results showed that compound **5** at hypoxic conditions increased the p21 expression in 5637 cells. The p21 protein is well-known as a regulator of G1/S; however, it should be noted that p21 affects multiple cell cycle phases, depending on the cellular context [70]. Results from the proteome profiler are in line with cell cycle distribution analysis. At the same concentration, we observed significant changes in cell cycle distribution in phases G1/G0 and S that might be related to p21 overexpression. Noteworthily, we observed the elevated expression of HIF-1α. As reported by Koshiji et al., HIF-1α is important for hypoxia-induced growth arrest and the activation of p21 [71].

Compound **5** under hypoxic conditions also increased the phospho-HSP27 level in 5637 cells. The role of HSP27 in cancer development and resistance to treatment is still controversial. It was reported that the gemcitabine-induced phosphorylation of HSP27 via the p38 mitogen-activated protein kinase–MAP kinase-activated protein kinase 2 (p38 MAPK–MAPKAPK-2) pathway decreased pancreatic cancer cell proliferation [72]. Liang and co-workers found that the downregulation of HSP27 in colorectal cancer cells affects curcumin activity [73]. The silencing of HSP27 changed the anticancer effect of curcumin by reducing the expression of proteins involved in apoptosis (p-Akt, Akt, Bcl-2, and p-Bad), increasing the level of antiapoptotic proteins (Bad and c-PARP), decreasing ROS generation, and autophagy induction. Thus, similar behavior can be related to curcumin derivatives, and the elevated p-HSP27 protein might play an important role in inducing cancer cell death.

A disintegrin and metalloprotease with thrombospondin motifs (ADAMTS) are metalloproteases secreted and bound to the extracellular matrix (ECM) through their C-terminal regions [74]. Previous studies identified 19 ADAMTS proteins in humans [75]. They share homology in the catalytic proteinase and disintegrin domains; however, they differ in the number of thrombospondin-like motifs and other carboxyl-terminal domains, which determines their interaction with the ECM [76]. Taking into account their functions, we can distinguish those proteins that participate in the polymerization of ECM proteins (ADAMTS2, ADAMTS3, ADAMTS10, and ADAMTS17), while others catalyze proteoglycan degradation (ADAMTS, ADAMTS4, ADAMTS5, ADAMTS7, ADAMTS8, ADAMTS9, ADAMTS12, ADAMTS15, ADAMTS16, ADAMTS18, and ADAMTS20) [74]. The ADAMTS-1 protein has been the most characterized in cancer, while its role is still controversial as the literature data indicates both suppressing [77] and tumor-promoting potential [78]. It was reported that ADAMTS-1 inhibits proliferation, polarization, and migration in ovarian cancer cell lines [79]. Noteworthily, the overexpression of ADAMTS-1 was linked with decreasing vascular endothelial growth factor (VEGF) expression and the proliferation of the lung cancer A549 cell line [80]. Indeed, the antiangiogenic activity is one of the well-known and described functions of ADAMTS-1. As demonstrated by Obika et al., ADAMTS-1 inhibited endothelial cell migration and proliferation and induced apoptosis [81]. Furthermore, the authors found that ADAMTS-1 suppressed vessel formation in vivo using a mice xenograft model established for human fibrosarcoma (HT1080), human prostate (DU145) carcinoma cell line, and Chinese hamster ovary (CHO-K1) cell line [81]. These inhibitory effects of ADAMTS-1 might be achieved independently of its proteolytic activity, while the authors used the full-length ADAMTS-1 and catalytic domain-deleted ADAMTS-1 for cell transfection [81]. Moreover, it was demonstrated that hypoxia upregulated ADAMTS-1 expression, and the transcriptional activation of ADAMTS-1 was mediated by HIF-1, which binds to hypoxia response elements (HREs) in the promoter region of the ADAMTS1 gene [82]. We observed the increased level of ADAMTS-1 in cells treated with compound **5** under hypoxic, not normoxic, conditions. Hypoxia can also increase VEGF expression [83], so the elevated ADAMTS-1 can act as a compensatory mechanism. However, the role of ADAMTS-1 in curcumin activity and its derivatives in bladder cancer cells has not been studied. Therefore, our results indicate that the relationship between ADAMTS-1 and curcumin derivatives under hypoxic conditions is an interesting starting point for further studies.

It is widely known that curcumin possesses anti-inflammatory activity by significantly reducing the production of proinflammatory mediators, including cyclooxygenase 2 (COX-2) and prostaglandin E2, and through the inhibition of the NF-κB pathway [84]. Interestingly, for both concentrations, we observed a decrease in COX-2 expression compared to control cells, which was associated with the unchanged level of NF-κB1 protein. A high expression of COX-2 is associated with cell proliferation, migration, and invasion in cancer cells [85,86]. A recent report showed that 5637 cells expressed high levels of COX-2 mRNA [87]; thus, affecting the COX-2 expression might suppress tumor growth and play a crucial role in chemoprevention.

### 2.6. Computational Modeling to Predict ADMET Properties

**Figure 16:**
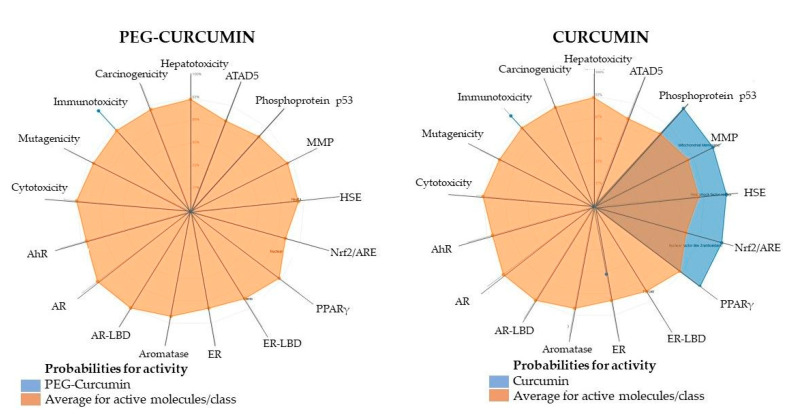
Toxicity radars for compound **5** and curcumin. The orange dots/lines represent the predicted probabilities of the input structure for corresponding ProTox-II models. The predicted toxicity result is created by comparing the average confidence score of the active compounds in the training set of each model, to that of the input compounds (compound **5** and curcumin). Abbreviations: AhR, aryl hydrocarbon receptor; AR, androgen receptor; AR-LBD, androgen receptor ligand-binding domain; ATAD5, ATPase family AAA domain-containing 5; ER, estrogen receptor; ER-LBD, estrogen receptor ligand-binding domain; HSE, heat shock factor response element; MMP, mitochondrial membrane potential; Nrf2/ARE, nuclear erythroid 2-related factor 2/antioxidant response element; PPARγ, peroxisome proliferator-activated receptor.

Since the publication in 1996 by Lipinski et al., the “rule of five” has become widely accepted in the pharmaceutical community [88]. The proposed rule states that most currently used drugs have a molecular weight < 500, logP < 5, the amount of hydrogen bond donors < 5, and acceptors < 10. It also notes that compounds that function as substrates for biological transporters are exceptions to the rule. There are now many tools to estimate ADMET (absorption, distribution, metabolism, excretion, and toxicity) properties of newly developed drug candidates [89,90,91]. Accordingly, we have elected to use two: ProTox-II [92] and admetSAR [89]. We analyzed two PEGylated and two non-PEGylated curcuminoids, four compounds in total. In each tested pair, one was a difluoroborinate complex. The results are presented in Table 2. Briefly, all tested compounds had a logP under the optimal 5 value and a polar surface area less than 140Å [93]. Interestingly, curcumin is the only compound that should not cross the blood–brain barrier, likely due to its low logP. In the test for carcinogenicity, the curcumin difluoroborinate complex was the only compound to show a response. All of the tested compounds show similar Caco-2 permeability, but as expected in each case, PEGylation increases this value compared to the parent variant. All four compounds were nearly equivalent substrates for P-glycoprotein, but both PEGylated curcuminoids also act as inhibitors, slowing down their elimination from the cell. This may explain the slightly lower IC_50_ values for PEGylated curcuminoids after 48 h of incubation versus 24 h. Following the prediction study, it is worth noting that compound **5** demonstrated significantly lower acute oral toxicity compared to its difluoroborinate complex and curcumin. In Figure 16, the toxicity radar plots are presented. The average confidence score of the active agents in the training set of each model to that of compound **5** and curcumin was compared.

### 2.7. Study Limitations and Future Directions

Our work should be considered preliminary research that sets the direction for further mechanistic studies by showing the cellular targets and related signaling pathways. Moreover, additional solubility and stability studies are necessary to verify the advantages of the proposed chemical modifications. Therefore, considering this critical aspect, further research on the chemical stability and activity of curcumin derivatives in physiologically relevant conditions should be performed to better assess their potential in animal-based preclinical studies. Noteworthily, our initial findings are the starting point for further experiments, including the design of delivery systems and nanoformulations to develop an innovative approach to eliminate cancer cells and improve our understanding of the anticancer mechanism of curcumin derivatives.

## 3. Materials and Methods

### 3.1. Materials

All substrates and solvents used in synthesis were purchased from either Alfa Aesar, TCI, or Merck. MALDI-TOF spectra were registered using an UltrafleXtreme (Bruker Daltonics, Bremen, Germany). ESI-MS spectra were registered using an Agilent Technologies 6410B Triple Quadrupole LC/MS System with an Agilent 1200/1290 HPLC (Agilent Technologies, Santa Clara, CA, USA). NMR spectra were recorded at 298 K either on a Bruker Avance III 500 spectrometer (Bruker Daltonics, Bremen, Germany) using PABBO BBF/1Hand19F 5 mm probe or an Agilent DD2 800 spectrometer (Agilent Technologies, Santa Clara, CA, USA) equipped with a 5 mm ^1^H(^13^C/^15^N) probe head. UV–vis spectra were recorded on a UV–vis Jasco V-770 spectrophotometer (JASCO, Tokyo, Japan). The UV–vis spectra in DMSO were recorded using a Tecan Infinite M PLEX (Tecan, Switzerland). HPLC determined compound purity on an Agilent 1260 Infinity II chromatograph.

Reagents used for in vitro experiments, such as Dulbecco’s modified Eagle’s medium (DMEM), fetal bovine serum (FBS), penicillin–streptomycin-L-glutamine solution, phosphate-buffered saline (PBS), trypsin-EDTA, dimethyl sulfoxide (DMSO), 3-(4, 5-dimethylthiazol-2-yl)-2,5-diphenyltetrazolium bromide (MTT), and propidium iodide (PI), were obtained from Sigma-Aldrich (St. Louis, MO, USA). Roswell Park Memorial Institute 1640 (RPMI) medium, Eagle’s Minimum Essential Medium (EMEM), and Pierce BCA protein assay Kit were obtained from Thermofisher Scientific (Waltham, MA, USA). Aprotinin, leupeptin hemisulfate, and pepstatin A were purchased from Tocris (Bio-Techne, Bristol, UK). The DMSO for dissolving formazan crystals was obtained from Avantor Performance Materials (Gliwice, Poland).

### 3.2. Synthesis

#### 3.2.1. PEGylated Aldehyde Synthesis—General Procedure

Aromatic aldehyde PEGylation was performed using the standard nucleophilic substitution method. To a solution of the corresponding aldehyde (vanillin, isovanillin, or syringaldehyde) in DMF, 1.1 equivalent of Br(CH_2_CH_2_O)_3_CH_3_ and 1 equivalent of K_2_CO_3_ was added. The reaction was then heated to 80 °C and stirred for 24 h. After that, the reaction was quenched with distilled water, and the product was extracted with ethyl acetate and purified using column chromatography.

*3-methoxy-4-(2-(2-(2-methoxyethoxy)ethoxy)ethoxy)benzaldehyde* (**1**) ESI [M+H]^+^ *m/z* 299.2,

^1^H NMR (500 MHz, DMSO-*d*_6_) δ 9.84 (s, 1H), 7.53 (dd, *J* = 8.2, 1.9 Hz, 1H), 7.39 (d, *J* = 1.8 Hz, 1H), 7.18 (d, *J* = 8.3 Hz, 1H), 4.24–4.16 (m, 2H), 3.83 (s, 3H), 3.78 (dd, *J* = 5.2, 4.0 Hz, 2H), 3.59 (dd, *J* = 5.9, 3.5 Hz, 2H), 3.55–3.52 (m, 2H), 3.52–3.49 (m, 2H), 3.42 (dd, *J* = 5.7, 3.8 Hz, 2H), 3.23 (s, 3H).

^13^C NMR (126 MHz, DMSO-*d*_6_) δ 191.36, 153.43, 149.22, 129.70, 125.96, 112.17, 109.68, 71.26, 69.97, 69.78, 69.59, 68.68, 68.12, 58.01, 55.49.

Column chromatography—ethyl acetate. Rf(ethyl acetate): 0.52, yield 79.2% brown viscous liquid

*4-methoxy-3-{2-[2-(2-methoxyethoxy)ethoxy]ethoxy}benzaldehyde* (**2**) ESI [M+H]^+^ *m/z* 299.2,

^1^H NMR (500 MHz, DMSO-*d*_6_) δ 9.83 (s, 1H), 7.56 (dd, *J* = 8.2, 1.9 Hz, 1H), 7.41 (d, *J* = 1.8 Hz, 1H), 7.18 (d, *J* = 8.3 Hz, 1H), 4.17–4.13 (m, 2H), 3.87 (s, 3H), 3.76 (dd, *J* = 5.2, 4.0 Hz, 2H), 3.59 (dd, *J* = 5.9, 3.5 Hz, 2H), 3.55–3.52 (m, 2H), 3.52–3.50 (m, 2H), 3.42 (dd, *J* = 5.7, 3.8 Hz, 2H), 3.23 (s, 3H).

^13^C NMR (126 MHz, DMSO-*d*_6_) δ 191.32, 154.32, 148.32, 129.59, 126.00, 111.48, 110.89, 71.24, 69.93, 69.77, 69.58, 68.79, 67.89, 58.00, 55.85.

Column chromatography—ethyl acetate. Rf(ethyl acetate): 0.43, yield 83.6% brown viscous liquid.

*3,5-dimethoxy-4-{2-[2-(2-methoxyethoxy)ethoxy]ethoxy}benzaldehyde* (**3**)

^1^H NMR (800 MHz, CDCl_3_) δ 9.84 (s, 1H), 7.10 (s, 2H), 4.24–4.21 (m, 2H), 3.89 (s, 6H), 3.81–3.79 (m, 2H), 3.71–3.69 (m, 2H), 3.64 (dd, *J* = 5.8, 4.1 Hz, 2H), 3.63–3.61 (m, 2H), 3.53–3.51 (m, 2H), 3.35 (s, 3H).

^13^C NMR (201 MHz, CDCl_3_) δ 191.17, 153.89, 142.87, 131.86, 106.80, 72.48, 72.04, 70.76, 70.74, 70.64, 70.55, 59.11, 56.36.

Column chromatography—ethyl acetate. R_f_(ethyl acetate): 0.57, yield 81.3% brown viscous liquid.

#### 3.2.2. Curcuminoids Synthesis

General Procedure: Difluoroborinate complexes were synthesized by the condensation of acetylacetone difluoroborinate complex with an appropriate aldehyde (2.1 equivalent) in toluene. Tributyl borate (2 equivalent) was added as a dehydrating agent, whereas *n*-butylamine (0.2 equivalent) in toluene was added dropwise to the reaction mixture over 15 min. The reaction mixture was heated at 65 °C for 24 h under nitrogen. The upper layer of the reaction mixture was then decanted, whereas the dark red amorphous layer at the bottom of the reaction flask was dried under vacuum, and the solid residue was purified using flash column chromatography.

Subsequently, free curcumins were released from the difluoroborinate complexes. The difluoroborinate complexes were placed together with disodium oxalate in a methanol:water (4:1) mixture under sealed microwave conditions at 140 °C for 20 min in an Anton Paar Monowave 400 microwave reactor. After the microwave reaction, the solvents were evaporated under a vacuum and the solid residue was purified using flash column chromatography.

*(1E,3Z,6E)-1,7-bis(3-methoxy-4-{2-[2-(2-methoxyethoxy)ethoxy]ethoxy}phenyl)-5-oxohepta-1,3,6-trien-3-yl difluoroborinate* (**4**) MALDI-TOF [M-F]^+^ *m/z* 689.3410

^1^H NMR (800 MHz, CDCl_3_) δ 7.91 (d, *J* = 15.4 Hz, 2H), 7.15 (dd, *J* = 8.3, 1.6 Hz, 2H), 7.07 (d, *J* = 1.6 Hz, 2H), 6.90 (d, *J* = 8.4 Hz, 2H), 6.56 (d, *J* = 15.4 Hz, 2H), 6.04 (s, 1H), 4.24–4.21 (m, 4H), 3.92–3.88 (m, 10H), 3.74 (dd, *J* = 5.7, 3.9 Hz, 4H), 3.68 (dd, *J* = 5.7, 3.9 Hz, 4H), 3.66 – 3.64 (m, 4H), 3.56–3.54 (m, 4H), 3.38 (s, 6H).

^13^C NMR (201 MHz, CDCl_3_) δ 179.30, 152.06, 149.74, 147.00, 127.44, 124.42, 118.36, 112.89, 110.94, 101.76, 71.94, 70.90, 70.65, 70.56, 69.43, 68.46, 59.03, 55.99.

R_f_(dichloromethane:acetone 4:1) 0.33 yield 80.3%

*(1E,4Z,6E)-5-hydroxy-1,7-bis(3-methoxy-4-{2-[2-(2-methoxyethoxy)ethoxy]ethoxy}phenyl)-hepta-1,4,6-trien-3-one* (**5**) MALDI-TOF [M+H]^+^ *m/z* 661.3199

^1^H NMR (800 MHz, CDCl_3_) δ 16.03 (s, 1H), 7.59 (d, *J* = 15.7 Hz, 2H), 7.11 (dd, *J* = 8.4, 1.9 Hz, 2H), 7.07 (d, *J* = 2.0 Hz, 2H), 6.92 (d, *J* = 8.3 Hz, 2H), 6.49 (d, *J* = 15.7 Hz, 2H), 5.82 (s, 1H), 4.23–4.21 (m, 4H), 3.92–3.88 (m, 10H), 3.74 (dd, *J* = 5.7, 3.9 Hz, 4H), 3.69–3.67 (m, 4H), 3.66–3.64 (m, 4H), 3.57–3.53 (m, 4H), 3.38 (s, 6H).

^13^C NMR (201 MHz, CDCl_3_) δ 183.23, 150.41, 149.69, 140.36, 128.42, 122.44, 122.14, 113.20, 110.46, 101.27, 71.94, 70.88, 70.65, 70.56, 69.52, 68.45, 59.03, 55.98.

R_f_(dichloromethane:acetone 4:1) 0.42 yield 25.6%

*(1E,3Z,6E)-1,7-bis(4-methoxy-3-{2-[2-(2-methoxyethoxy)ethoxy]ethoxy}phenyl)-5-oxohepta-1,3,6-trien-3-yl difluoroborinate* (**6**) MALDI-TOF [M-F]^-^ *m/z* 689.3494

^1^H NMR (800 MHz, CDCl_3_) δ 7.90 (d, *J* = 15.4 Hz, 2H), 7.18–7.14 (m, 4H), 6.85 (d, *J* = 8.3 Hz, 2H), 6.54 (d, *J* = 15.4 Hz, 2H), 6.03 (s, 1H), 4.23 – 4.20 (m, 4H), 3.90 (dd, *J* = 5.5, 4.5 Hz, 4H), 3.89 (s, 6H), 3.75–3.74 (m, 4H), 3.69–3.67 (m, 4H), 3.65–3.64 (m, 4H), 3.55–3.53 (m, 4H), 3.36 (s, 6H).

^13^C NMR (201 MHz, CDCl_3_) δ 179.41, 153.30, 148.83, 147.08, 127.27, 125.26, 118.41, 112.84, 111.68, 101.89, 72.04, 70.96, 70.76, 70.65, 69.75, 68.80, 59.12, 56.12.

R_f_(dichloromethane:acetone 4:1) 0.43 yield 78.2%

*(1E,4Z,6E)-5-hydroxy-1,7-bis(4-methoxy-3-{2-[2-(2-methoxyethoxy)ethoxy]ethoxy}phenyl)-hepta-1,4,6-trien-3-one* (**7**) MALDI-TOF [M-H]^-^ *m/z* 659.2984

^1^H NMR (800 MHz, CDCl_3_) δ 16.04 (s, 1H), 7.59 (d, *J* = 15.7 Hz, 2H), 7.16–7.13 (m, 4H), 6.87 (d, *J* = 8.2 Hz, 2H), 6.49 (d, *J* = 15.7 Hz, 2H), 5.81 (s, 1H), 4.25–4.22 (m, 4H), 3.93–3.90 (m, 4H), 3.89 (s, 6H), 3.77–3.75 (m, 4H), 3.70–3.68 (m, 4H), 3.67–3.65 (m, 4H), 3.56–3.55 (m, 4H), 3.38 (s, 6H).

^13^C NMR (201 MHz, CDCl_3_) δ 183.25, 151.62, 148.54, 140.33, 128.04, 123.14, 122.05, 112.40, 111.61, 101.35, 71.94, 70.87, 70.66, 70.56, 69.68, 68.66, 59.02, 55.97.

R_f_(dichloromethane:acetone 4:1) 0.48 yield 27.8%

*(1E,3Z,6E)-1,7-bis(3,5-dimethoxy-4-{2-[2-(2-methoxyethoxy)ethoxy]ethoxy}phenyl)-5-oxohepta-1,3,6-trien-3-yl difluoroborinate* (**8**) MALDI-TOF [M-F]^+^ *m/z* 749.3601

^1^H NMR (800 MHz, CDCl_3_) δ 7.89 (d, *J* = 15.4 Hz, 2H), 6.80 (s, 4H), 6.62 (d, *J* = 15.5 Hz, 2H), 6.14 (s, 1H), 4.21–4.19 (m, 4H), 3.87 (s, 6H), 3.80–3.78 (m, 4H), 3.71 (dd, *J* = 5.8, 3.9 Hz, 4H), 3.66 (dd, *J* = 5.8, 3.9 Hz, 4H), 3.64 (dd, *J* = 5.6, 3.8 Hz, 4H), 3.54 (dd, *J* = 5.6, 3.8 Hz, 4H), 3.37 (s, 6H).

^13^C NMR (201 MHz, CDCl_3_) δ 179.76, 153.74, 147.38, 140.80, 129.72, 119.94, 106.57, 102.23, 72.56, 72.02, 70.74, 70.72, 70.63, 70.56, 59.14, 56.35.

R_f_(dichloromethane:acetone 4:1) 0.33 yield 71.3%

Compound **9,** which would have been the non-fluoroborinated version of compound **8,** could not be isolated due to decomposition.

*(1E,3Z,6E)-1,7-bis(3-methoxy-4-hydroxyphenyl)-5-oxohepta-1,3,6-trien-3-yl difluoroborinate* (**10**) ESI [M-F]^+^ *m/z* 397.1

^1^H NMR (800 MHz DMSO-*d*_6_) δ 10.06 (s, 2H), 7.89 (d, *J* = 15.5 Hz, 2H), 7.45 (d, *J* = 1.9 Hz, 2H), 7.32 (dd, *J* = 8.3, 1.9 Hz, 2H), 6.99 (d, *J* = 15.6 Hz, 2H), 6.85 (d, *J* = 8.2 Hz, 2H), 6.43 (s, 1H), 3.83 (s, 6H).

^13^C NMR (201 MHz, DMSO-*d_6_*) δ 178.69, 151.31, 148.14, 146.92, 125.95, 125.21, 117.84, 115.92, 112.40, 101.05, 55.75.

R_f_(dichloromethane:acetone 7:1) 0.72 yield 88.4%

*(1E,4Z,6E)-5-hydroxy-1,7-bis (3-methoxy-4-hydroxyphenyl)-hepta-1,4,6-trien-3-one* (**11**)

ESI [M+H]^+^ *m/z* 369.1

^1^H NMR (800 MHz, DMSO-*d*_6_) δ 16.39 (s, 1H), 9.64 (s, 2H), 7.54 (d, *J* = 15.8 Hz, 2H), 7.32 (d, *J* = 1.9 Hz, 2H), 7.15 (dd, *J* = 8.3, 1.9 Hz, 2H), 6.82 (d, *J* = 8.1 Hz, 2H), 6.75 (d, *J* = 15.8 Hz, 2H), 6.06 (s, 1H), 3.84 (s, 6H).

^13^C NMR (201 MHz, DMSO-*d*_6_) δ 183.18, 149.33, 147.96, 140.67, 126.31, 123.10, 121.07, 115.68, 111.33, 100.78, 55.68.

R_f_(dichloromethane:acetone 7:1) 0.78 yield 57.8%

(1E,3Z,6E)-1,7-bis(4-methoxy-3-hydroxyphenyl)-5-oxohepta-1,3,6-trien-3-yl difluoroborinate (**12**) ESI [M-F]^+^ *m/z* 397.1

^1^H NMR (800 MHz, DMSO-*d*_6_) δ 9.37 (s, 2H), 7.88 (d, *J* = 15.6 Hz, 2H), 7.33 (dd, *J* = 8.5, 2.0 Hz, 2H), 7.27 (d, *J* = 2.0 Hz, 2H), 7.05 (d, *J* = 8.5 Hz, 2H), 6.92 (d, *J* = 15.6 Hz, 2H), 6.56 (s, 1H), 3.86 (s, 6H).

^13^C NMR (201 MHz, DMSO-*d*_6_) δ 178.94, 151.75, 146.91, 146.72, 127.17, 123.71, 118.52, 114.79, 112.13, 101.52, 55.78.

R_f_(dichloromethane:acetone 7:1) 0.68 yield 94.3%

(1E,4Z,6E)-5-hydroxy-1,7-bis(4-methoxy-3-hydroxyphenyl)-hepta-1,4,6-trien-3-one (**13**) ESI [M+H]^+^ *m/z* 369.1

^1^H NMR (800 MHz, DMSO-*d*_6_) δ 16.29 (s, 1H), 9.21 (s, 2H), 7.50 (d, *J* = 15.8 Hz, 2H), 7.24–7.08 (m, 4H), 6.98 (d, *J* = 8.1 Hz, 2H), 6.65 (d, *J* = 15.8 Hz, 2H), 6.12 (s, 1H), 3.82 (s, 6H).

^13^C NMR (201 MHz, DMSO-*d*_6_) δ 183.08, 150.03, 146.74, 140.41, 127.62, 121.64, 121.40, 114.08, 112.05, 101.12, 55.65.

R_f_(dichloromethane:acetone 7:1) 0.75 yield 62.3%.

#### 3.2.3. HPLC Purity

HPLC analysis was performed to assess the purity of the new PEGylated curcumin using high-performance liquid chromatography coupled, in series, to a diode array detector (DAD) and evaporative light scattering detector (ELSD). To analyze the purity, the high-concentration solution of tested compounds (0.2 mg/mL) was used. The sample was prepared by the dissolution of the compound in acetonitrile. The detection wavelength was adjusted for each tested compound at their absorption maxima. The elution of compounds was achieved on the reversed-phase column used as the stationary phase (C-18 (2) 100 Å Luna^®^, 150 × 4.6 mm ID, 5 µm, Phenomenex, Torrance, CA, USA), and gradient solvent systems of 0.4% acetic acid (phase A) and acetonitrile (phase B) were used as the mobile phase with a flow rate of 1.0 mL/min. A sample volume of 10 µL was injected into the column. All chromatograms showed the single main peak between 11.21 min and 13.53 min, with purity ranging from 95.1% to 99.4%. Detailed data are presented in the Supplementary Materials (Tables S1 and S2).

### 3.3. Cell Culture Conditions

Human urinary grade II carcinoma cell line (5637) and human urinary squamous cell carcinoma (SCaBER) were purchased from ATCC (Manassas, VA, USA). The MRC-5 cell line (normal human lung fibroblasts) was purchased from the European Collection of Authenticated Cell Cultures (ECACC) distributed by Sigma-Aldrich. The 5637 cells were grown in RPMI, SCaBER cells were cultured in EMEM, and MRC-5 in DMEM. All media were supplemented with 10% (*v/v*) FBS, 2 mM L-glutamine, 100 U/mL penicillin, and 10 mg/mL streptomycin in a humidified atmosphere of 5% CO_2_ at 37 °C.

For the hypoxia experiments, cells were cultured at 1% O_2_ (5% CO_2_/94% N_2_; 37 °C) using a hypoxia station (Whitley H35 hypoxystation; Don Whitley, Bingley, UK). All media used in these experiments were incubated under hypoxic conditions before being added to the cells.

Stock solutions of each compound were prepared by dissolving in DMSO and stored in the dark at −20 °C. The final concentration of DMSO in the experiments did not exceed 0.1% in the cell culture medium.

### 3.4. In Vitro Anticancer Activity

5637 and SCaBER cells were seeded at 15 × 10^3^ cells/well, while MRC-5 was at a density of 20 × 10^3^ cells/well and incubated overnight. In the case of the hypoxia experiments, cells were transferred to a hypoxia chamber and incubated for 24 h. Then, cells were treated with tested compounds at a concentration of 0.3 µM, 0.6 µM, 1.2 µM, 2.5 µM, 5 µM, and 10 µM for curcumin and isocurcumin derivatives **3**, **4**, **6**, **10**, and **12**. Compound **13** was tested at a concentration of 0.6 µM 1.2 µM, 2.5 µM, 5 µM, 10 µM, and 20 µM, while curcumin was used at a concentration of 3 µM, 6 µM, 12 µM, 25 µM, 50 µM, and 100 µM. The DMSO was used as a control, and its concentration in the cell culture medium did not exceed 0.1%. The cytotoxic activity of tested compounds was measured after 24 h and 48 h using the MTT assay. After incubation lasting 24 h and 48 h, the cells were washed twice with PBS, and 170 µL of MTT (final concentration of 0.59 mg/mL in cell culture medium) was added to each well. Cells were incubated for 1.5 h under standard cell culture conditions. Then, DMSO was added to dissolve the formazan crystals. The absorbance was measured at a wavelength of 570 nm with a plate reader (Biotek Instruments, Elx-800, Winooski, VT, USA). Cell viability was calculated as a percentage of the control. All experiments were repeated at least two times (two independent experiments performed in hexaplicate). The IC_50_ values were determined using GraphPad 8.0 software (GraphPad Software, Inc., La Jolla, CA, USA).

### 3.5. Cellular Uptake

The 5637 cells (0.1 × 10^6^ cells/well) were seeded in 24-well plates and incubated overnight under standard cell culture conditions. Then, the curcumin and compound **5** were added at a concentration of 10 µM. DMSO was used as a control, and its concentration in the cell culture medium did not exceed 0.1%. After incubation lasting 0.5 h, 2 h, 4 h, and 8 h, cells were washed twice with PBS, trypsinized, centrifuged, and washed twice with PBS. The samples were analyzed using BD FACSLyric™ (Becton Dickinson, Franklin Lakes, NJ, USA). The results were analyzed using FlowJo software version 10.8.1 (Becton Dickinson, Ashland, OR, USA).

### 3.6. Cell Cycle Analysis

The 5637 cells (0.4 × 10^6^ cells/well) were seeded in 6-well plates and incubated overnight under standard cell culture conditions or in a hypoxia workstation. Then, the tested compound was added at a concentration of 4 µM, 5 µM, and 6 µM (for hypoxic conditions), and 6 µM, 8 µM, and 10 µM (for normoxic conditions), and incubated for 24 h. DMSO was used as a control, and its concentration in the cell culture medium did not exceed 0.1%. After incubation, cells were washed twice with PBS, trypsinized, and washed again with PBS. Cells were fixed with 70% ethanol at 4 °C. After fixation, the cells were washed twice with PBS and treated with RNAse (100 µg/mL) and PI (50 µg/mL) for 30 min at 37 °C in the dark. The samples were analyzed using BD FACSLyric™ (Becton Dickinson, Franklin Lakes, NJ, USA). Finally, cell cycle distribution was analyzed using flow cytometry. The percentages of cells in the G1, S, and G2/M phases were calculated using FlowJo software version 10.8.1 (Becton Dickinson, Ashland, OR, USA).

### 3.7. Proteome Profiler Array

The 5637 cells were seeded in a Petri dish (Ø 35 mm) at a density of 0.4 × 10^6^ cells/dish and incubated overnight. Cells were treated with compound **5** at a concentration of 4 µM (hypoxia) and 8 µM (normoxia) for 18 h. DMSO was used as a control, and its concentration in the cell culture medium did not exceed 0.1%. After incubation, cells were lysed in a lysis buffer containing pepstatin A, leupeptin, and aprotinin (each at a concentration of 10 µg/mL). Total protein was determined using a Pierce BCA protein assay Kit and stored at −80 °C until future use. A total of 300 µg of protein was then incubated with a human Proteome Profiler Human Cell Stress Array (Bio-Techne, Minneapolis, MN, USA) according to the manufacturer’s instructions. The membranes were visualized using the I-Bright Imaging System (Thermofisher Scientific, Waltham, MA, USA). The spot densitometric analysis was performed using Image Studio Lite (version 5.2) software (LI-COR Biosciences, Lincoln, NE, USA).

### 3.8. Functional Enrichment Analysis

FunRich is an open-access analytical tool for the functional enrichment and interaction network analysis of genes and proteins [94]. The FunRich analysis is based on a backend database with human-specific collated genomic and proteomic datasets of more than 1.5 million annotations, which were regularly updated [95]. FunRich version 3.1.3 (www.funrich.org accessed on 1.11.2022) and the UniProt database were used for the analysis. The ten biological pathways most important for the upregulated proteins and the ten molecular functions were presented.

### 3.9. ADMET Properties Analysis Using the Computational Method

It is common practice in drug discovery research to predict the ADME (absorption, distribution, metabolism, excretion, and toxicity) toxicological properties using computational methods. Several computational tools are freely available for scientists to use to predict and analyze the ADMET properties. Our study used two tools: ProTox-II (http://tox.charite.de/protox_II, accessed on 26 October 2022) and the admetSAR server (http://lmmd.ecust.edu.cn:8000/, accessed on 26 October 2022). ADMET parameter analysis was performed for compounds **4**, **5**, **10**, and the reference compound, curcumin.

### 3.10. Statistical Analysis

The statistical analysis was performed using GraphPad Prism^®^8 (GraphPad Software, Inc., La Jolla, CA, USA). One-way ANOVA with post hoc Dunnett’s test was used to determine the significance; *p* < 0.05 was considered significant.

## 4. Conclusions

This work presents a series of BF_2_ and PEG-modified curcumin and isocurcumin derivatives, which were subjected to physicochemical and biological studies. The cytotoxicity tests performed on the series of compounds under normoxic and hypoxic conditions allowed us to select the most active molecule. It was demonstrated that the cytotoxic activities of curcumin and isocurcumin could be increased by inserting the BF_2_ moiety into their chemical structure. However, this modification also reveals a severe drawback as molecules enriched in the BF_2_ motif lost their selectivity toward cell lines and exerted the strongest activity toward the noncancerous cell line. Interestingly, incorporating the PEG moiety into the molecule decreased the cytotoxic effect against normal cells. It was found that the observed effect against cells relies on the position of the PEG insertion within the phenolic motif of curcumin and isocurcumin and that the substitution of position 4 is favorable in this regard for curcumin derivatives with an unmodified diketo moiety. Curcumin derivative **5** was selected for further research due to the lack of cytotoxic activity against the MRC-5 line and higher activity under hypoxic conditions. The cellular uptake studies performed for compound **5** and curcumin indicated that short-chain PEG modifications did not improve the uptake by cancer cells compared to curcumin. Furthermore, we also noted that a lack of serum proteins is affected by cellular uptake, which can be related to a different mechanism; thus, further studies are necessary to obtain further details for this effect. Our research has shown that, depending on the concentration, PEGylated curcumin inhibited the cell cycle in the G2/M phase and induced the expression of proteins involved in cell cycle regulation, cell proliferation, and response to hypoxic conditions. Compound **5** under hypoxic rather than normoxic conditions increased the expression of stress-related proteins related to c-Jun N-terminal kinase signaling, angiogenesis, ECM modeling, and the p21 signaling pathway. These results make compound **5** an interesting lead compound for further research, which, if continued in the future, could allow us to better understand the anticancer mechanism.

## Figures and Tables

**Figure 5:**
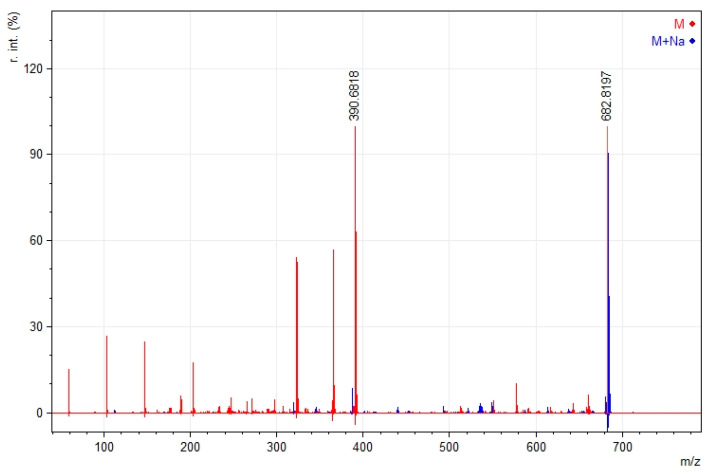
Mass spectrum of compound **5** with the presentation of fragmentations; [M+Na]^+^ sodium adduct with molecular ion in blue, and [M]^+^ molecular ion in red.

**Table 1:** The IC_50_ values for tested compounds under normoxic and hypoxic conditions against 5637, SCaBER, and MRC-5 cells. Data presented as mean ±SD from at least two independent experiments.

		IC_50_ [µM]				
Compounds	Incubation Time [h]	Normoxia			Hypoxia	
		5637	SCaBER	MRC-5	5637	SCaBER
4	24	3.31 ± 0.34	4.18 ± 1.30	>10	2.58 ± 0.11	2.42 ± 0.23
	48	3.27 ± 0.45	4.57 ± 2.07	>10	2.96 ± 0.23	2.86 ± 0.62
5	24	8.40 ± 0.21	8.53 ± 0.16	>10	6.43 ± 0.26	7.27 ± 0.20
	48	7.91 ± 0.46	7.97 ± 0.40	>10	5.02 ± 1.06	7.58 ± 0.91
6	24	2.60 ± 0.99	4.11 ± 1.40	>10	2.37 ± 0.19	5.85 ± 1.98
	48	2.63 ± 0.77	4.35 ± 1.97	9.34 ± 0.76	2.66 ± 0.07	7.28 ± 1.81
10	24	2.25 ± 0.08	2.59 ± 0.16	>10	1.96 ± 0.47	2.35 ± 0.22
	48	2.17 ± 0.24	2.71 ± 0.34	7.84 ± 0.15	2.12 ± 0.33	2.60 ± 0.03
Curcumin (11)	24	12.65 ± 3.03	13.14 ± 0.68	70.35 ± 12.45	12.14 ± 0.16	11.60 ± 0.83
	48	12.86 ± 2.35	12.57 ± 0.62	45.33 ± 3.18	13.29 ± 0.54	11.33 ± 0.19
12	24	2.48 ± 0.55	2.28 ± 0.13	9.63 ± 2.61	2.32 ± 0.29	3.26 ± 1.05
	48	2.40 ± 0.45	2.21 ± 0.17	5.80 ± 0.77	2.64 ± 0.54	4.25 ± 1.94
Isocurcumin (13)	24	10.85 ± 3.46	14.66 ± 0.88	>20	13.09 ± 2.89	14.17 ± 0.23
	48	6.71 ± 1.70	14.26 ± 0.41	>20	10.12 ± 3.12	11.94 ± 0.09

**Table 2:** The prediction of ADMET properties for compounds **4**, **5**, **10**, and curcumin.

	Compound			
Parameter	4	5	10	Curcumin
TPSA ^a,^*	118.6	129.6	85.22	93.06
logP ^b,^*	4.94	4.56	4.24	3.37
Subcellular Localization **	Mitochondria	Mitochondria	Mitochondria	Mitochondria
Caco-2 permeability * LogPapp cm/s **	1.0738	1.4124	0.8938	1.2769
BBB ^c,^**	+	+	+	-
Carcinogenicity **	-	-	+	-
AOT ^d,^**	III	IV	III	III
P-glycoprotein Substrate **	Substrate	Substrate	Substrate	Substrate
P-glycoprotein Inhibitor **	Inhibitor	Inhibitor	Non-inhibitor	Non-inhibitor

* calculated using ProTox-II, ** calculated using admetSAR, ^a^ topological polar surface area, ^b^ octanol/water partition coefficient (log P), ^c^ blood–brain barrier, and ^d^ acute oral toxicity.

## Data Availability

Not applicable.

## Supplementary Materials

The following supporting information can be downloaded at: https://www.mdpi.com/article/10.3390/ijms24021467/s1.
