# Supplementary material for: Enhanced Cytotoxic Activity of PEGylated Curcumin Derivatives: Synthesis, Structure–Activity Evaluation, and Biological Activity
Source: Int J Mol Sci. 2023 Jan 11;24(2):1467. doi: 10.3390/ijms24021467 (PMC9867315; doi:10.3390/ijms24021467)
Supplement: Supplementary file 1 [file ijms-24-01467-s001.zip › ijms-2055194-supplementary.pdf]

Enhanced Cytotoxic Activity of PEGylated Curcumin Derivatives: Synthesis, Structure–Activity Evaluation, and Biological Activity

Dawid Lazewski [1], Malgorzata Kucinska [2,*], Edward Potapskiy [1], Joanna Kuzminska [3], Lukasz Popenda [4], Artur Tezyk [5], Tomasz Goslinski [1], Marcin Wierzchowski [1] and Marek Murias [2,6,*]

1 Department of Chemical Technology of Drugs, Poznan University of Medical Sciences, Grunwaldzka 6 Street, 60-780 Poznan, Poland; lazewskidawid@gmail.com (D.L.); potapskiyed@gmail.com (E.P.); tomasz.goslinski@ump.edu.pl (T.G.); mwierzch@ump.edu.pl (M.W.)
2 Department of Toxicology, Poznan University of Medical Sciences, Dojazd 30 Street, 60-631 Poznan, Poland
3 Department of Pharmaceutical Chemistry, Poznan University of Medical Sciences, Grunwaldzka 6 Street, 60-780 Poznan, Poland; joanna.kuzminska@student.ump.edu.pl
4 NanoBioMedical Centre, Adam Mickiewicz University, Wszechnicy Piastowskiej 3 Street, 61-614 Poznan, Poland; lpopenda@amu.edu.pl
5 Department of Forensic Medicine, Poznan University of Medical Sciences, Rokietnicka 10 Street, 60-806 Poznan, Poland; atezyk@ump.edu.pl
6 Center for Advanced Technology, Adam Mickiewicz University, Uniwersytetu Poznanskiego 10 Street, 61-614 Poznan, Poland
* Correspondence: kucinska@ump.edu.pl (M.K.); marek.murias@ump.edu.pl (M.M.)

Supporting Information

Citation: Lazewski, D.; Kucinska, M.; Potapskiy, E.; Kuzminska, J.; Popenda, L.; Tezyk, A.; Goslinski, T.; Wierzchowski, M.; Murias, M. Enhanced Cytotoxic Activity of PEGylated Curcumin Derivatives: Synthesis, Structure–Activity Evaluation, and Biological Activity. *Int. J. Mol. Sci.* **2023**, *24*, 1467. https://doi.org/10.3390/ijms24021467

Academic Editor(s): Hwan-Suck Chung

Received: 9 November 2022
Revised: 25 December 2022
Accepted: 6 January 2023
Published: 11 January 2023

Copyright: © 2023 by the authors. Submitted for possible open access publication under the terms and conditions of the Creative Commons Attribution (CC BY) license (https://creativecommons.org/licenses/by/4.0/).

Table of contents

S1 – NMR spectroscopy....................2
S2 – Mass spectrometry....................22
S3 – UV-vis spectroscopy....................26
S4 – HPLC data....................28

S1 – NMR spectroscopy

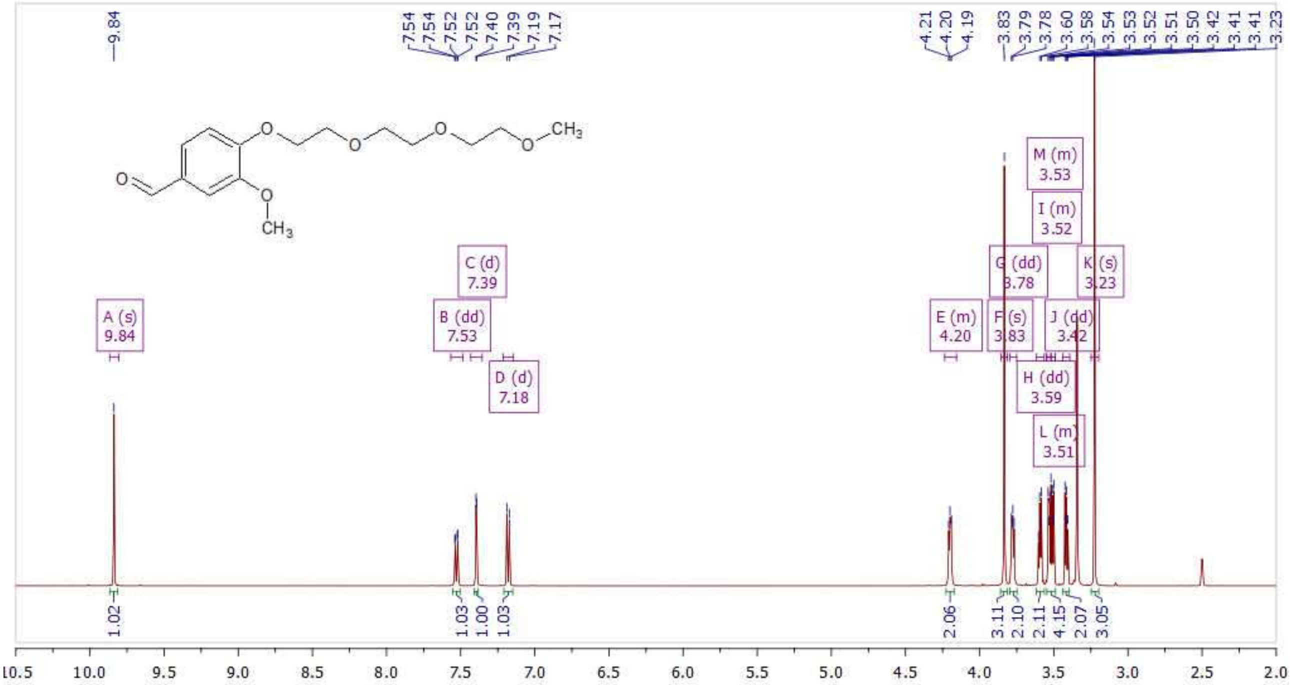

Figure S1. ^{1}H NMR spectrum of aldehyde **1**.

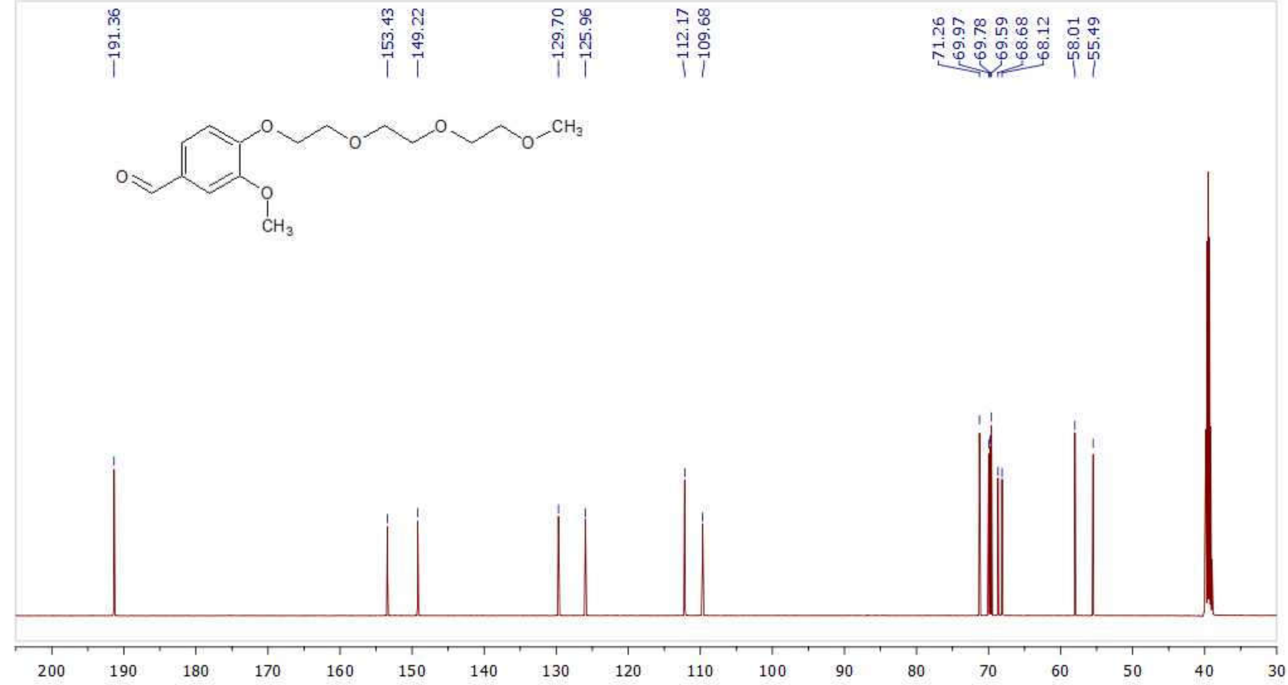

Figure S2. ^{13}C NMR spectrum of aldehyde **1**.

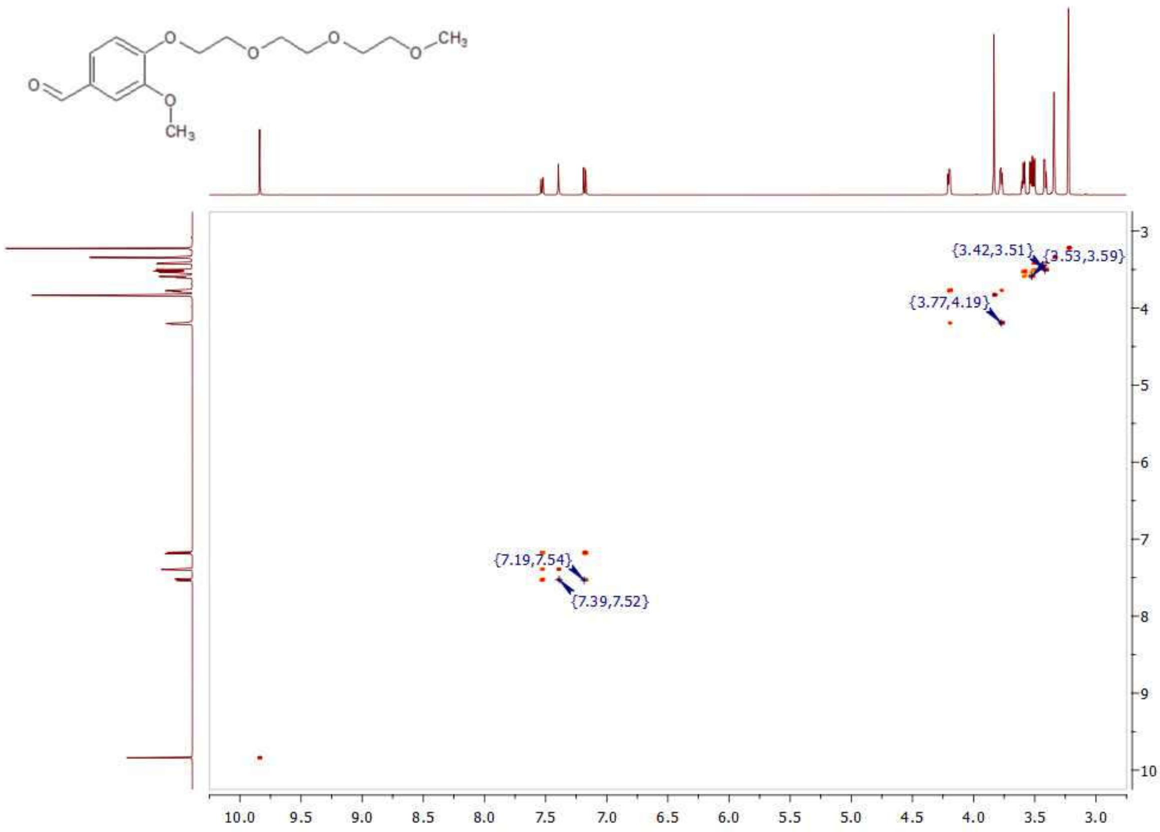

Figure S3. ^{1}H-^{1}H COSY spectrum of aldehyde **1**.

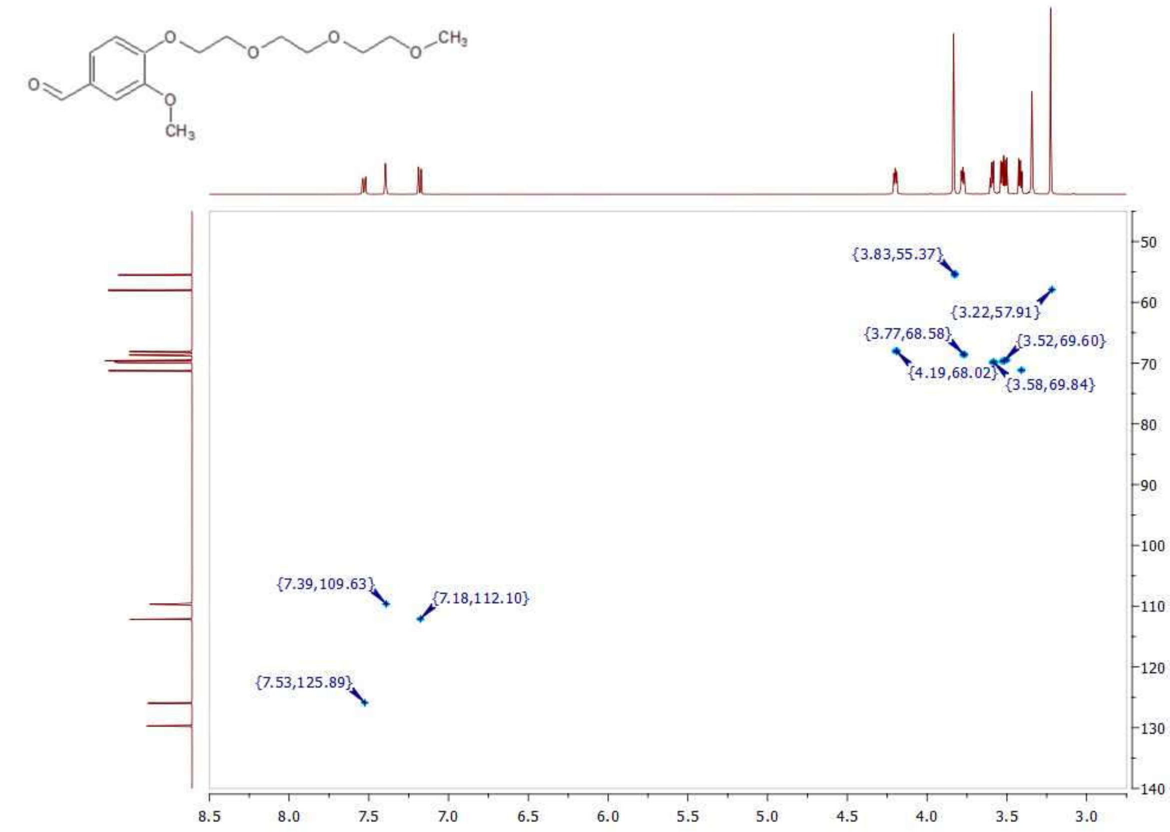

Figure S4. ^{1}H-^{13}C HSQC spectrum of aldehyde **1**.

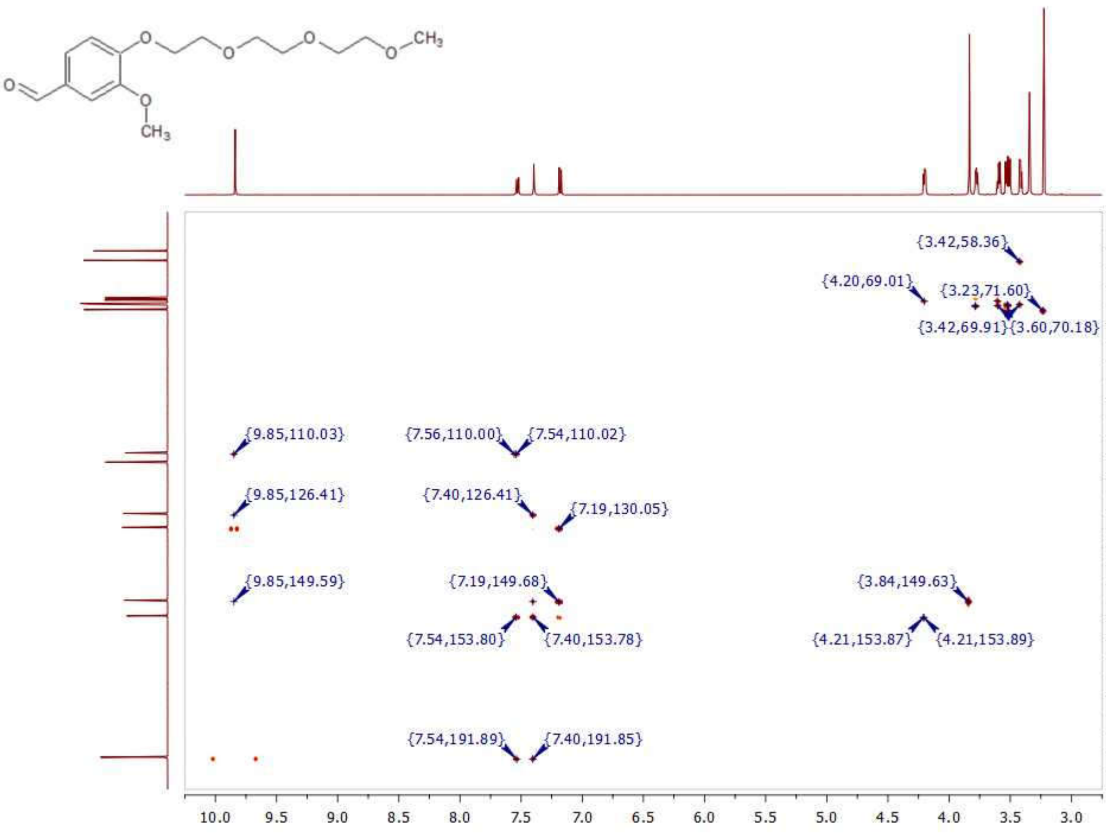

Figure S5. ^{1}H-^{13}C HMBC spectrum of aldehyde **1**.

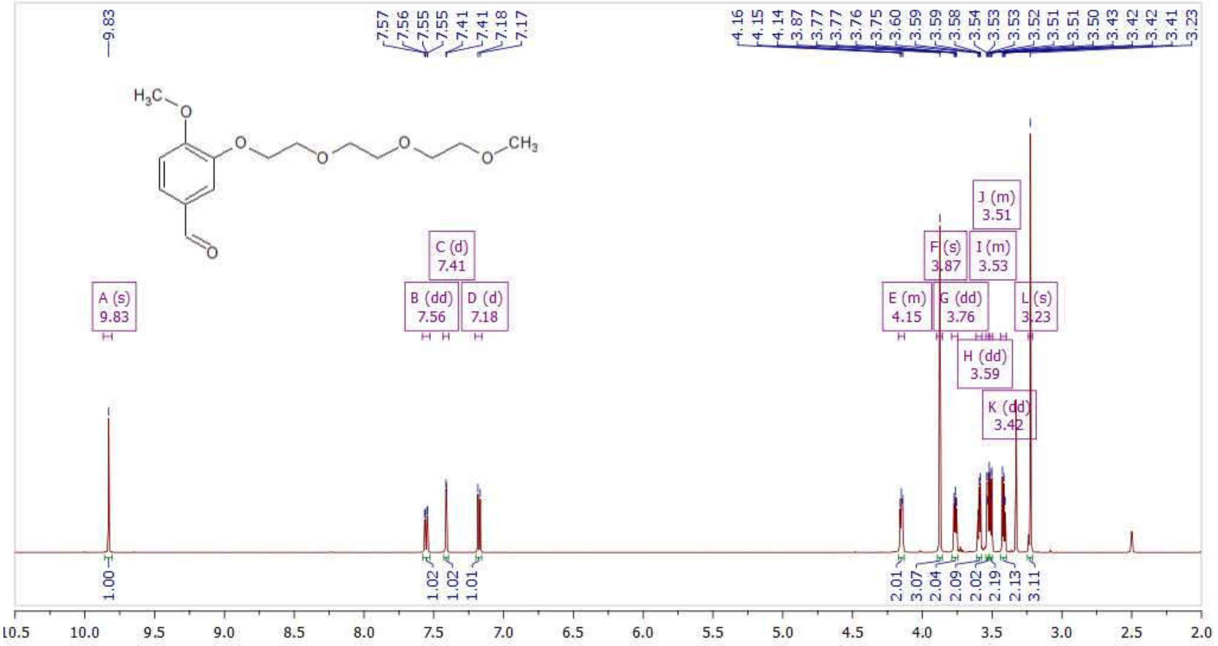

Figure S6. ^{1}H NMR spectrum of aldehyde **2**.

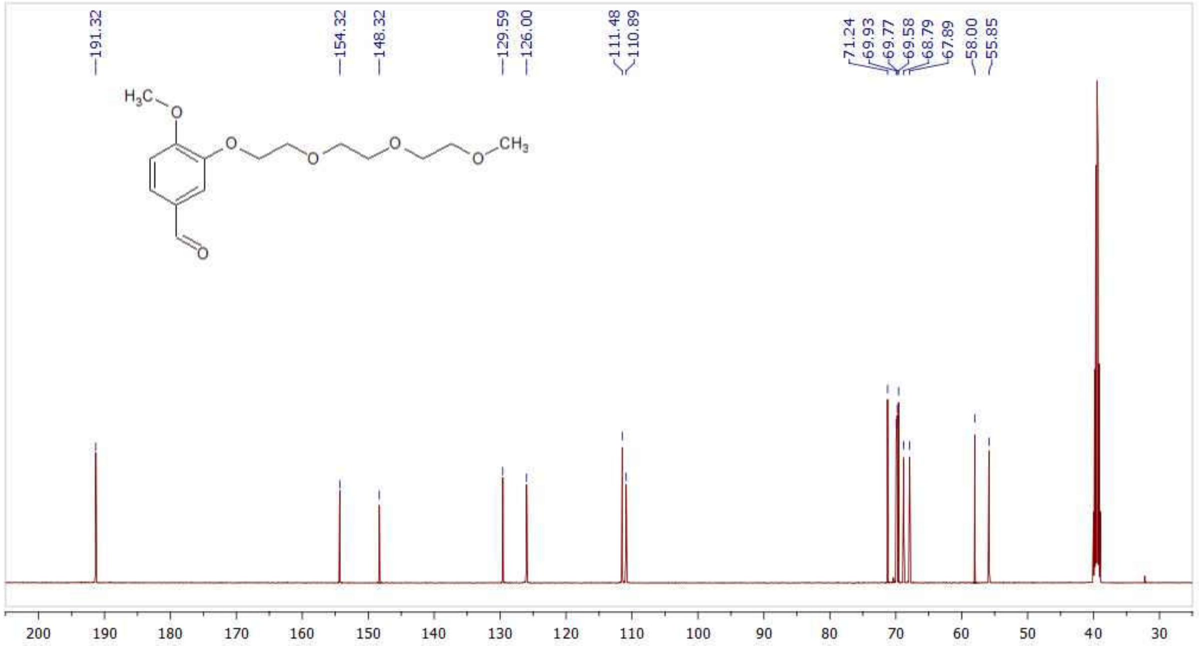

Figure S7. ^{13}C NMR spectrum of aldehyde **2**.

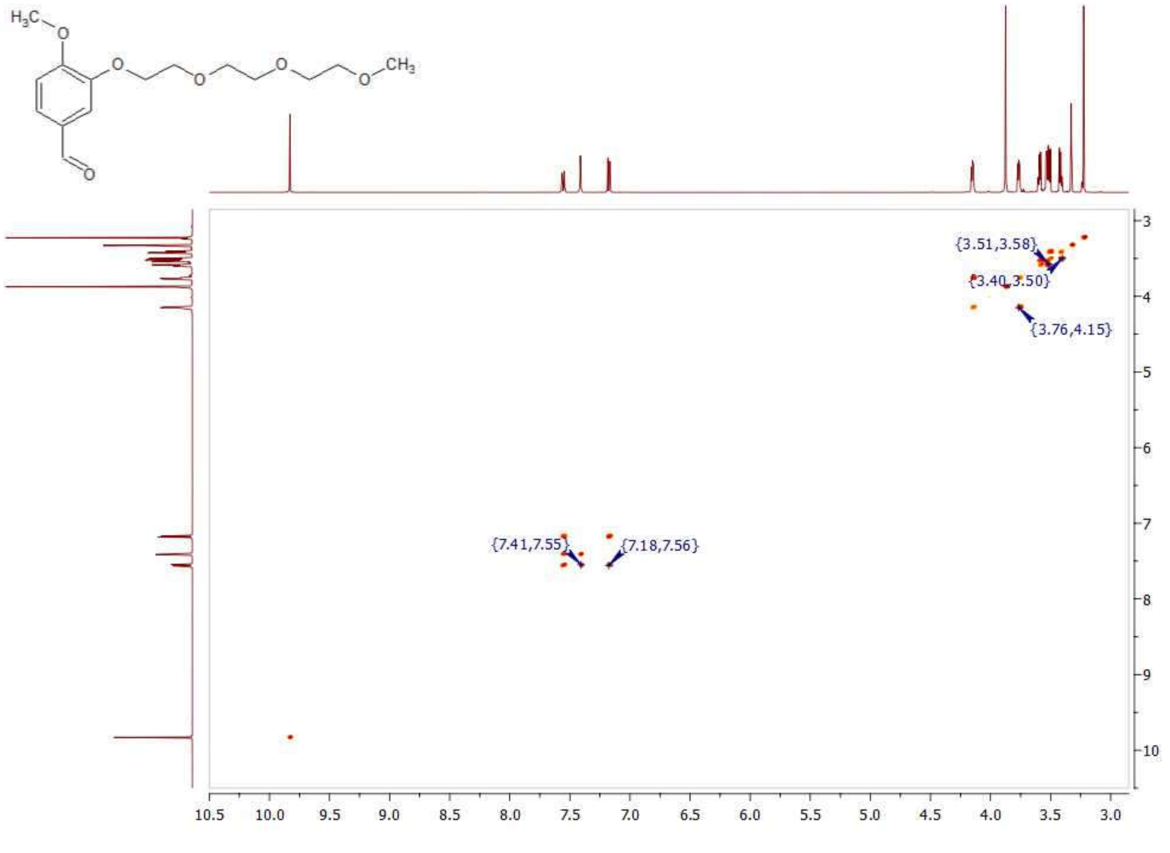

Figure S8. ^{1}H-^{1}H COSY spectrum of aldehyde **2**.

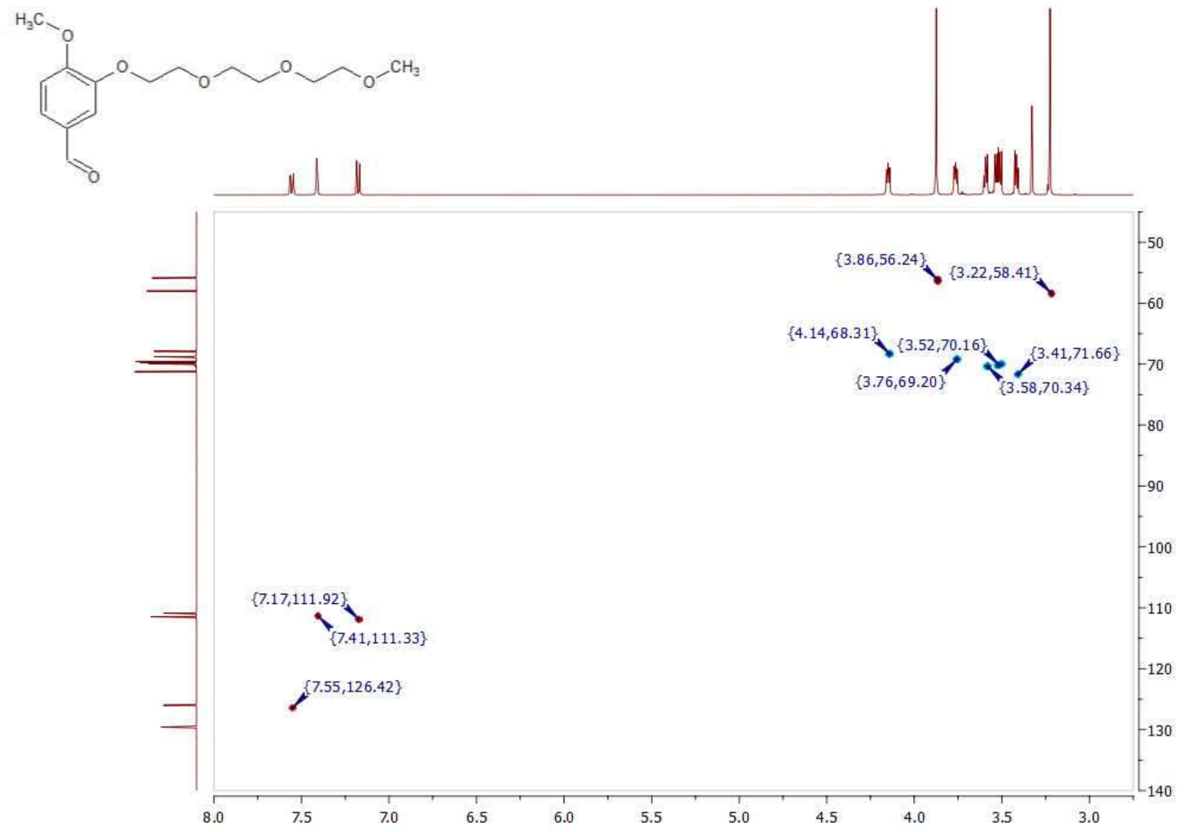

Figure S9. ^{1}H-^{13}C HSQC spectrum of aldehyde **2**.

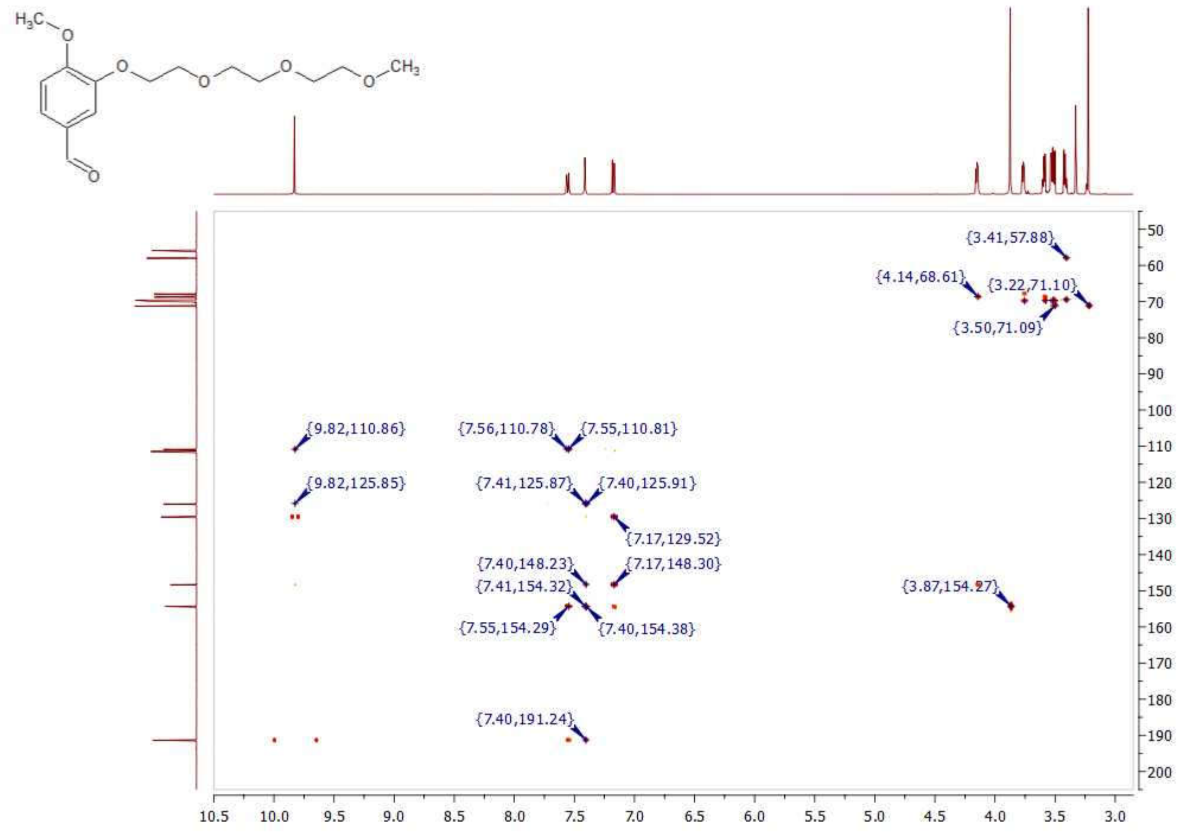

Figure S10. ^{1}H-^{13}C HMBC spectrum of aldehyde **2**.

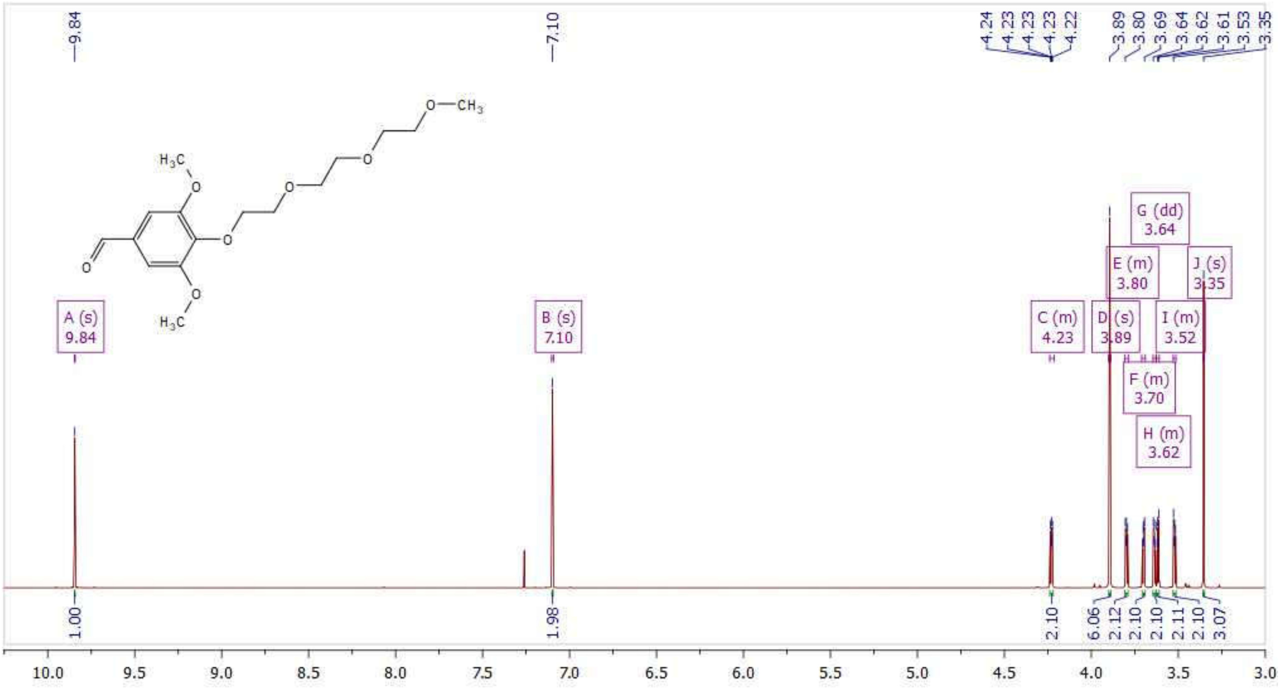

Figure S11. ^{1}H NMR spectrum of aldehyde **3**.

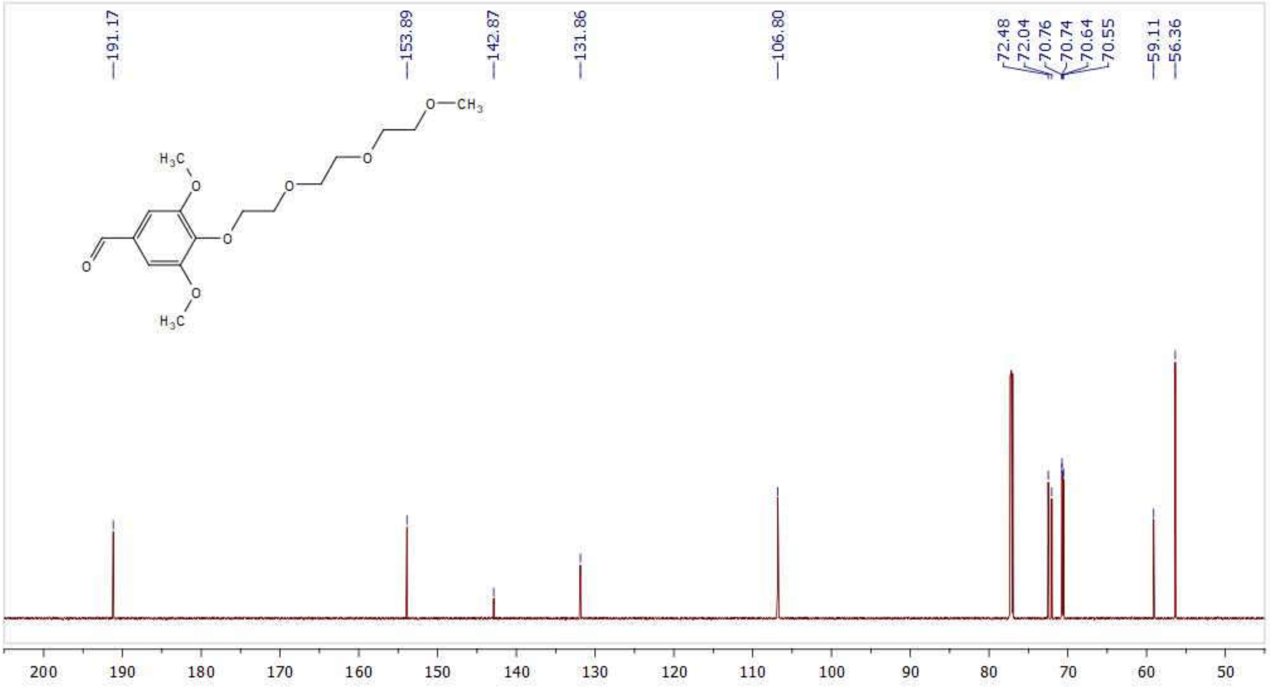

Figure S12. ^{13}C NMR spectrum of aldehyde **3**.

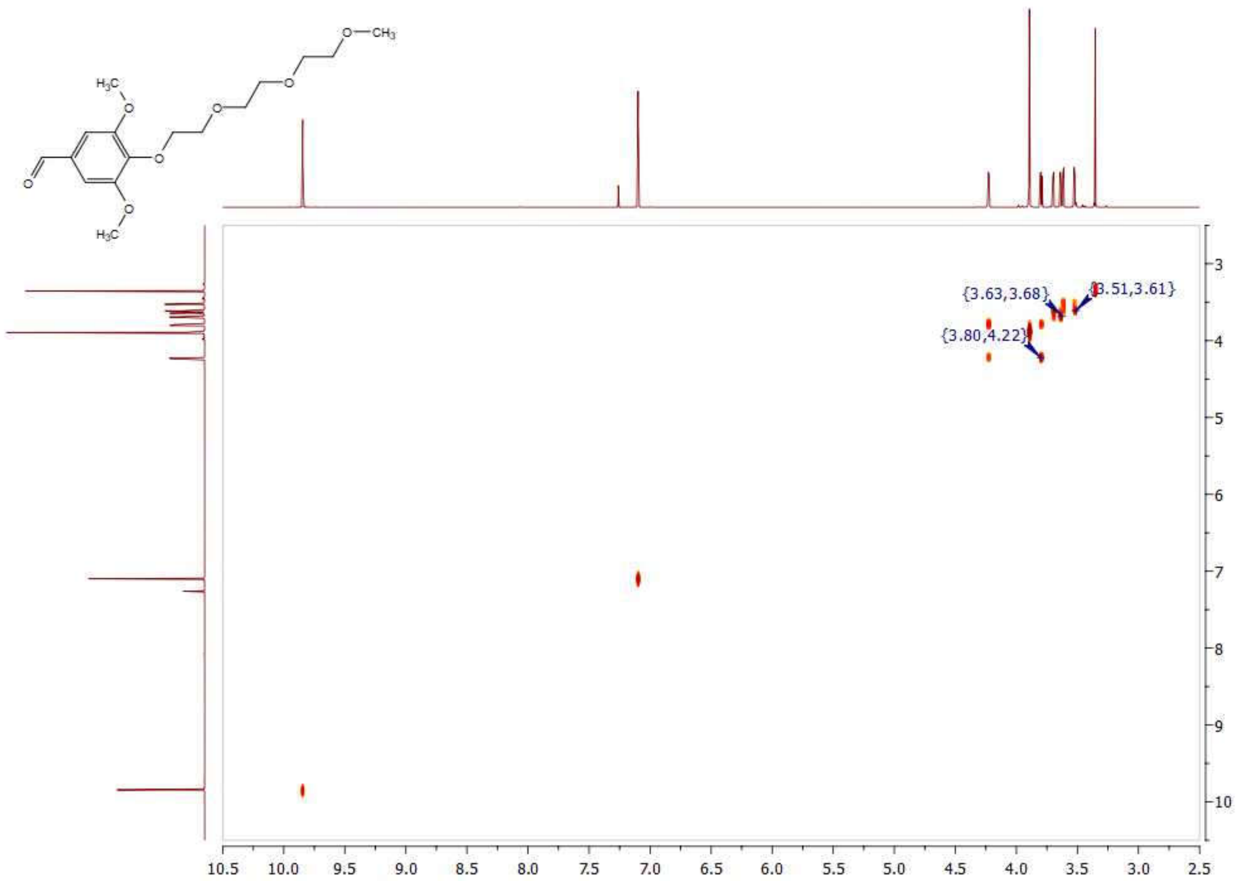

Figure S13. ^1H-^1H COSY spectrum of aldehyde **3**.

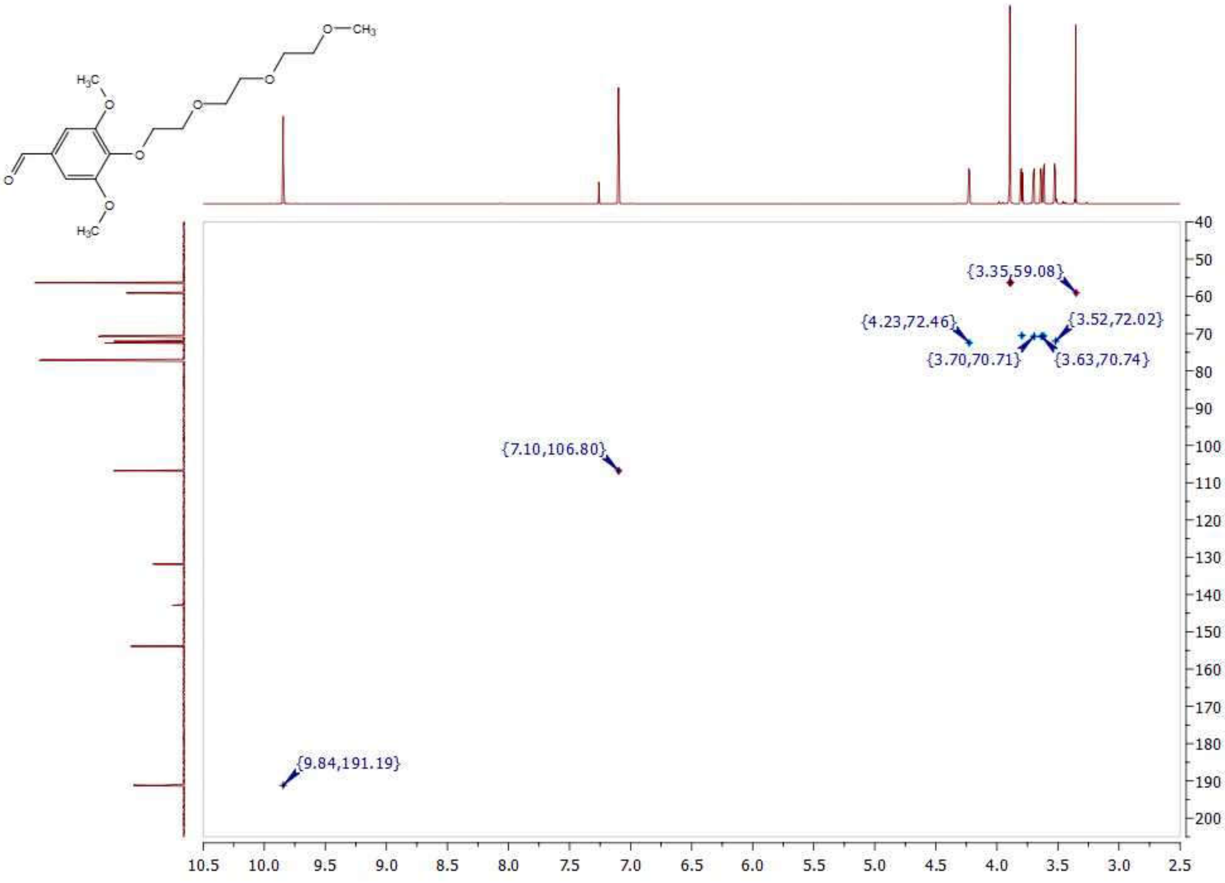

Figure S14. ^{1}H-^{13}C HSQC spectrum of aldehyde **3**.

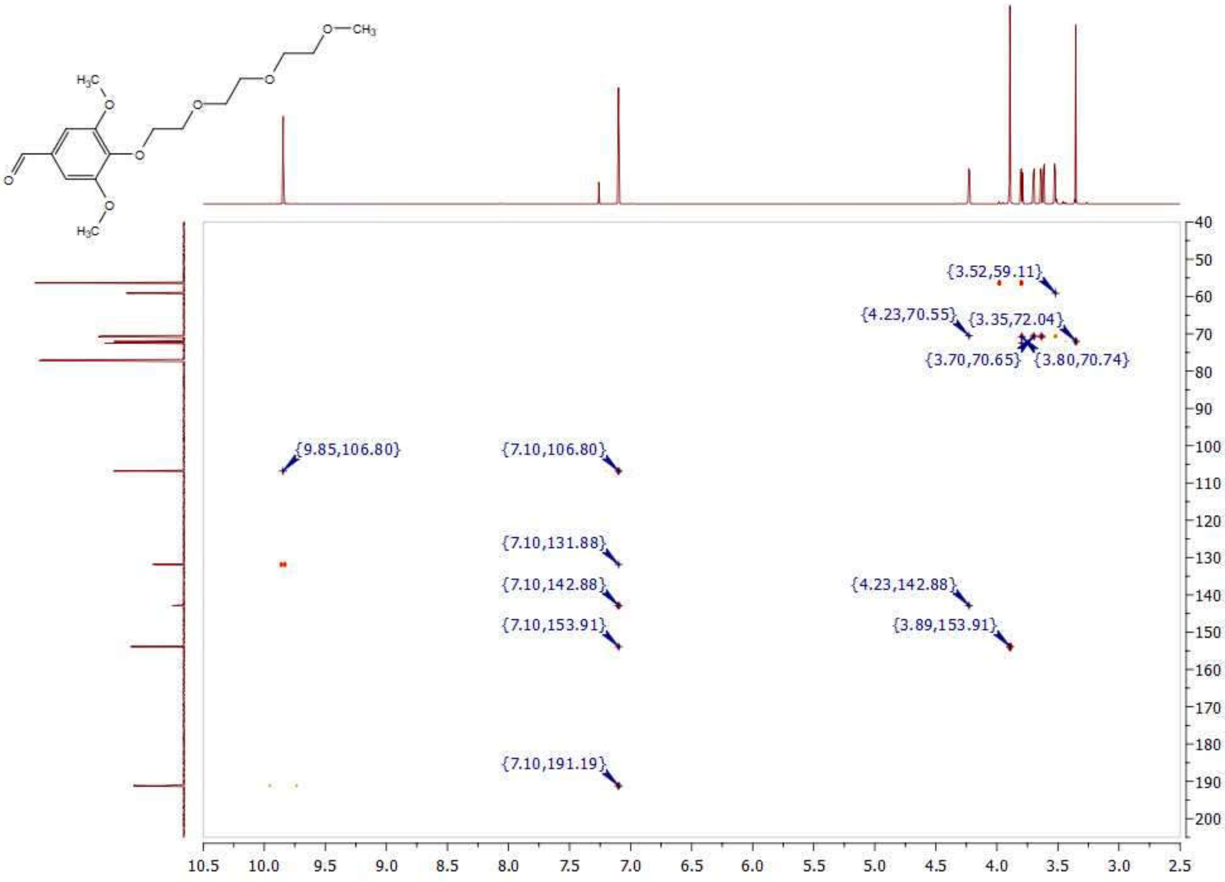

Figure S15. ^{1}H-^{13}C HMBC spectrum of aldehyde **3**.

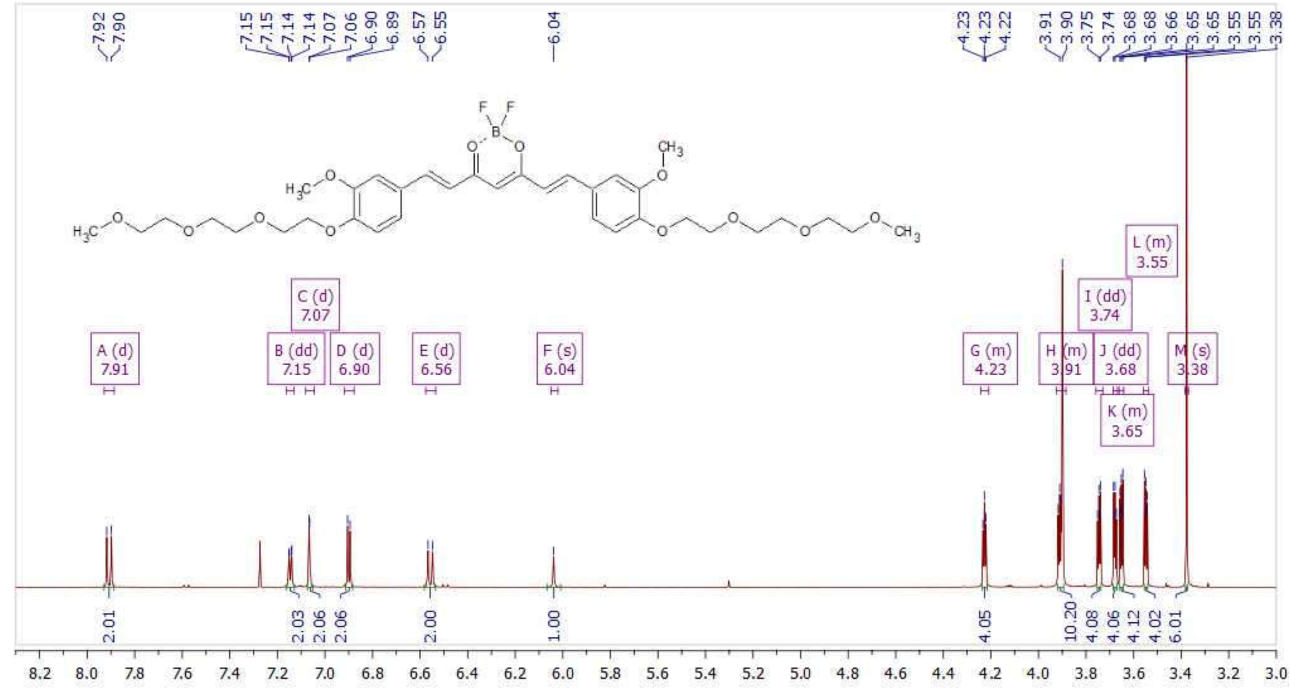

Figure S16. ^{1}H NMR spectrum of compound **4**.

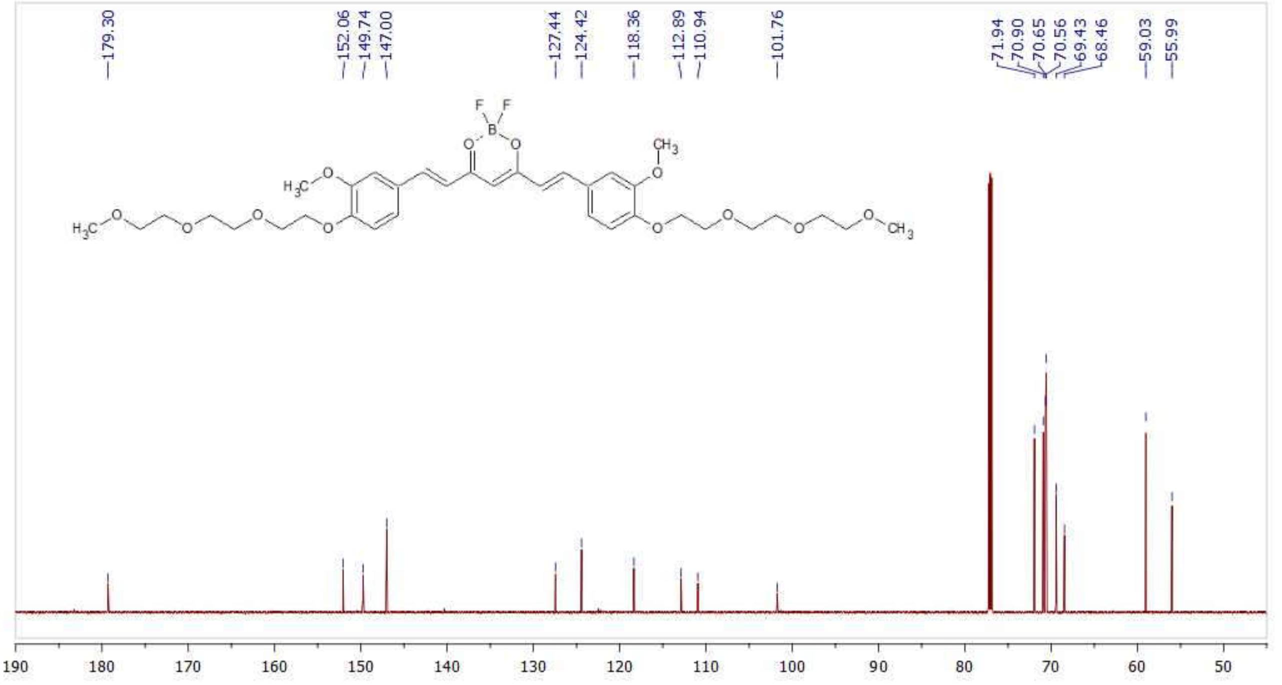

Figure S17. ^{13}C NMR spectrum of compound **4**.

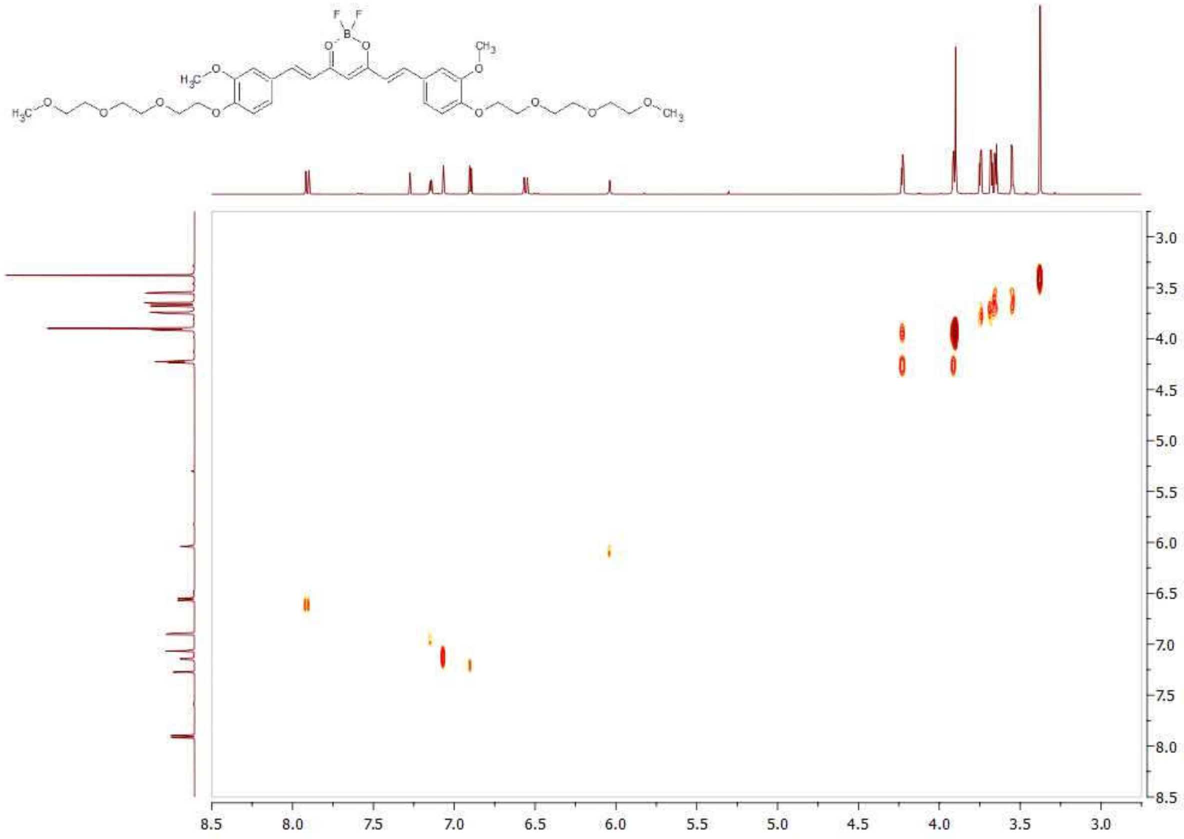

Figure S18. ^1H-^1H COSY spectrum of compound **4**.

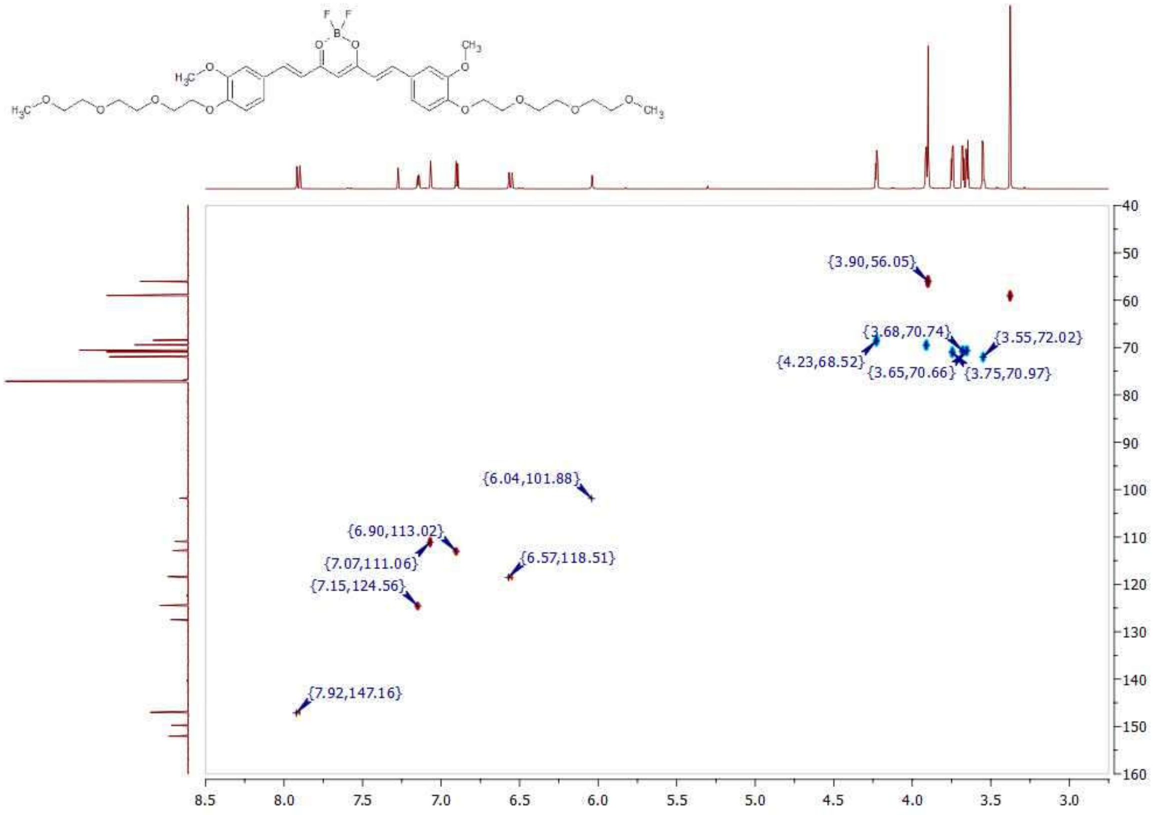

Figure S19. ^1H-^{13}C HSQC spectrum of compound **4**.

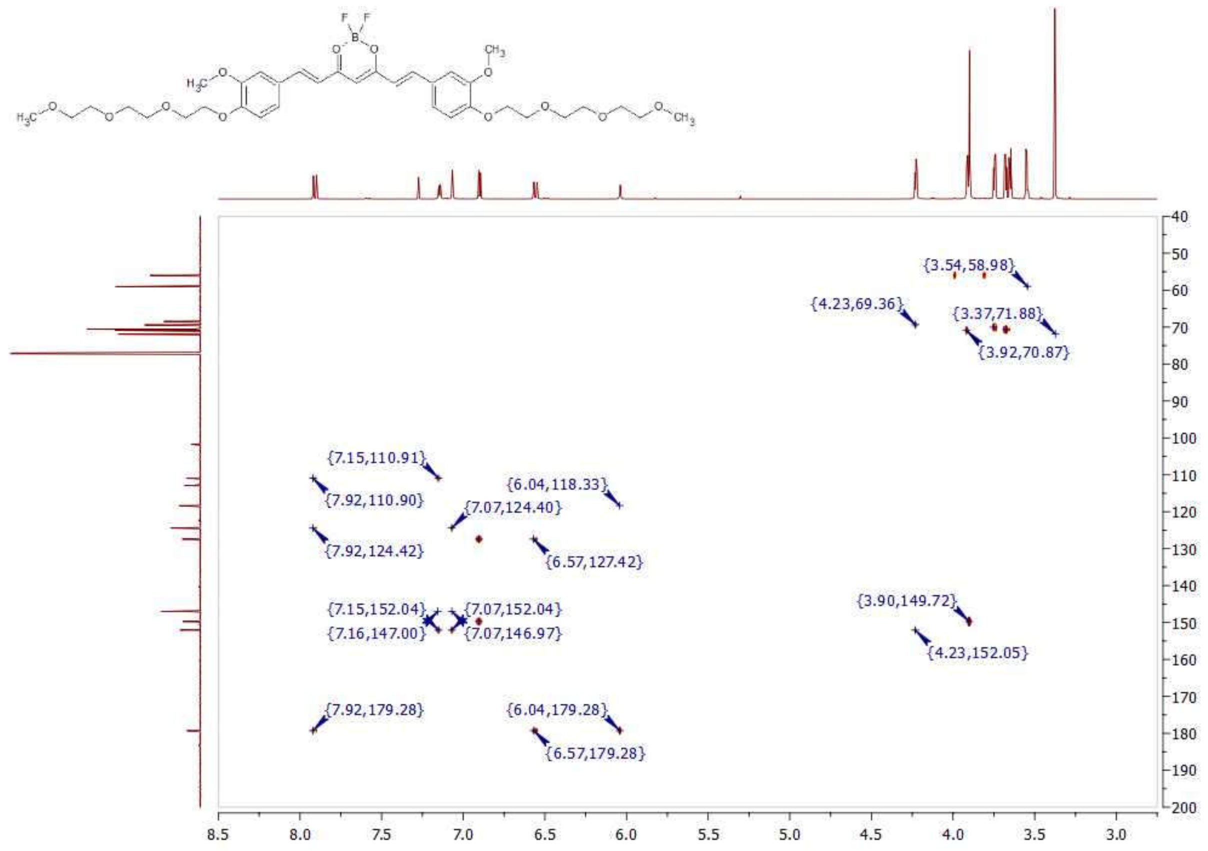

Figure S20. ^{1}H-^{13}C HMBC spectrum of compound **4**.

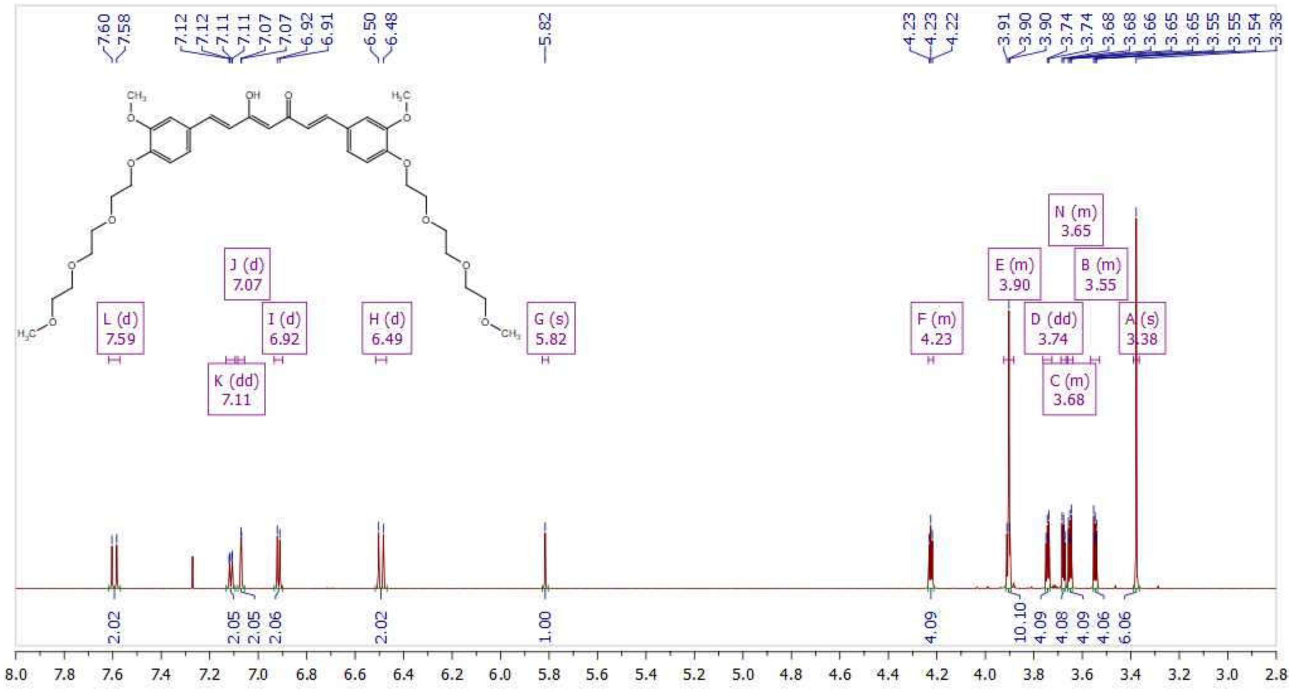

Figure S21. ^{1}H NMR spectrum of compound **5**.

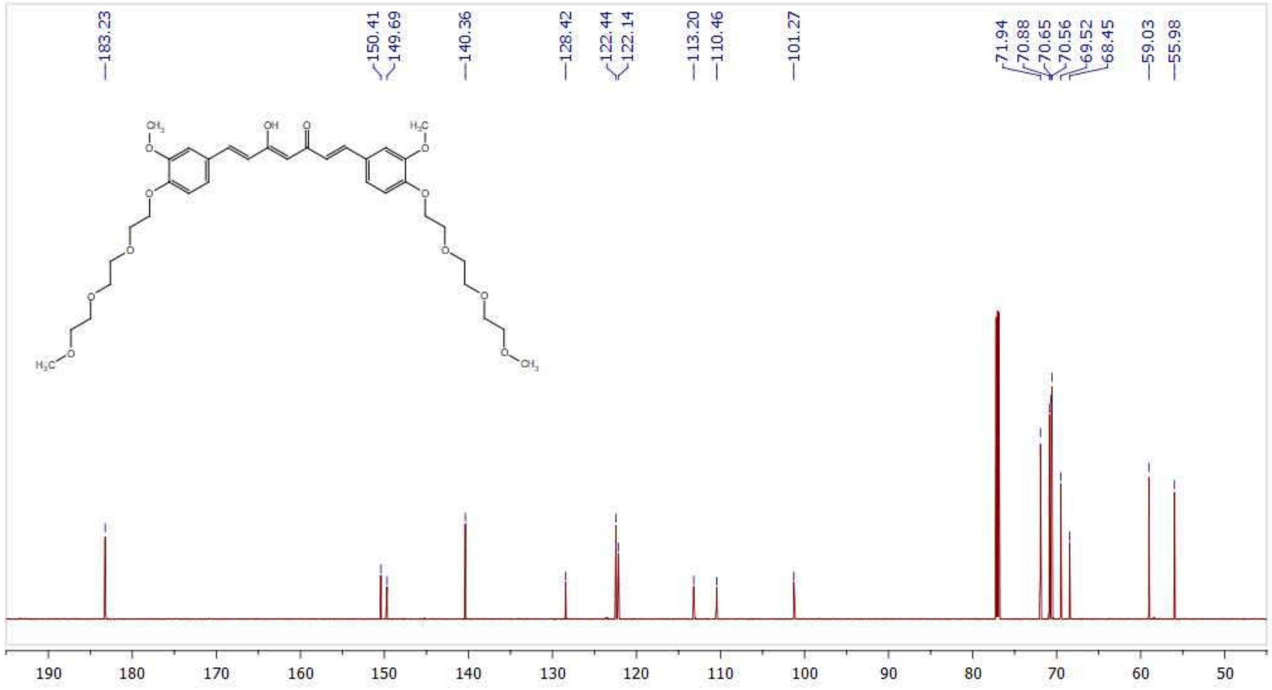

Figure S22. ^{13}C NMR spectrum of compound **5**.

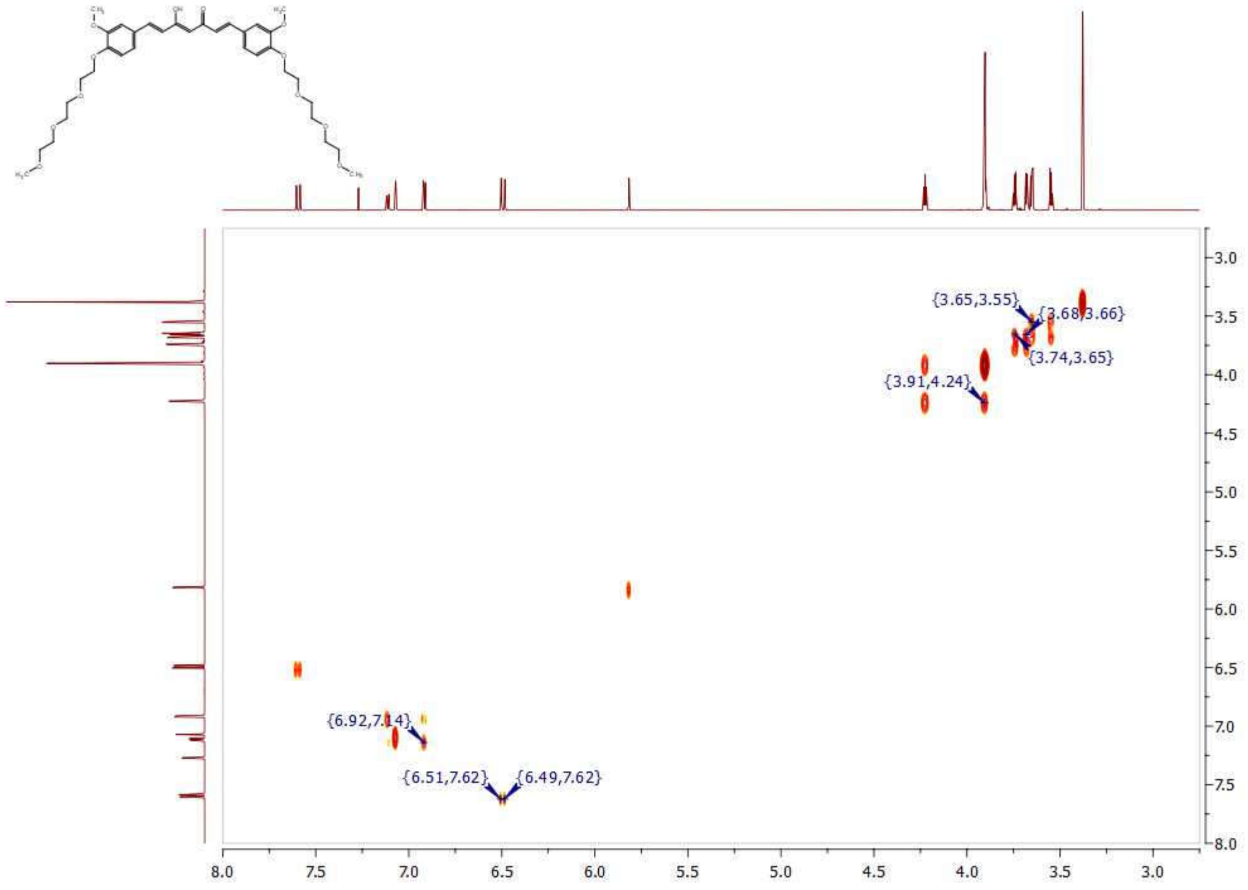

Figure S23. ^{1}H-^{1}H COSY spectrum of compound **5**.

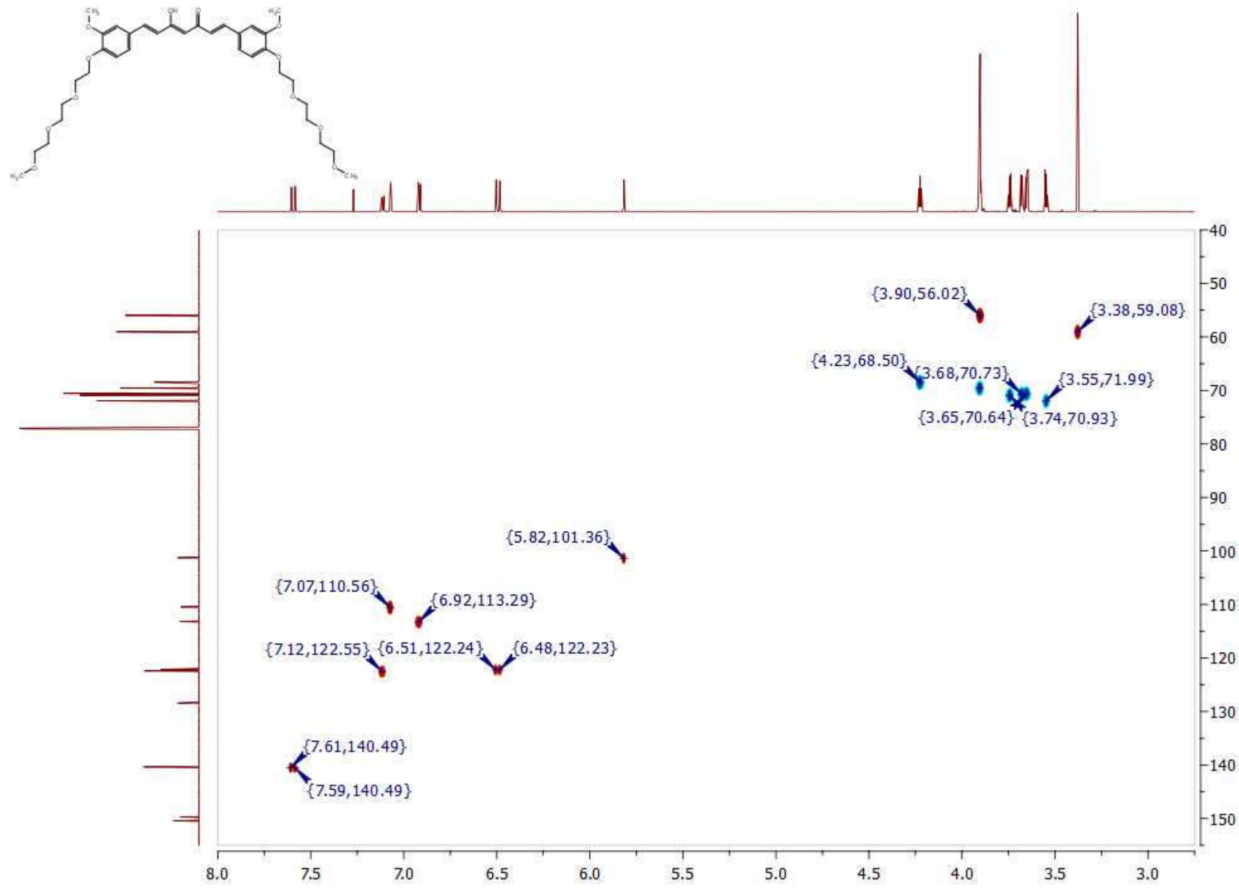

Figure S24. ^{1}H-^{13}C HSQC spectrum of compound **5**.

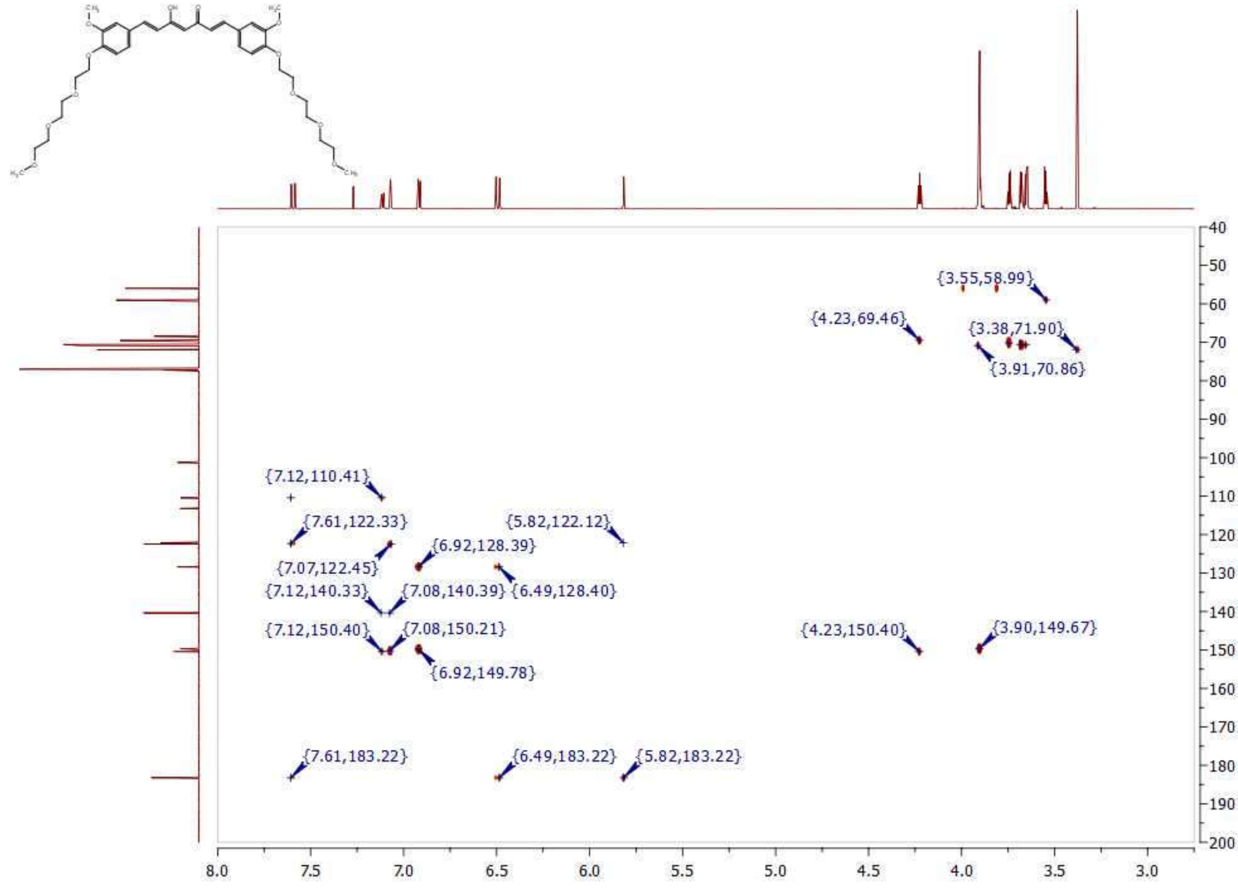

Figure S25. ^{1}H-^{13}C HMBC spectrum of compound **5**.

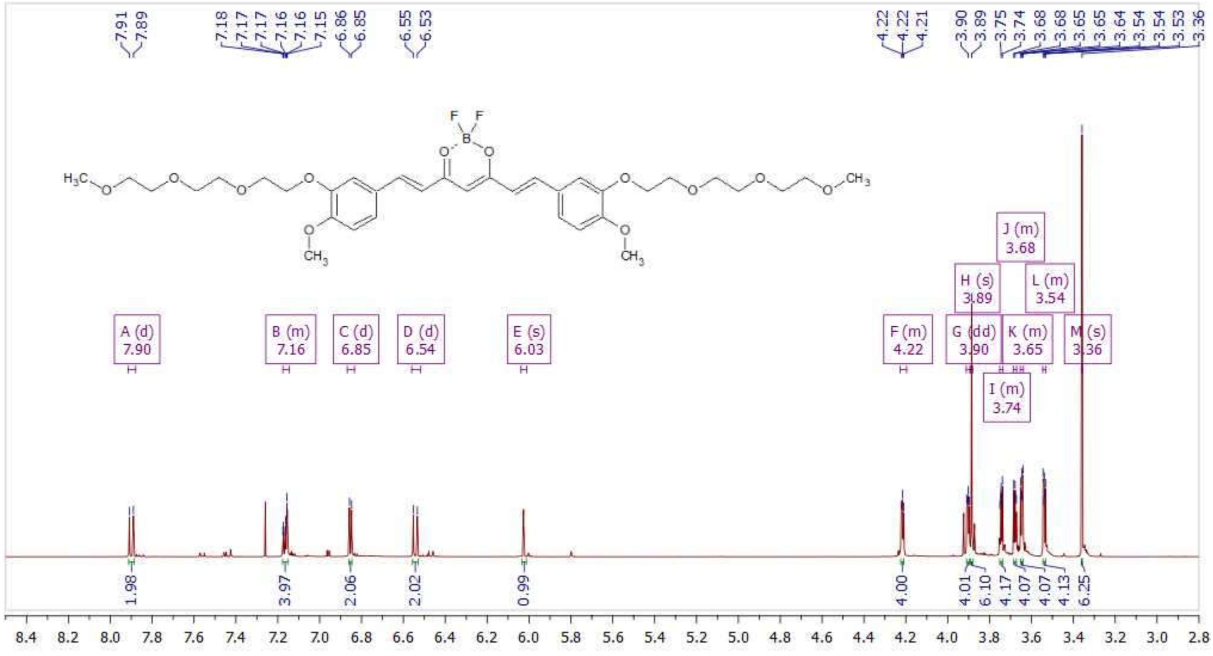

Figure S26. ^{1}H NMR spectrum of compound **6**.

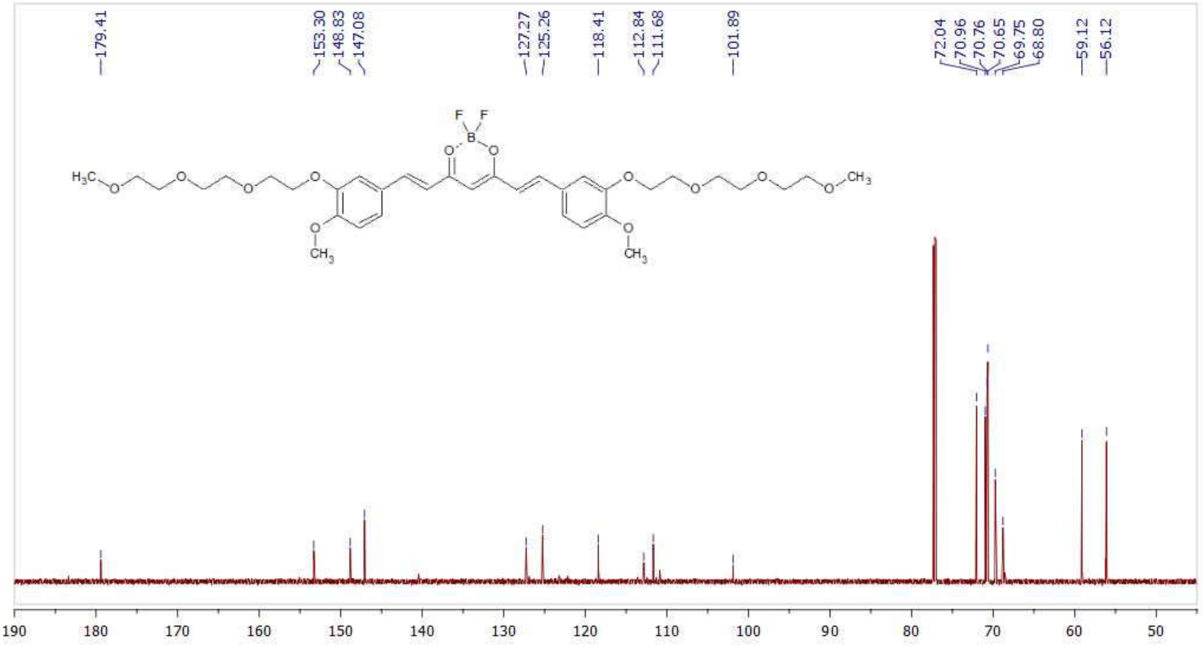

Figure S27. ^{13}C NMR spectrum of compound **6**.

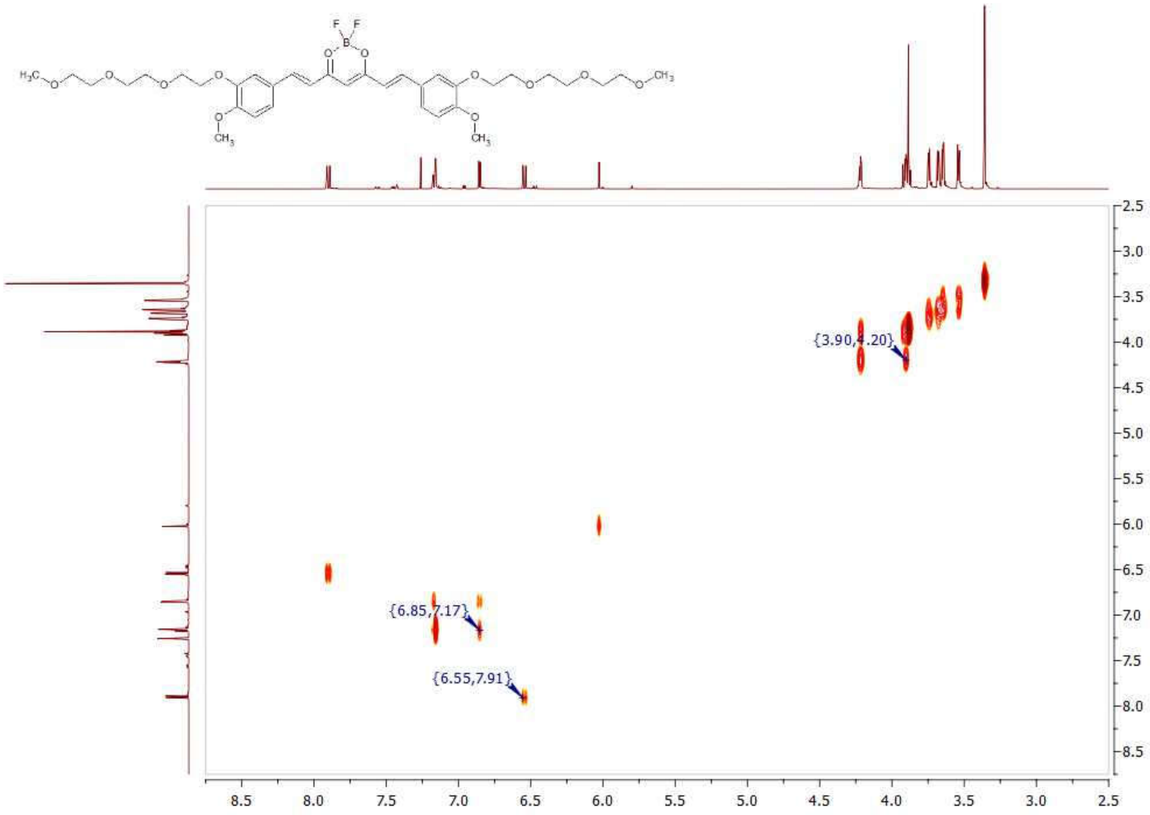

Figure S28. ^{1}H-^{1}H COSY spectrum of compound **6**.

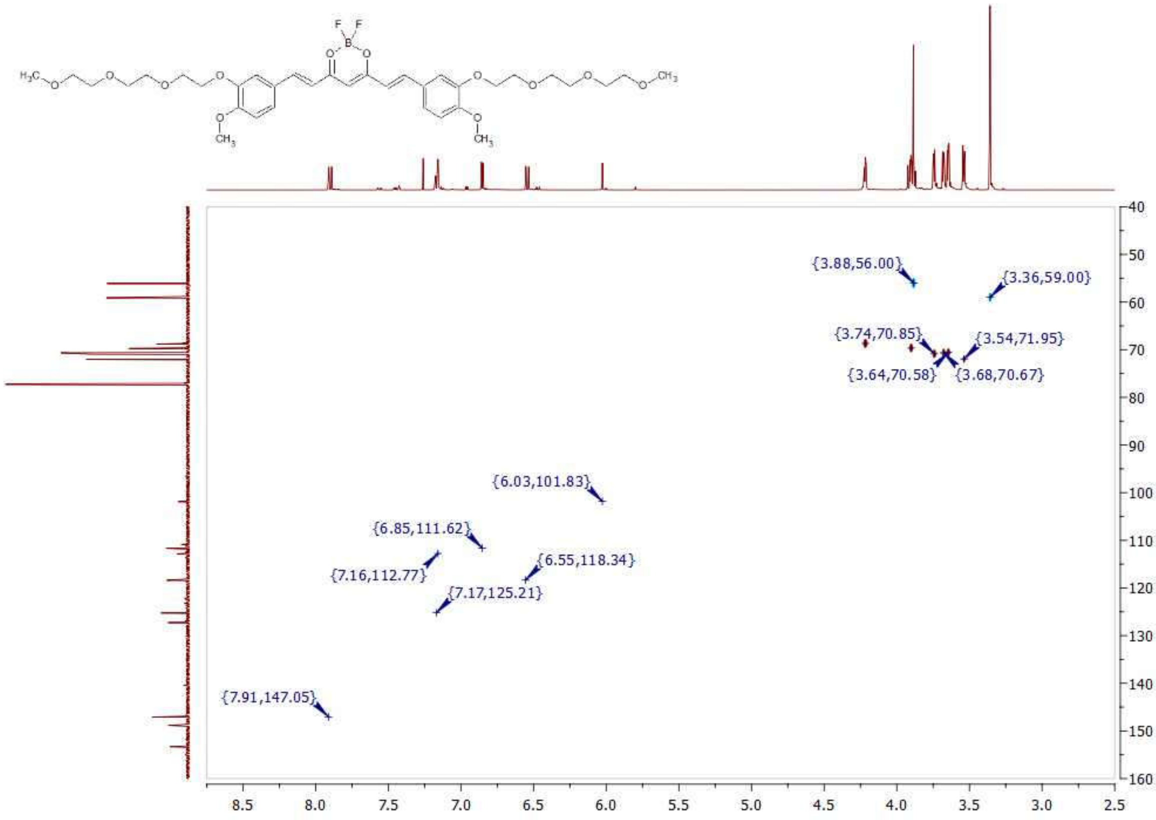

Figure S29. ^{1}H-^{13}C HSQC spectrum of compound **6**.

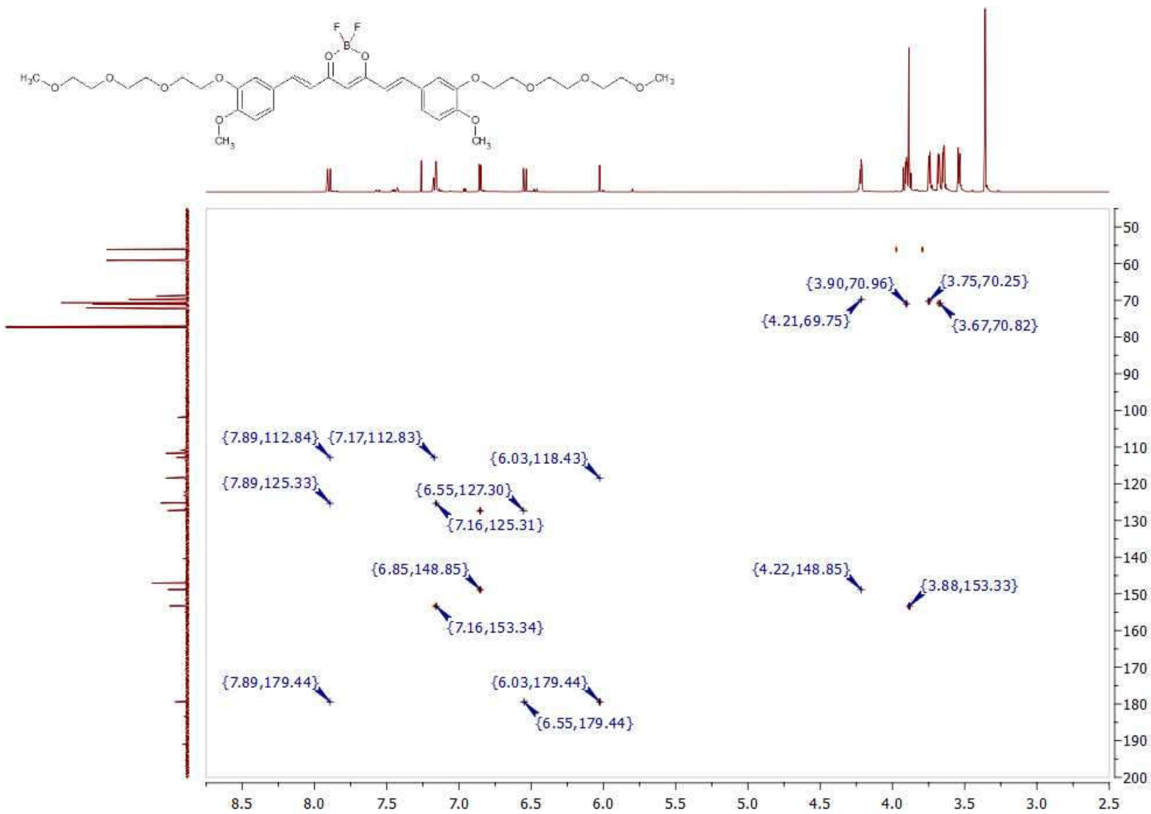

Figure S30. ^{1}H-^{13}C HMBC spectrum of compound **6**.

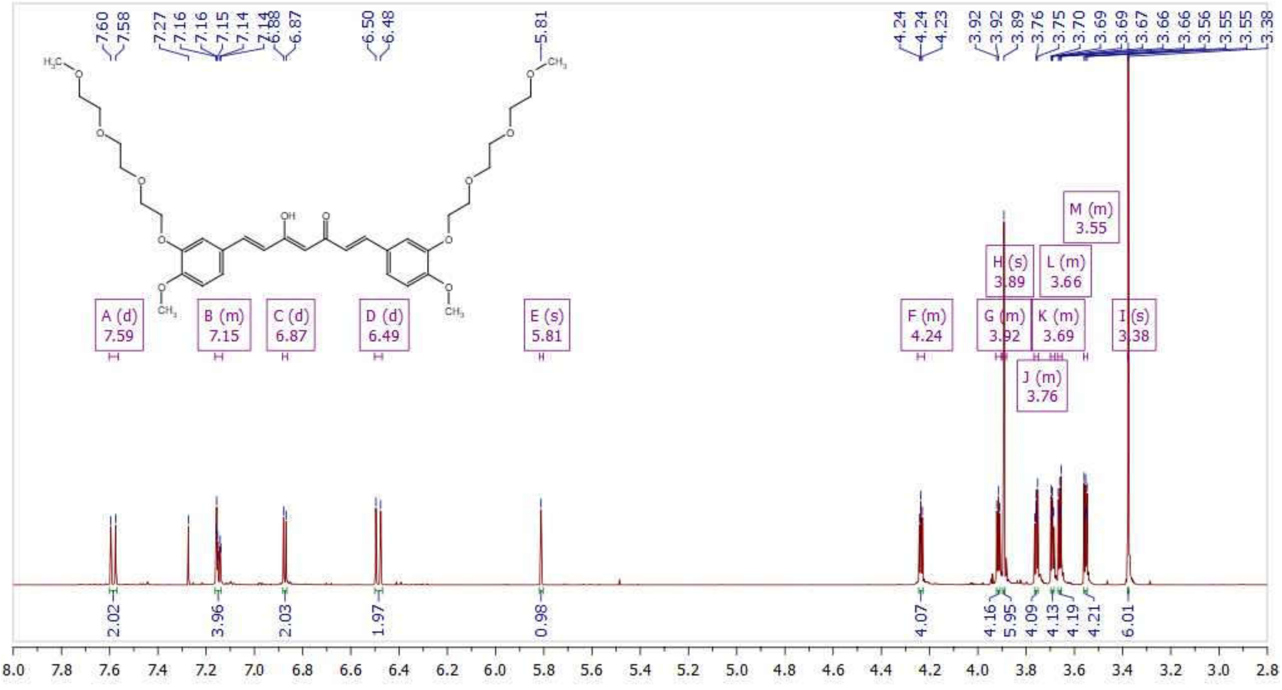

Figure S31. ^{1}H NMR spectrum of compound **7**.

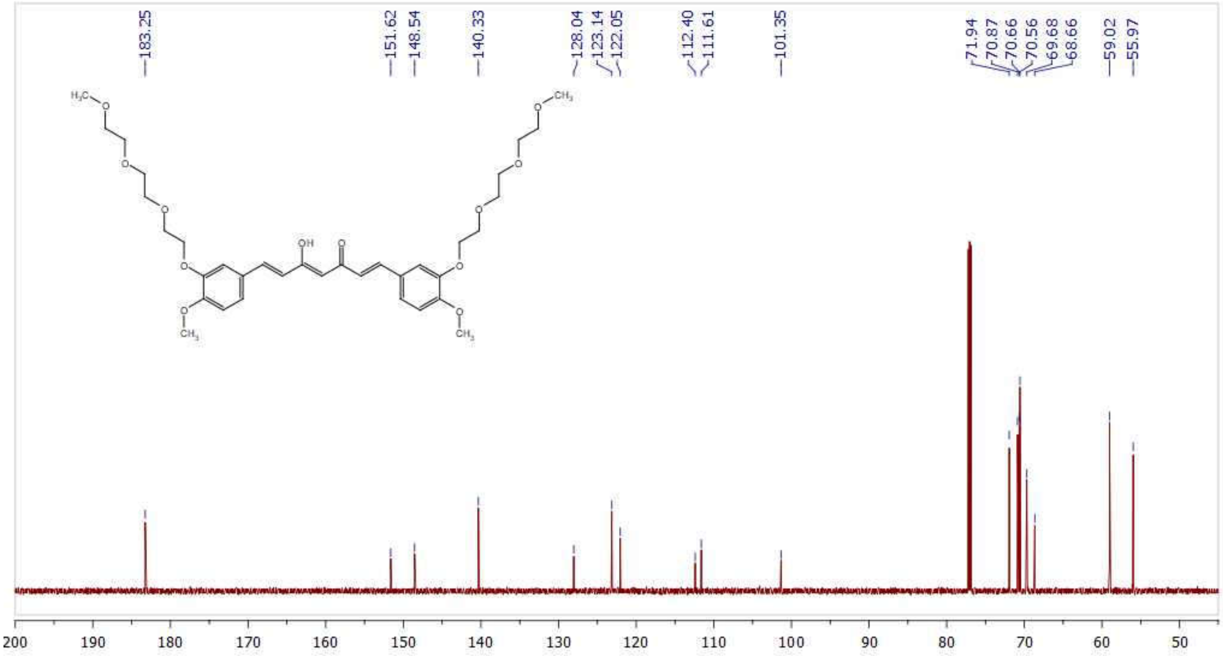

Figure S32. ^{13}C NMR spectrum of compound **7**.

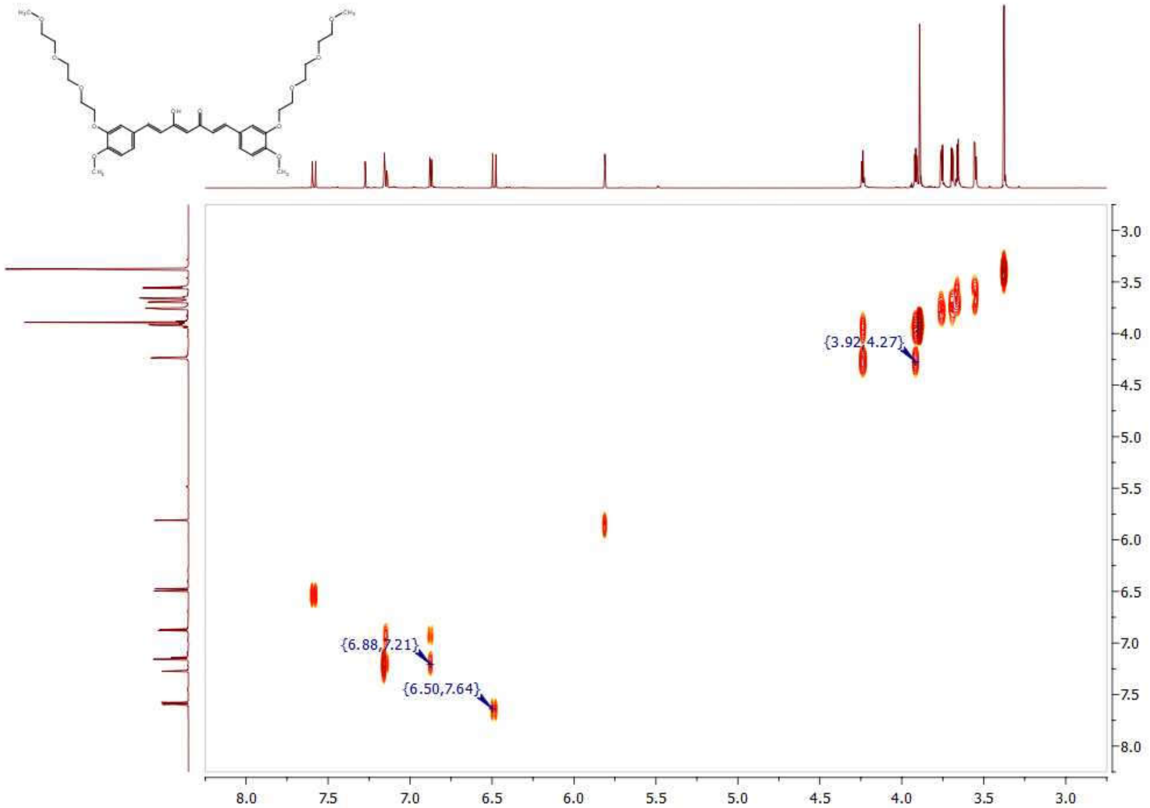

Figure S33. ^{1}H-^{1}H COSY spectrum of compound **7**.

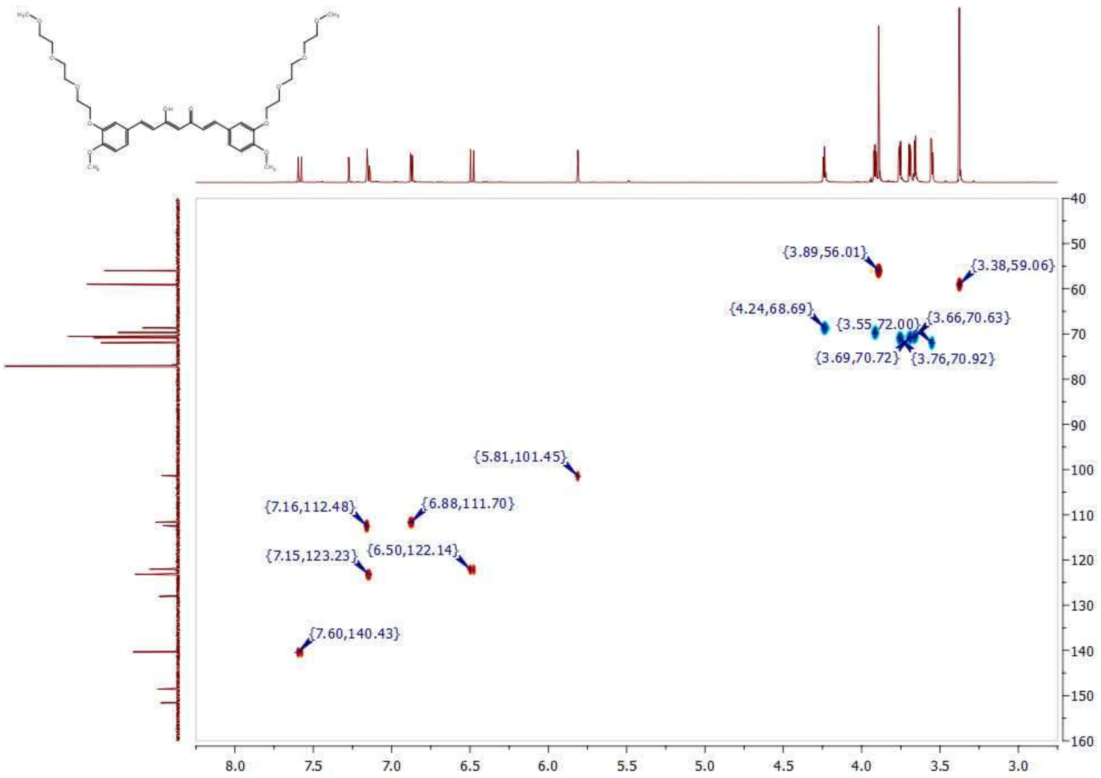

Figure S34. ^{1}H-^{13}C HSQC spectrum of compound **7**.

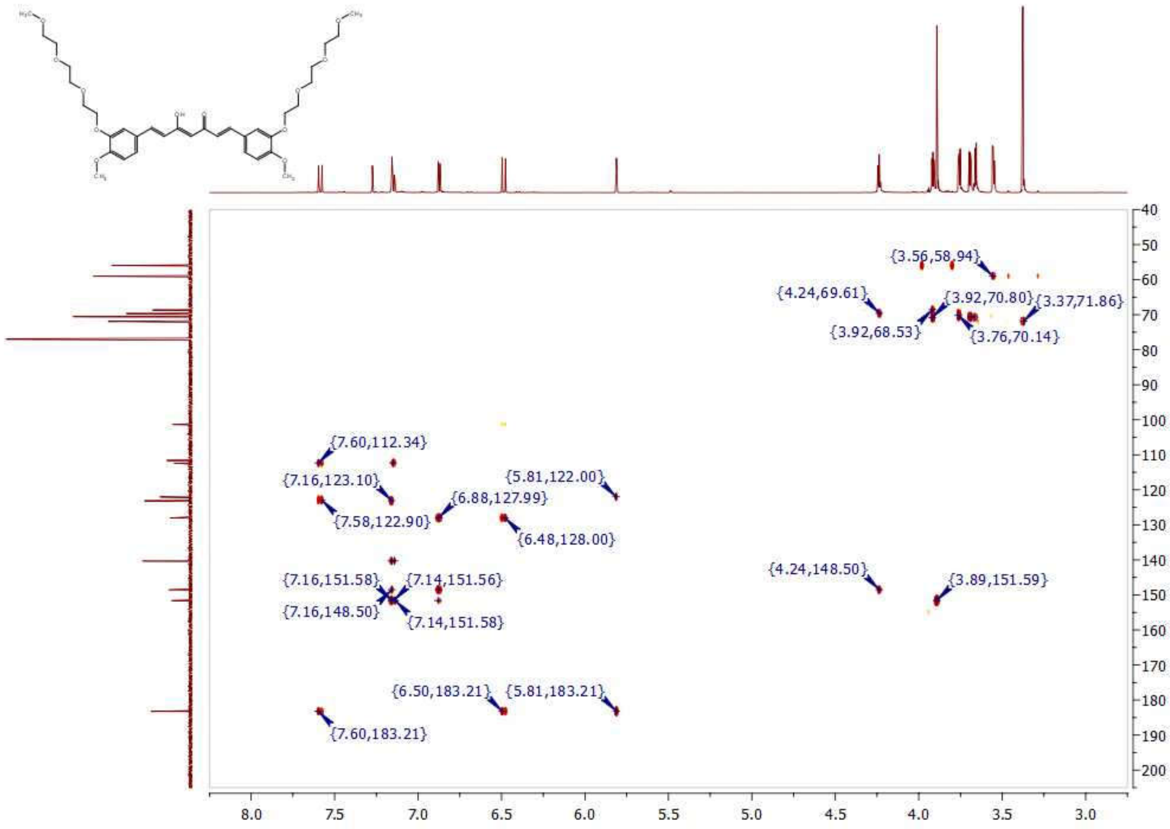

Figure S35. ^{1}H-^{13}C HMBC spectrum of compound **7**.

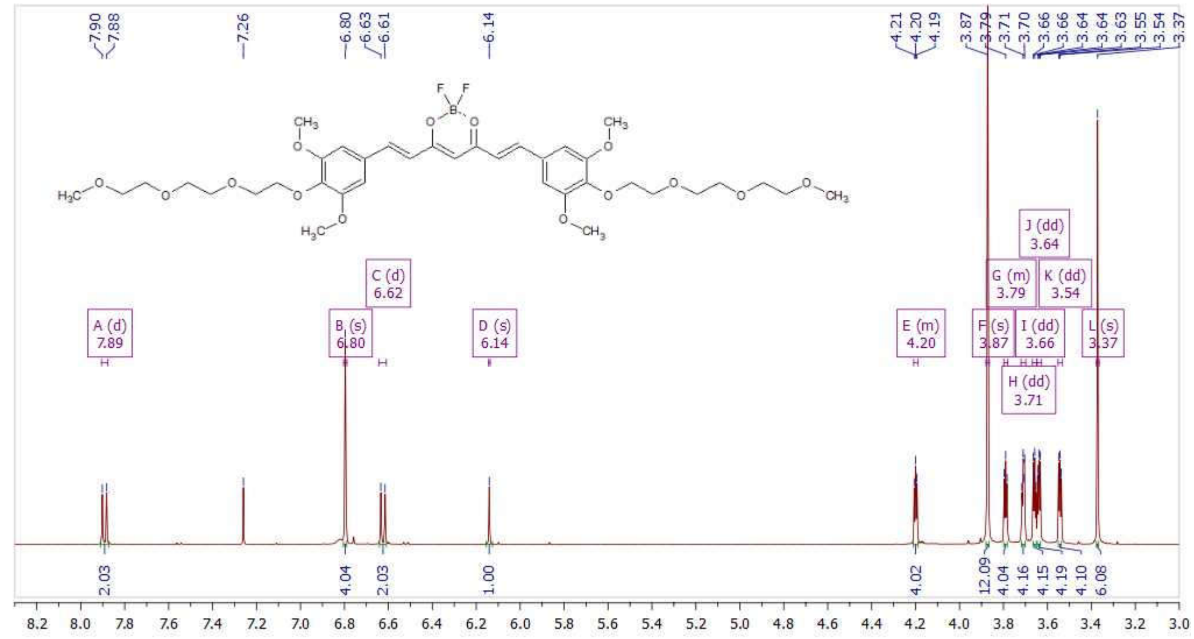

Figure S36. ^{1}H NMR spectrum of compound **8**.

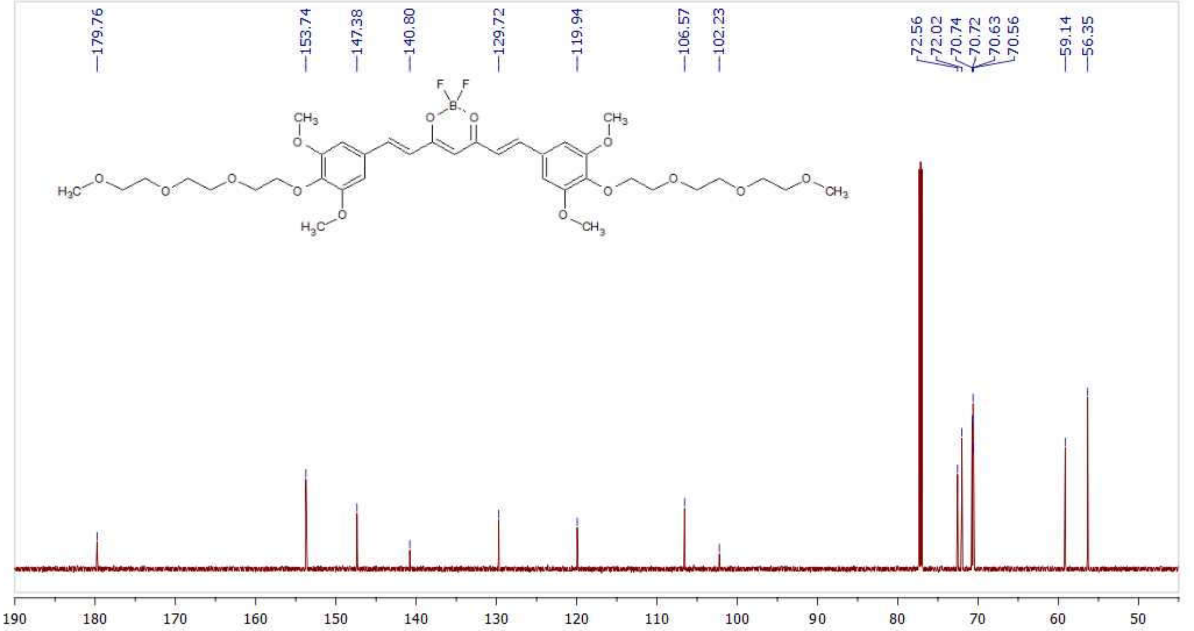

Figure S37. ^{13}C NMR spectrum of compound **8**.

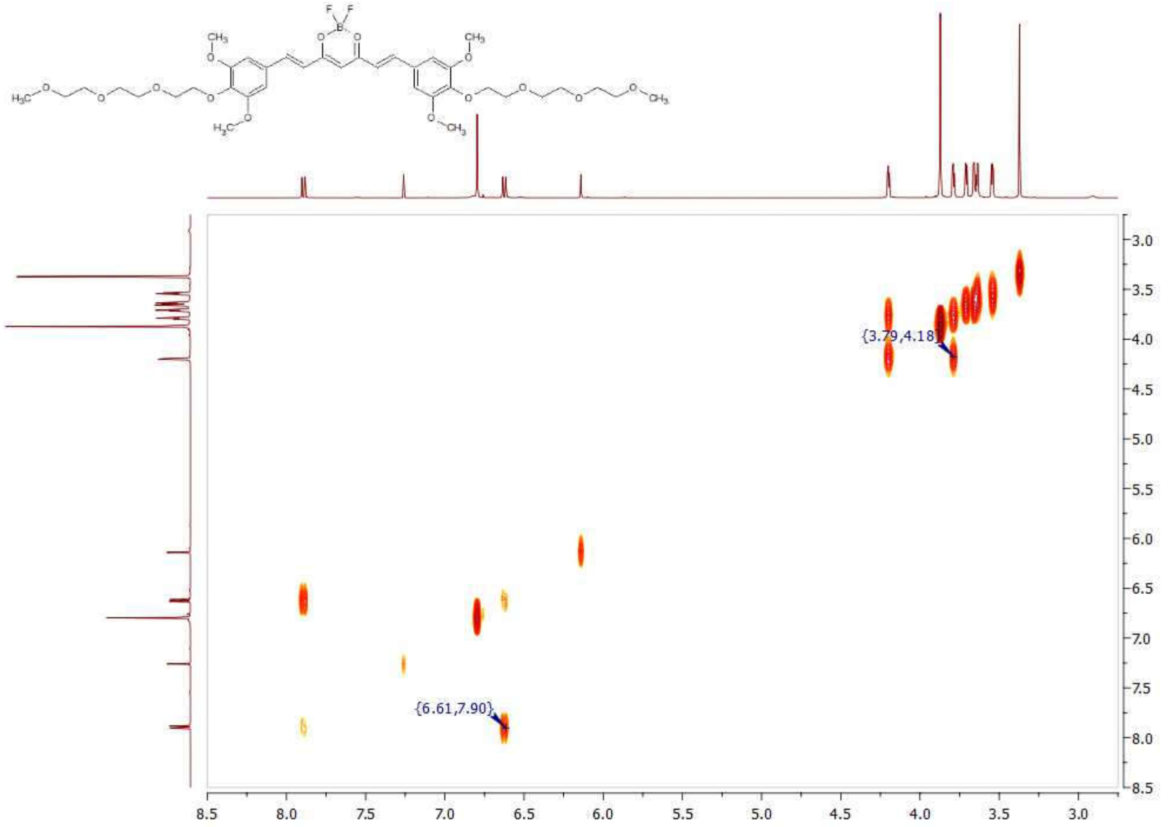

Figure S38. ^{1}H-^{1}H COSY spectrum of compound **8.**

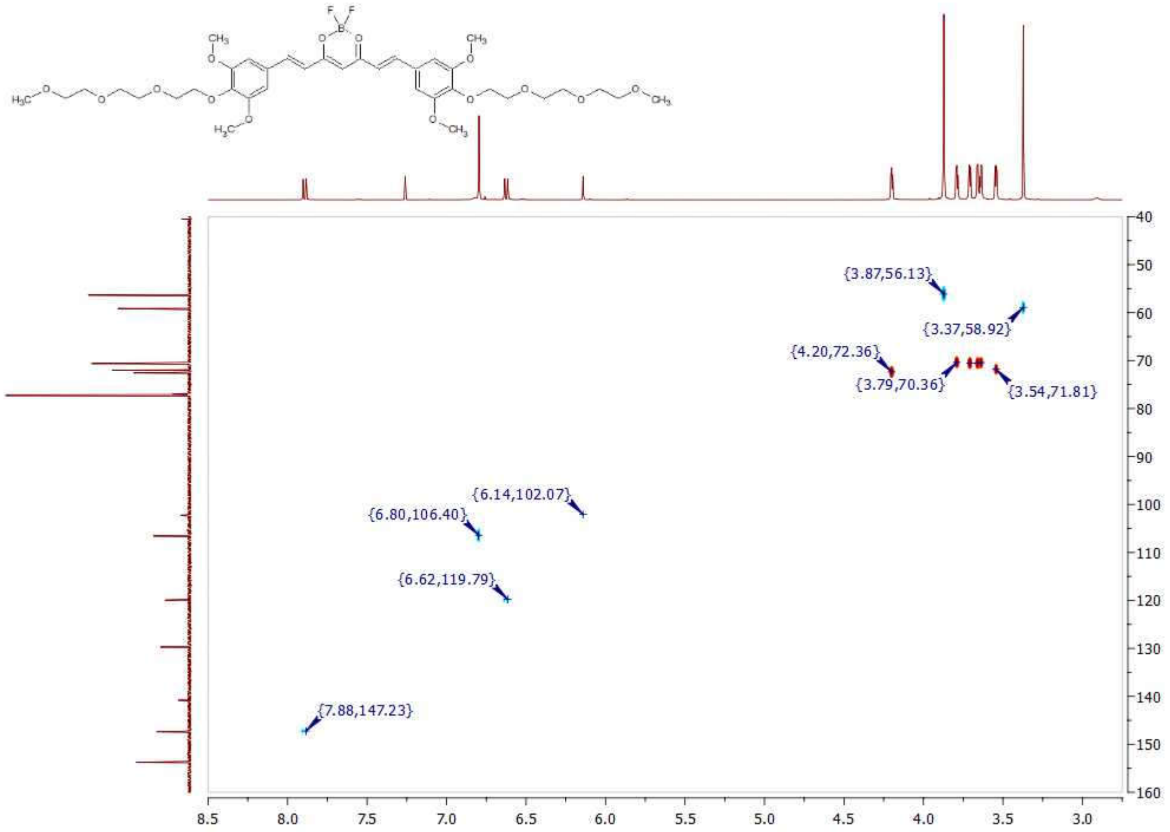

Figure S39. ^{1}H-^{13}C HSQC spectrum of compound **8.**

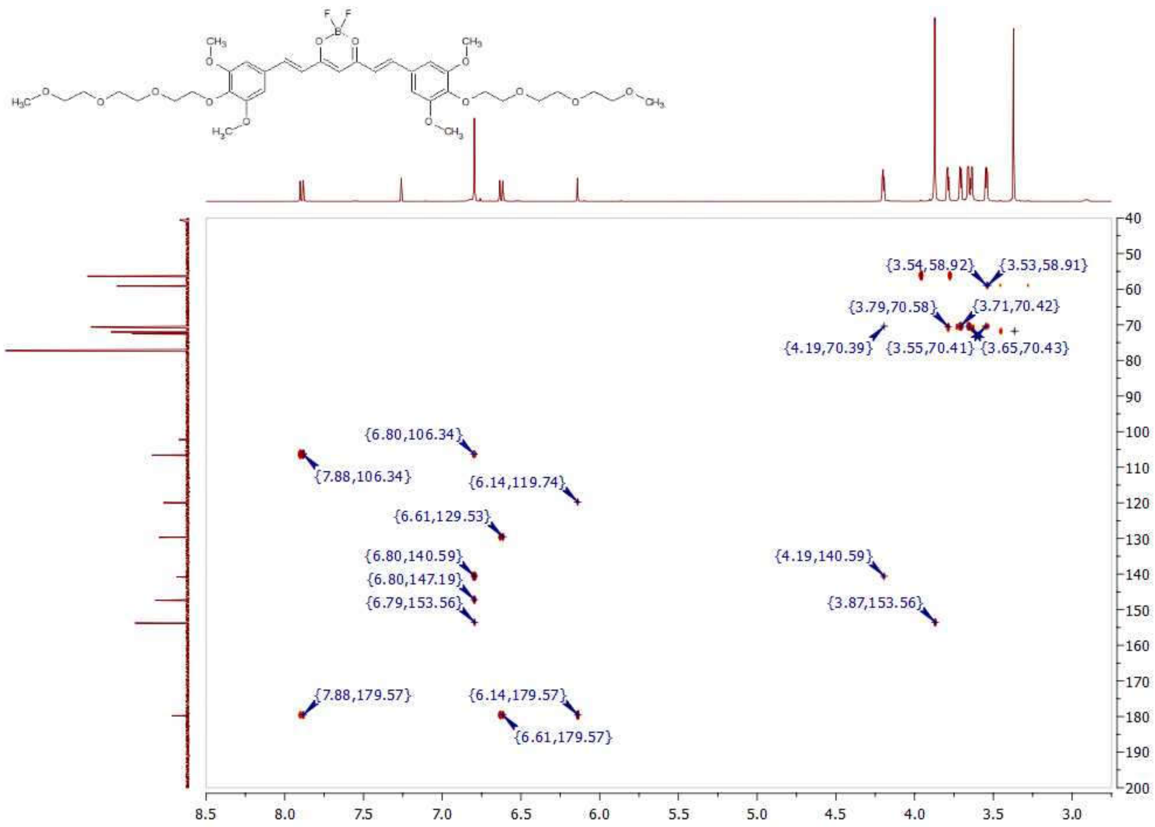

Figure S40. ^{1}H-^{13}C HMBC spectrum of compound **8.**

S2 – Mass spectrometry

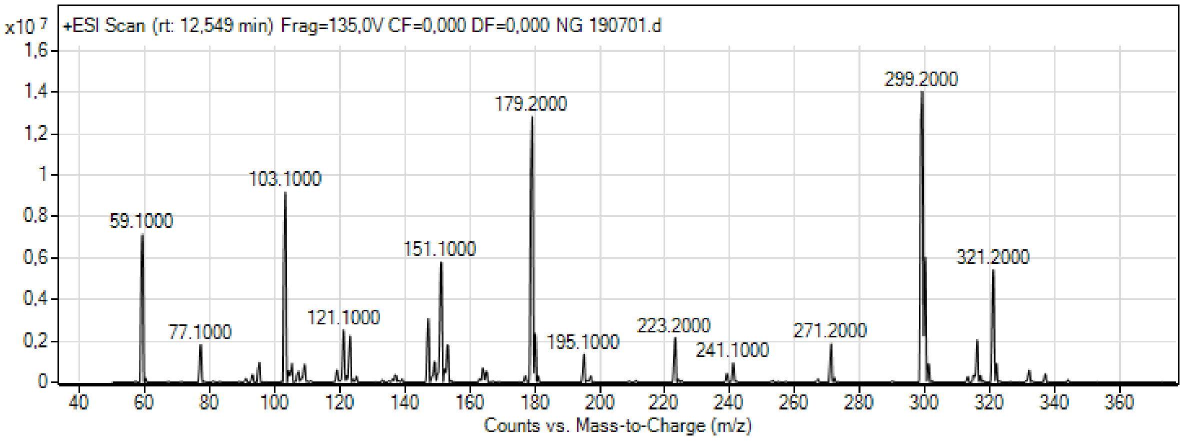

Figure S41. Mass spectra of aldehyde **1**.

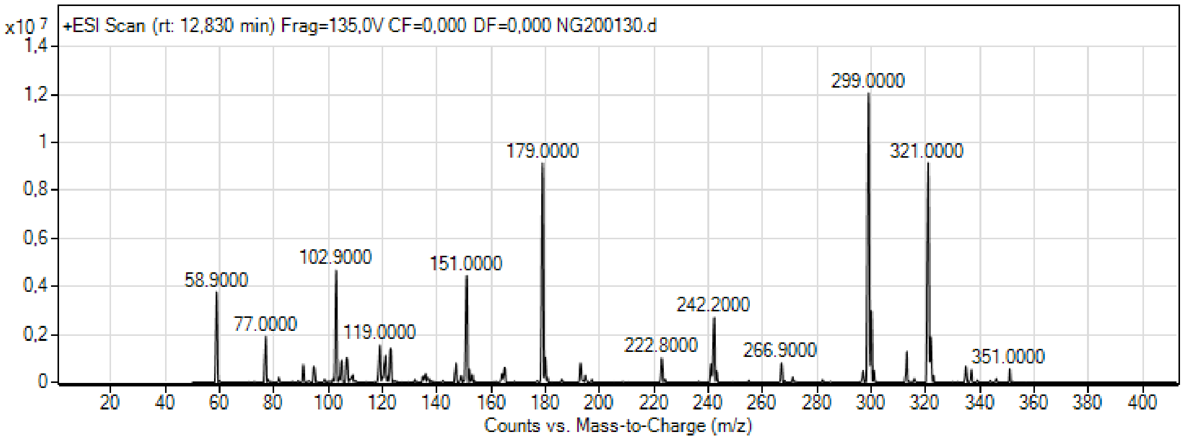

Figure S42. Mass spectra of aldehyde **2**.

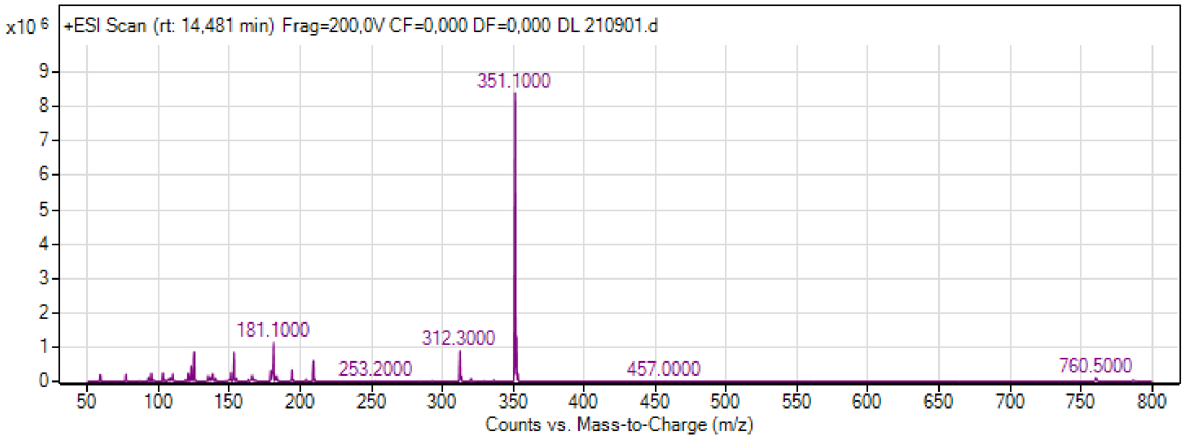

Figure S43. Mass spectra of aldehyde **3** sodium adduct.

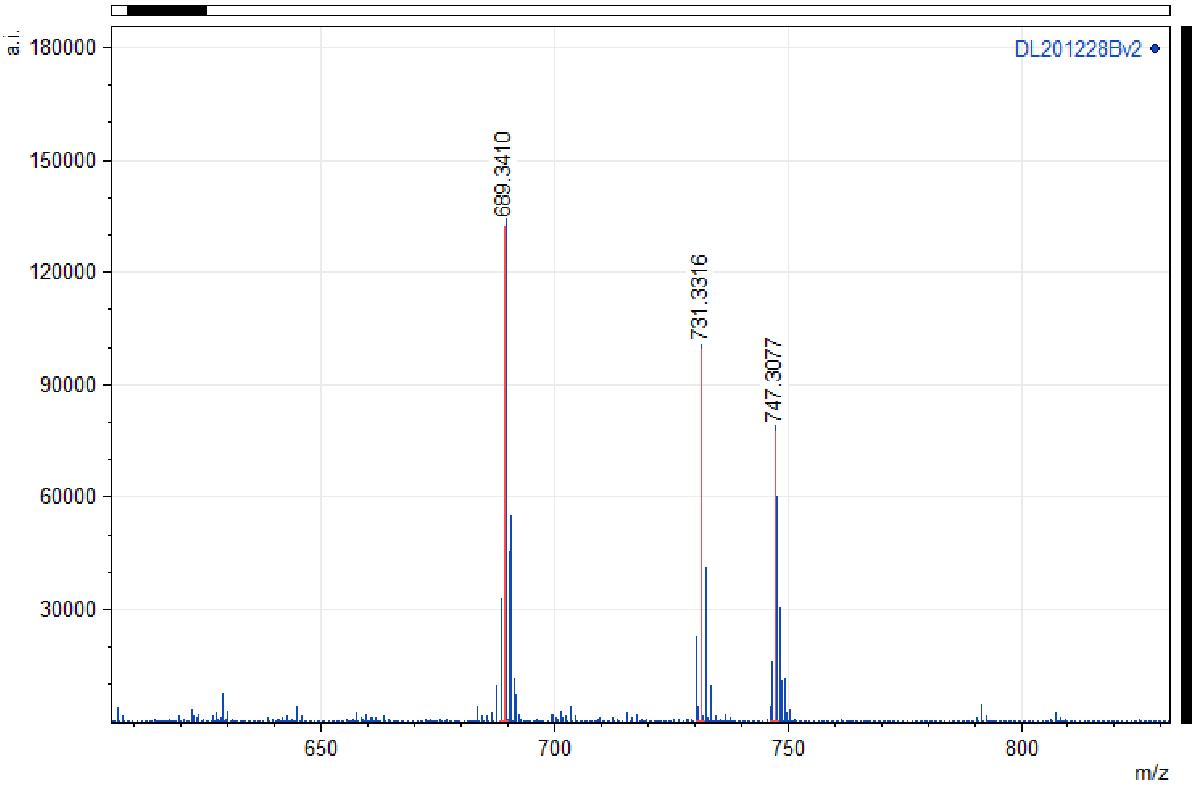

Figure S44. Mass spectrum of compound **4**.

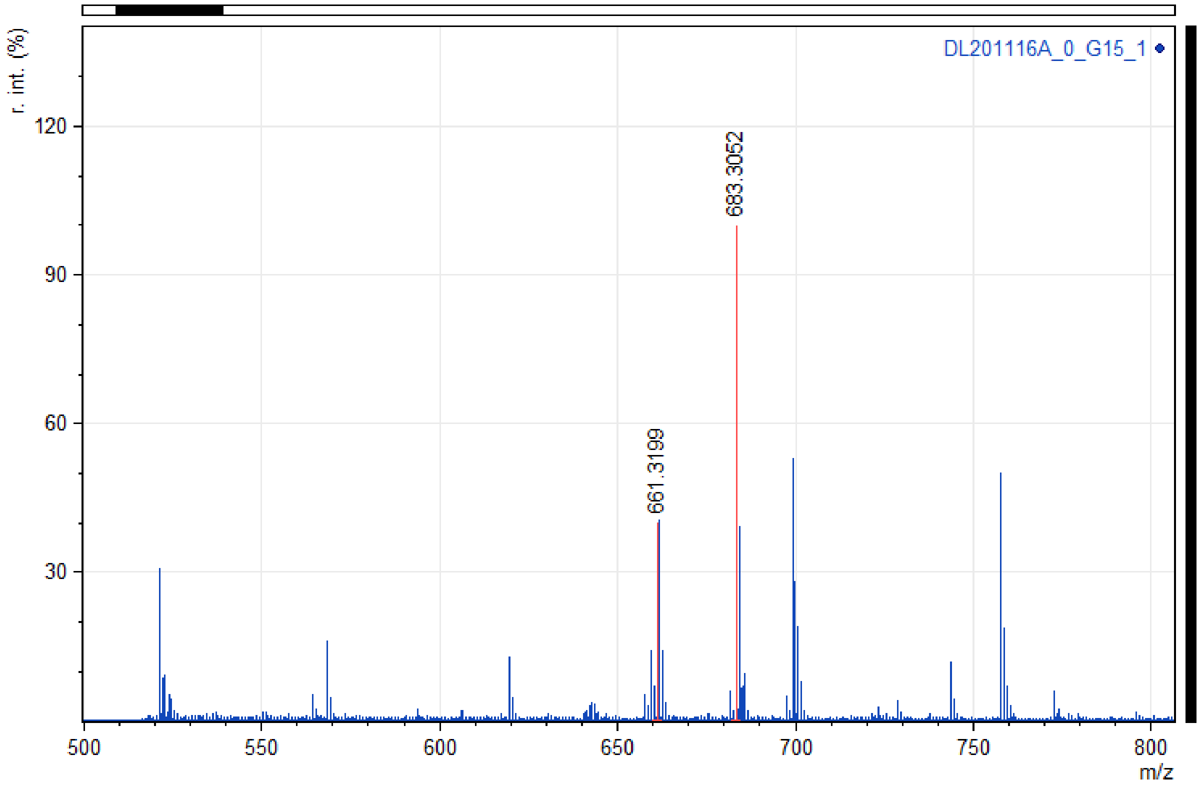

Figure S45. Mass spectrum of compound **5**.

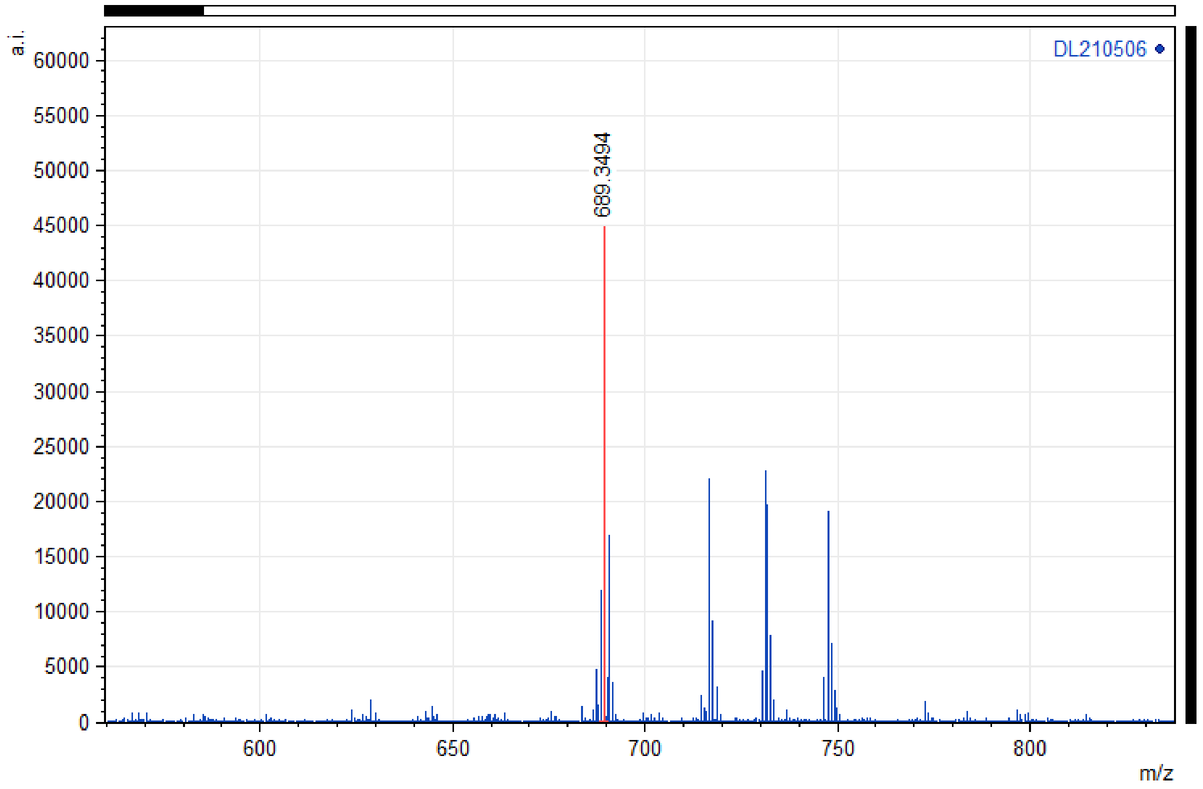

Figure S46. Mass spectrum of compound **6**.

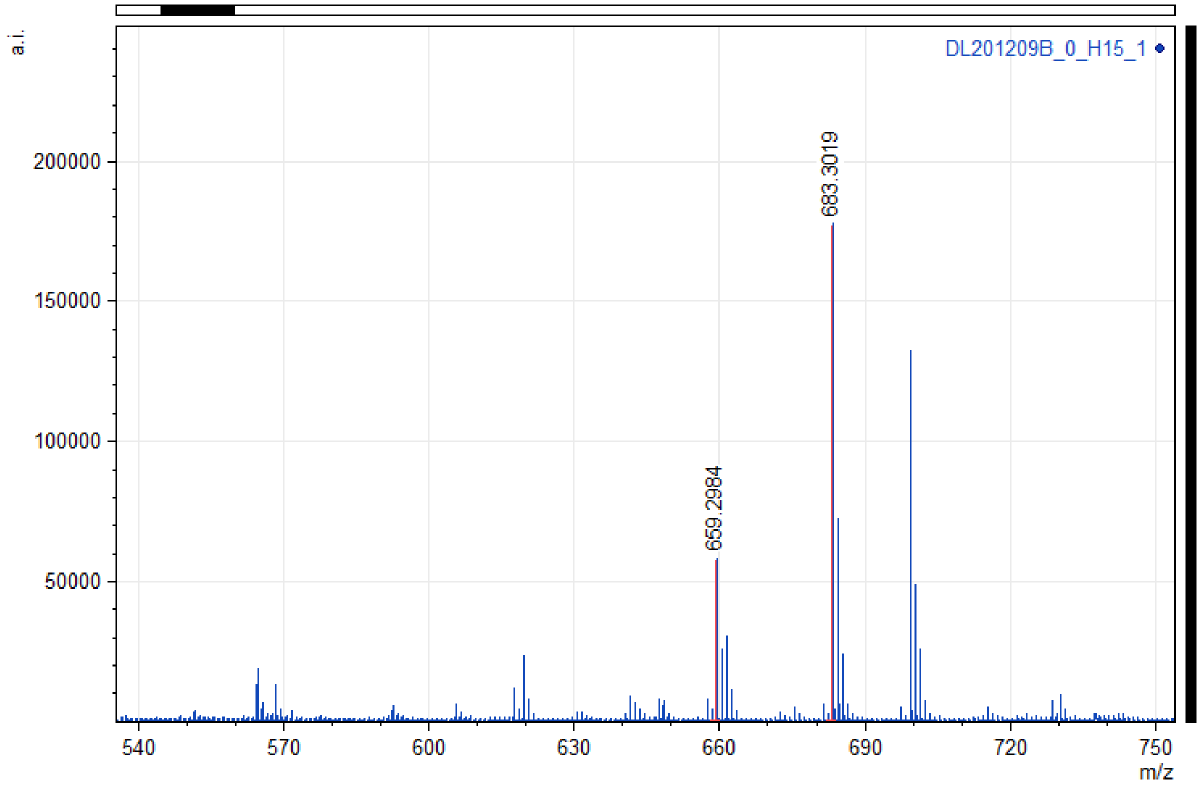

Figure S47. Mass spectrum of compound **7**.

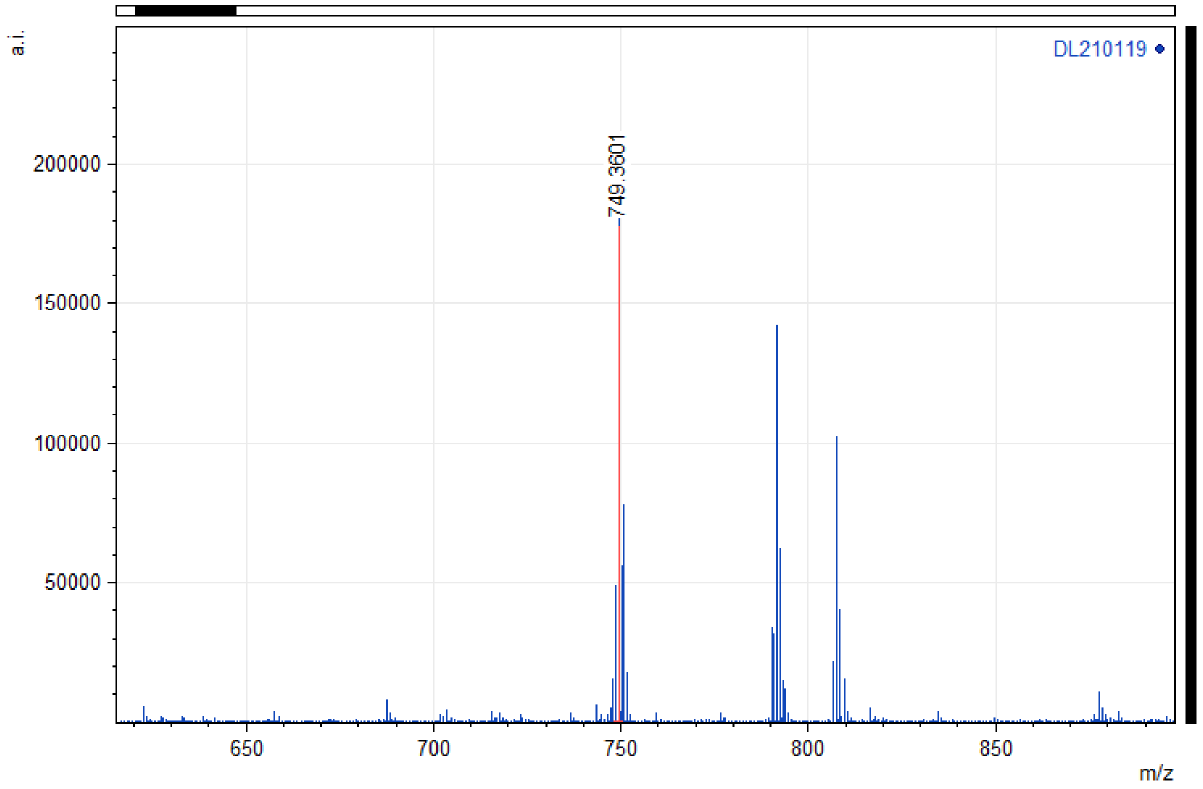

Figure S47. Mass spectrum of compound **8**.

S3 – UV-vis spectroscopy

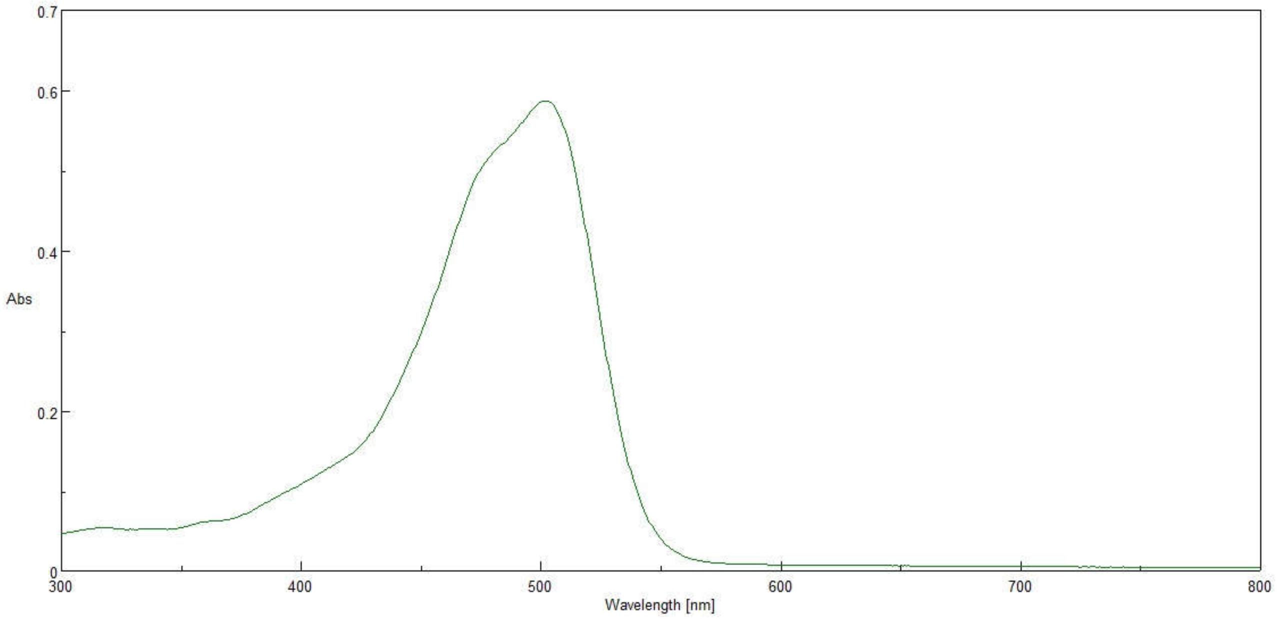

Figure S8. UV-vis spectrum of compound **4** in chloroform.

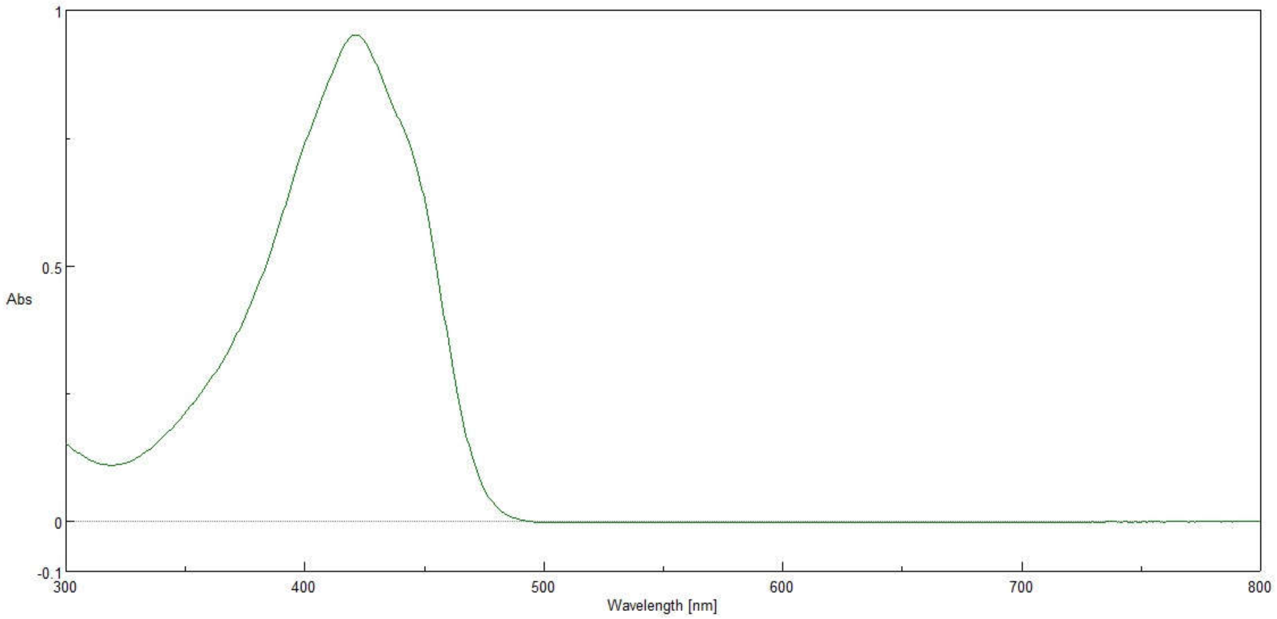

Figure S9. UV-vis spectrum of compound 5 in chloroform.

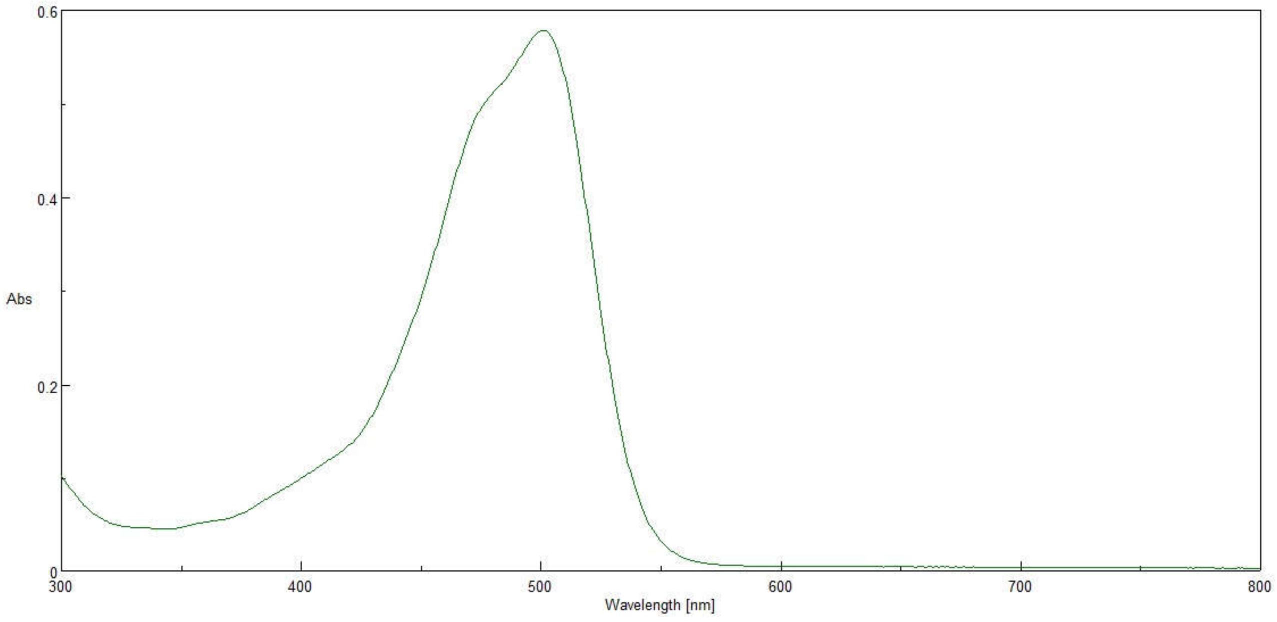

Figure S10. UV-vis spectrum of compound 6 in chloroform.

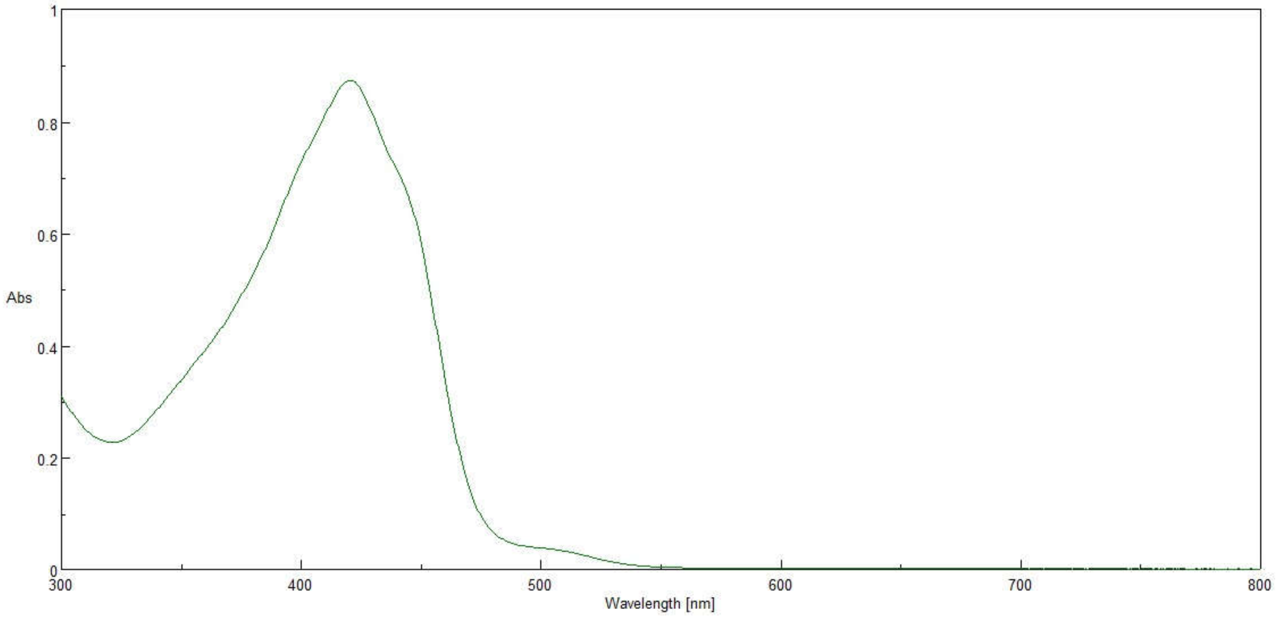

Figure S11. UV-vis spectrum of compound 7 in chloroform.

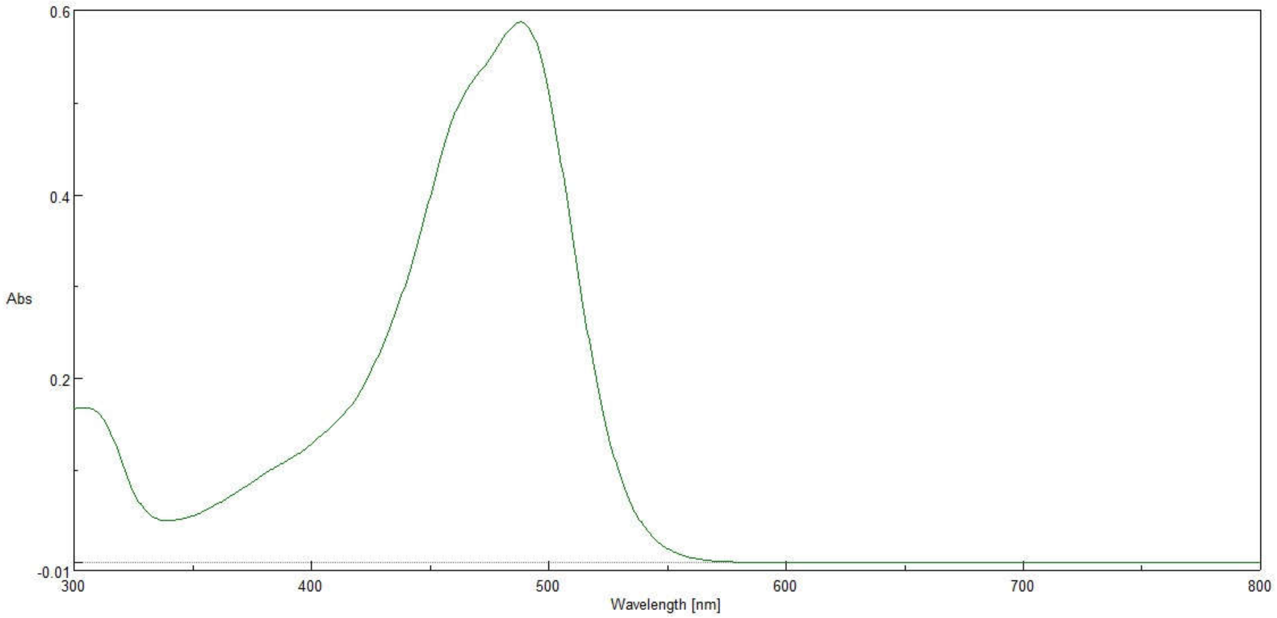

Figure S12. UV-vis spectrum of compound 8 in chloroform.

S4 – HPLC data

Table S1. HPLC gradient used for the analysis of new synthesized compound.

Time [min]	Phase A [%]	Phase B [%]
0	25	75
7	60	40
25	70	30
28	100	0
35	25	75

Table S2. Purity data for synthesized curcuminoids.

Symbol	C, mg/ml	λ_{max}, nm	t_R, min	%
4	0.2	491	11.21	97.47
5	0.2	418	12.04	97.38
6	0.2	502	12.11	99.38
7	0.2	418	12.77	97.20
8	0.2	480	12.62	98.90
10	0.2	502	9.12	98.81
11	0.2	424	9.58	99.41
12	0.2	492	9.26	98.59
13	0.2	418	9.56	99.42
